# Disentangling factors affecting bacterial transcriptional regulatory network inference

**DOI:** 10.1016/j.isci.2026.116834

**Published:** 2026-07-16

**Authors:** Gaoyuan Li, Joshua T. Burrows, Xuwen A. Lou, Bernhard O. Palsson, Daniel C. Zielinski

**Affiliations:** 1 Department of Bioengineering, University of California, San Diego, La Jolla, CA, USA; 2 Department of Pediatrics, University of California, San Diego, La Jolla, CA, USA; 3 Bioinformatics and Systems Biology Program, University of California, San Diego, La Jolla, CA, USA; 4 Center for Microbiome Innovation, University of California, San Diego, La Jolla, CA, USA; 5 The Novo Nordisk Foundation Biotechnology Research Institute for the Green Transition, Technical University of Denmark, Kongens Lyngby 2800, Denmark

**Keywords:** TRN inference, machine learning, transcriptional regulation

## Abstract

The rapid growth of bacterial gene expression databases has enabled computational inference of transcriptional regulatory networks (TRNs), yet it remains unclear why mathematically simple models often capture their apparent complexity. Using a 1035-sample *E. coli* expression database, we identify two transcriptome principles that support successful TRN inference. First, regulons defined from measured binding sites show limited overlap in gene membership, consistent with statistical independence exhibited by many successful inference methods. Second, 21% of genes, or 877 genes, exhibit regulator “dominance,” in which expression strongly correlates with a single regulator activity and receives minimal contributions from other regulators under most conditions. We formalize these properties with quantitative metrics and provide a reference catalog of dominantly regulated *E. coli* genes. Regulator dominance explains differences between expression-inferred and binding site-defined regulons, and removing dominated genes sharply reduces inference performance, suggesting that simply regulated promoter subsets are central to effective TRN inference.

## Highlights

• Independence and dominance underlie successful bacterial TRN inference
• Dominance explains gaps between inferred and binding-site regulons
• 877 *E. coli* genes (21%) are dominated by a single regulator

## Graphical abstract

**Figure:**
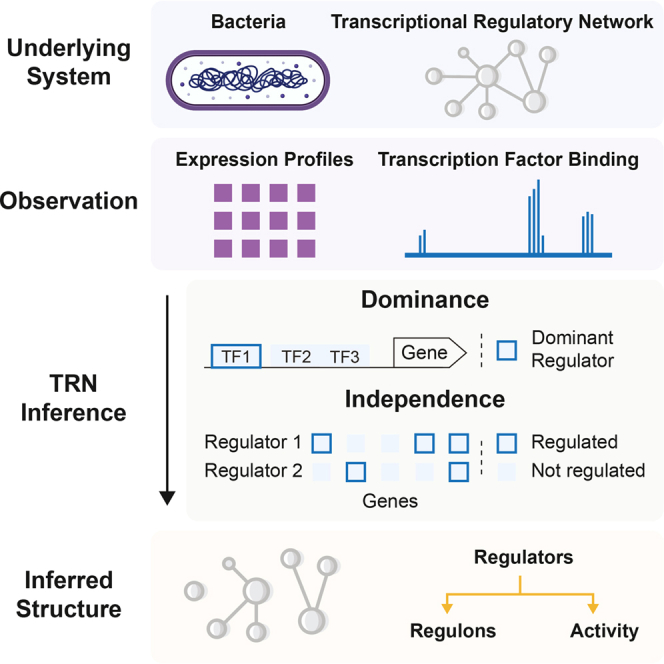

## Teaser

Biological sciences; Microbial genomics; Mathematical biosciences; Systems biology

## Introduction

The transcriptional regulatory network (TRN) of bacteria is a primary system of physiological control.^1^ TRNs for various microbes have been painstakingly defined component by component from the “bottom up” using molecular-biology assays. By directly testing regulator-DNA binding and regulatory effects, these studies formed the high-confidence backbone of bacterial TRN knowledge. More recently, chromatin immunoprecipitation experiments in regulator-perturbed strains under regulator-activating conditions^2^^,^^3^^,^^4^ have expanded coverage at the genome scale. These methods often reveal many more binding sites than are found to be functional, and assigning functional binding sites relies on the statistical testing of differential expression. As an additional challenge, the TRN that results from these experiments is a static network representation that lacks information on condition-specific regulator activities. Methods have been developed to directly measure condition-specific transcription factor (TF) activity through fluorescence, mass spectrometry, or multiplexed assays, but these methods have not yet been widely applied.^5^^,^^6^^,^^7^

As an alternative approach, “top-down” TRN inference attempts to determine regulatory relationships directly from large-scale data, most often transcriptomic profiles.^8^^,^^9^^,^^10^ Methods for TRN inference can be divided into three major categories. The first set of methods (which we term “Network-only methods”) focuses solely on identifying regulator-target gene connections, without directly inferring regulator activities.^11^^,^^12^^,^^13^^,^^14^ A second set of methods (which we term “Network-and-Activity methods”) simultaneously infer both the network structure and regulator activities, providing insights into both regulatory interactions and condition-specific dynamics.^15^^,^^16^ A third set of methods (which we term “Activity-only methods”) estimates regulator activities using a pre-established network structure as an input.^17^^,^^18^^,^^19^^,^^20^

Among the network-and-activity TRN inference methods, one particularly successful framework has been matrix factorization,^16^ which generates a linear regulatory model of expression (X = MA) consisting of a regulon structure matrix (M) and a regulatory activity matrix (A). In particular, independent component analysis (ICA), which maximizes the statistical independence of components, was shown to have the highest average test score out of 42 methods for transcriptional module detection.^21^ ICA-computed regulons have been termed iModulons, or independently modulated sets of genes.^22^ iModulons have been demonstrated to be consistent in many cases with experimentally annotated binding sites and regulator binding site sequences,^22^^,^^23^ and they have been used for a variety of discovery and synthetic biology applications.^24^^,^^25^ ICA is also a widely utilized processing step in the analysis of single-cell expression datasets.^26^^,^^27^

As described in the previous paragraph, the success of ICA and certain other inference methods for capturing bacterial regulon structure and activity has been demonstrated in previous works^22^^,^^23^^,^^28^^,^^29^ but is still not well understood. What properties of inference methods underlie their success? How can apparently complex transcriptional regulation be captured within relatively simple model structures, e.g., linear models that sum regulator contributions? In this work, we explore these questions and attempt to explain the success of bacterial TRN inference methods. We first argue that there is a theoretical basis for a linear model to capture nonlinear regulation in cases where only a single regulator is important. Then, we explore the properties of the *E. coli* TRN and expression states and find that these single-regulator cases are relatively common due to two complementary properties, which we find are critical to successful TRN inference. The first property is regulon *independence*, or a tendency for regulons to have minimal overlap. The second property is regulator *dominance*, in which a gene is controlled primarily by a single dominant regulator in the majority of conditions, even if additional regulators may be annotated. These properties have been previously observed in human TRN,^30^^,^^31^^,^^32^ and bacterial TRN,^33^^,^^34^ but their role in module inference algorithms has not been described. We find that observed differences between top-down and bottom-up regulons can be explained by alternative regulators exhibiting dominance on different genes within the regulon. Overall, our findings suggest that independence and dominance, as properties of bacterial transcriptional regulation, are key to the accurate modeling and inference of complex TRNs.

## Results

### Conceptualizing the transcriptional regulatory network inference challenge

**Figure 1:**
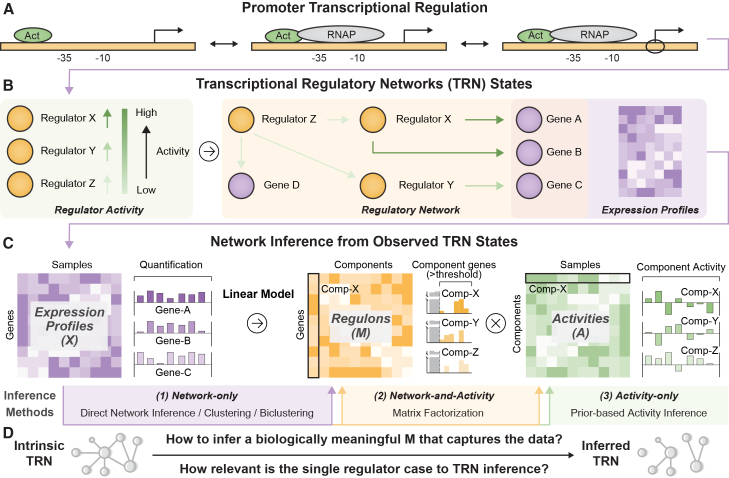
Overview of the prokaryotic transcriptional regulatory network and the formulation of network inference methods (A) Simple biophysical kinetic mechanism for transcription and its regulation by an activator (single strong regulator). Please see Note S1 for more details. (B) Overview of the underlying transcriptional regulatory network. Prokaryotic TRNs are formed by regulators (yellow) and their target genes (purple). The regulatory dynamics of these networks are characterized by the activity states of TFs (green). We observe the TRN state by expression profiling (purple matrix). (C) Formulation of linear modeling and inference method categories. Models begin with a gene expression matrix X (purple) and capture the regulatory relationship in M (yellow) and the regulator activity in A (green). Computational approaches for TRN inference are categorized as: (1) network-only methods that only identify regulatory interactions without TF activity; (2) network-and-activity methods that infer both the network and TF activity; and (3) activity-only methods that infer TF activity with a prior network structure as input. Note: These distinctions are only approximated based on the primary use of the tools in the literature. (D) Open questions to be answered in the following sections. The intrinsic TRN is the true biological regulatory network inside the cell, a ground-truth TRN. The reference TRN is the regulator-to-target-gene relationships from bottom-up experimental evidence (here, RegulonDB v14.5.0). The inferred TRN is the regulatory graph recovered top-down from expression data by an inference method.

To begin our investigation of how TRN inference methods can capture TRN structure and activity, we examine a simple model of how transcriptional regulation works at the promoter level. Prokaryotic transcriptional regulation modulates gene expression to a significant extent through controlling recruitment of the RNA polymerase to gene promoters by TFs and sigma factors (Figure 1A).^35^^,^^36^^,^^37^ Considering all promoters simultaneously, the bacterial TRN can be viewed as a “convolution” of two elements: the regulons that comprise the structure of the regulatory network and the condition-specific activity of corresponding transcriptional regulators (Figure 1B). The condition-specific state of the TRN is captured through expression profiling, which reflects a nonlinear combination of these elements.

TRN inference methods then attempt to deconvolute these observed gene expression states back into the TRN structure and regulator activities, most commonly using a linear model that separates regulator contributions additively (Figure 1C). In the case of matrix decomposition, mathematically this is written as decomposing a gene expression data matrix X into two matrices, M that contains modules of co-regulated genes, and A that contains the activities of those modules across samples in the dataset. As a basis for this deconvolution, it is possible to demonstrate a putative mechanistic relationship between the linear TRN inference model and the nonlinear transcriptional regulation model for cases of simple regulation, i.e., a single inhibitory TF (Note S1). However, while such relationships are possible under fairly strict assumptions, several key questions remain (Figure 1D). First, although some TRN structure matrix M may exist that effectively captures simple regulation, it is still not clear how successful TRN inference algorithms are able to find that M, i.e., compute an M that accurately captures experimentally determined regulons. Second, while it is expected from theoretical considerations that single regulator cases would be best captured by TRN inference, we do not know how frequently these single regulator cases occur, nor whether in practice these single regulator cases are important for successful TRN inference. In this work, we address these questions and, in the process, define regulatory principles that underlie the success of TRN inference methods (Please see Box 1 for definitions).

Box 1 **Definitions of core concepts**

• **Regulon:** a group of operons or genes controlled by one regulator.
• **Transcription factor (TF) activity:** the condition-specific functional state of a TF (active, repressing, inactive) that scales its regulatory effect on its targets.
• **Reference TRN:** the regulatory interactions established from bottom-up experimental data.
• **Inferred TRN:** the regulatory graph recovered top-down from expression data by an inference method.
• **Regulon Overlap:** the number of genes shared between two regulons. A gene is “in regulon overlap” if it is annotated as a target of two or more TFs.
• **Regulator Independence:** the property where the overlap between the regulons is minimized, meaning very few genes are shared across multiple distinct regulons.
• **Regulator Dominance:** the property where a gene’s measurable expression variance is driven primarily by a single, primary regulator across the vast majority of sampled conditions.
• **PRECISE-1K:** a 1035-sample, high-quality RNA-seq compendium from diverse growth conditions, including 9 media, 39 supplements, and 76 gene knockouts.
• **Component/iModulon:** a single column of the M matrix; a set of co-regulated genes identified by thresholding the values in an M column. Components produced by ICA specifically are called *iModulons* (independently modulated gene sets).

### Successful TRN inference methods exhibit higher statistical independence

The first question we explored was how TRN inference methods successfully approximate the TRN structure matrix M, i.e., compute an M that aligns with bottom-up regulon structure. We first benchmarked the performance of TRN inference methods in capturing regulatory interactions and activities. Network-only and Network-and-Activity methods were evaluated to assess their performance in recapturing the experimentally validated regulons. Resulting TRN structures were compared to the TRN from RegulonDB v14.5.0 (released January 2026; downloaded 2026-04-27), a manually curated transcriptional-regulation resource that integrates evidence from classical experimental approaches, high-throughput binding assays, and non-experimental sources. To evaluate whether Network-and-Activity methods can reliably estimate the activity matrix A from the expression data X, we used activities inferred from RegulonDB v14.5.0 TRN using NCA^17^ and decoupleR.^20^ Because direct ground-truth activities are generally unavailable, we treated agreement between these TRN-informed estimators as a practical proxy for benchmarking against the closest available reference. The two methods were highly consistent, showing an average pairwise correlation above 0.8, supporting their use as robust reference activity estimates (Figure S2A). Components from network-and-activity methods were enriched for regulators using their binarized M-matrices, with the top enrichment being considered for comparison. Each regulator that had an enrichment for a given method was then correlated with the activities for all enriched components of a method, with the highest correlated component being considered as the corresponding component.

**Figure 2:**
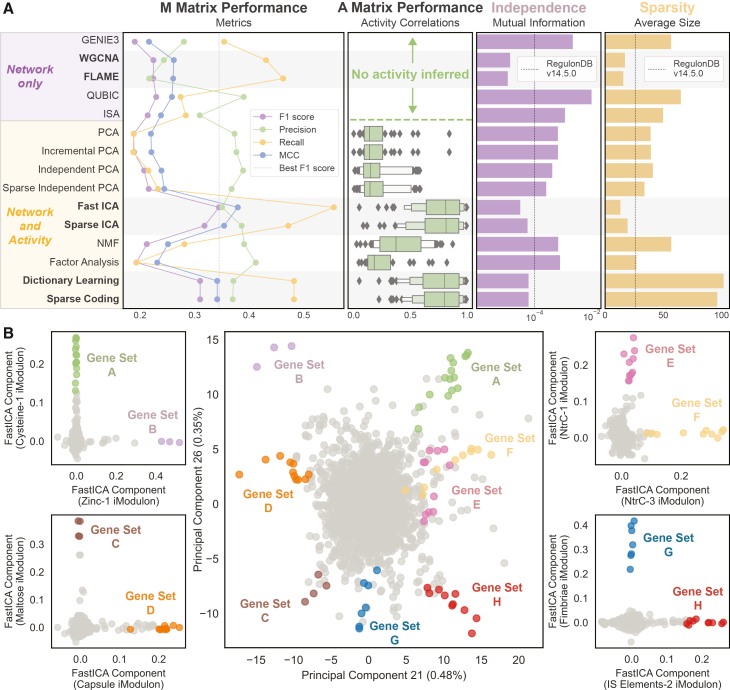
Benchmark and numerical properties of prokaryotic transcriptional regulatory network inference methods (A) Benchmark for inference methods. Average precision, average recall, average F1 score, and average Matthews correlation coefficient (MCC) were used for the M matrix benchmark. Please see the methods section for more details. For the network-only methods, we evaluated GENIE3^13^ (direct network inference) WGCNA^12^ (clustering), FLAME^39^ (clustering), QUBIC^40^ (biclustering), and ISA^41^ (biclustering). GENIE3 and WGCNA were chosen for their widespread use, while FLAME, QUBIC, and ISA were selected for their strong performance and ability to accommodate overlapping genes across components, as highlighted by Saelens et al.^21^ A total of ten matrix factorization methods were examined covering principal component analysis^42^ (PCA), independent PCA,^43^ sparse independent PCA,^43^ incremental PCA,^44^ FastICA,^45^ sparse ICA,^46^ non-negative matrix factorization^47^ (NMF), factor analysis,^48^ dictionary learning,^49^ and sparse coding.^49^ Results from the PRECISE-1K dataset are presented here. The number of components for all these methods was set to 250 to be roughly consistent with the number of regulators with strong evidence (S) and confirmed (C) evidence from RegulonDB^50^ v14.5.0. Please see the Methods section for the definitions of strong and confirmed evidence from RegulonDB. This selection ensures comprehensive coverage of widely adopted dimensionality reduction and feature extraction techniques, providing a robust benchmark for assessing TRN inference methods. Additional dimensions and benchmarks on *B. subtilis* are in Figures S3–S15. The M matrices obtained from these methods were binarized following the approach described by Sastry et al.^22^ The methods are evaluated based on their ability to accurately capture regulons from RegulonDB v14.5.0 (see methods section for details). The activities (A matrix) of Network-and-Activity methods are evaluated by measuring the correlation between the inferred activities from these methods and those from Activity-Only methods. Results from decoupleR ULM are presented here. The average mutual information and sparsity of the M matrices from various TRN inference methods were calculated. The values from RegulonDB experimental TRN were plotted on the vertical dashed lines as a reference. (B) Scatterplot of the gene weights of two principal components from PCA, selected for having top mutual information. The surrounding plots are the independent components corresponding to sets of genes within the two principal components highlighted in the center PCA plot. Gene Set A: cysJ, cysD, cysI, tsuA, yciW, cysH, cysK, tsuB, cysP, cysA, cysN, cysU, cysC, tcyP, cysW; Gene Set B: ykgO, ykgM, zinT; Gene Set C: lamB, malM, malK, malE; Gene Set D: gmm, yjbE, rcsA, wcaA, wcaE, wcaF, wzb, wza, wzc, gmd, fcl; Gene Set E: ddpX, ddpA, ddpB, ddpC, ddpD, ddpF, astD, astB, astA, astC, astE; Gene Set F: rutA, rutE, rutG, rutF, rutB, rutC, rutD, yhdX, yhdY, yhdW; Gene Set G: fimI, fimD, fimA, fimC, fimF, fimG, fimH; Gene Set H: insD1, insD6, insC1, insC6, insH21, insH9, insH4, insA4, insA6, and insC5.

Here we evaluated 15 methods (Note S2) using the *E. coli* transcriptomic compendium PRECISE-1K.^38^ The results demonstrated that FastICA, Sparse ICA, Dictionary Learning, Sparse Coding, WGCNA, and FLAME delivered superior performance across key metrics, including recall, F1 score, and MCC. Notably, FastICA achieved the highest values in all three metrics, outperforming the other evaluated approaches (Figure 2A). The benchmark results are consistent across different dimensions, different datasets, and different confidence levels for the reference TRN (Figures S3–S15). The top-performing methods of FastICA, Sparse ICA, Dictionary Learning, and SparseCoder in the M matrix also displayed the highest correlation corresponding components to the TRN-based activity inference methods (Figures 2A and S2B). Under conditions where the regulator was deleted, such as in the Fur or Cra knockouts, the inferred regulator activities consistently showed extreme values. This aligns with the expectation that deleting a key regulator removes its direct effect on gene expression, leading to a marked shift in the inferred activity and thereby validating the method’s reliability (Figures S16A and S16B). From the above assessments, it is clear that several methods perform at a qualitative level above other inference methods at capturing the *E. coli* TRN structure and activity. Based on its high performance across evaluations, FastICA was selected for the following sections as the exemplary inference method. Similarly, Saelens et al.^21^ highlighted FastICA as the top-performing method in their module detection benchmark. To put the cross-method comparison on an absolute footing, we report the fraction of v14.5.0 regulators (≥3 annotated members, *n* = 169) that FastICA recovers at increasing best-match F1 thresholds: 50.3% at F1 ≥ 0.4, 37.9% at F1 ≥ 0.5, and 17.2% at F1 ≥ 0.7, with a macro-averaged F1 of 0.41 across the 169 regulators. This is a moderate, not saturating, level of recovery and is consistent with the existing literature that ICA is the strongest of many imperfect methods, rather than that ICA is itself complete.

To understand the factors underlying the observed superior performance of certain TRN inference methods, we examined two key metrics that are commonly utilized as criteria by the inference methods: mutual information, which measures independence, and the average number of genes per component, a metric of the sparsity of the components (Figure 2A). These metrics were chosen based on the observation that ICA-based methods tend to perform well, and these methods tend to exhibit both high independence and sparsity, leading us to examine whether one or both of these properties are key to their success. Indeed, FastICA and Sparse ICA result in components with low mutual information, indicative of high independence, and high sparsity with few genes per component. However, Dictionary Learning and SparseCoder also demonstrate high independence but produce less sparse components, yet they still effectively capture regulons from RegulonDB. These results suggest that limiting regulator overlap (i.e., achieving independence) is the critical factor influencing performance, rather than focusing on reducing the total number of regulatory relationships (i.e., sparsity).

To better understand why independence would be a useful criterion in capturing regulon structure, we contrasted two similar and well-understood methods: principal component analysis (PCA) and ICA. While both PCA and ICA compute components that are mathematically orthogonal, ICA finds components that maximize statistical independence, and PCA finds components that maximize explained variance. The difference between orthogonality and statistical independence is subtle, but fascinatingly, we found that this difference appears to be a key distinction underlying the success of ICA at capturing biological regulons. We demonstrate this later in discussion.

To examine the practical relevance of these mathematical details, we utilized the PCA results computed above and selected two principal components (PCs) with high mutual information, i.e., low statistical independence, as well as 8 independent components (ICs) from ICA that shared gene membership with these PCs (Figure 2B). It can be seen that the two PCs, despite being mathematically orthogonal, are effectively mixed signals of the 8 ICs, showing a significant overlap in gene membership between the two PCs that is not seen in the corresponding 8 ICs. It thus appears that since PCA prioritizes explained variance, it tends to merge distinct but related gene sets into fewer components. In comparison, FastICA, which ensures both orthogonality and statistical independence, separates gene sets with subtle differences into different components (Figure 2B). Despite this separation, FastICA maintains a similar total explained variance to PCA (Figure S17), highlighting its ability to uncover distinct biological modules without sacrificing explanatory power. As another point of comparison, we also compared components from NMF, which is another method previously used to infer the TRN of *E. coli*,^51^ to FastICA components, and we once again found that mixed signals from NMF could be separated into ICs (Figures S18A and S18B). This distinction underscores the importance of independence in disentangling complex biological signals and providing a clearer representation of the modular organization in gene expression data.

Due to its success by metrics examined thus far, we selected FastICA as an exemplary inference method for the remaining analysis conducted in this work.

### A substantial fraction of genes is dominantly regulated by a single transcriptional regulator

**Figure 3:**
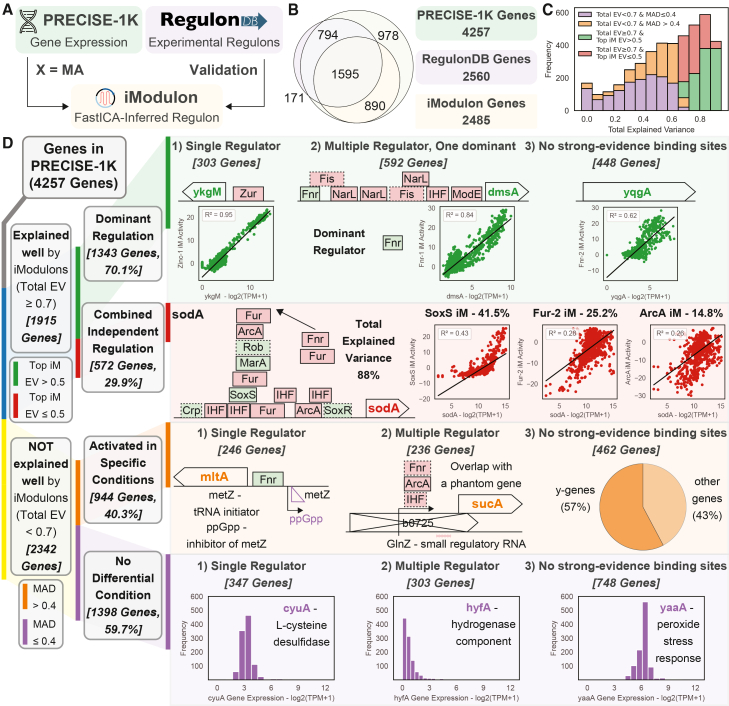
Categorization of genes in PRECISE-1K (A) Workflow overview. The iModulons were inferred from the PRECISE-1K dataset using FastICA. Experimental binding site information was obtained from RegulonDB. (B) Venn diagram for genes in the PRECISE-1K dataset, genes with experimental binding site annotation in RegulonDB, and genes that are in iModulons. (C) Histogram of the total explained variance by FastICA decomposition of all the genes in PRECISE-1K. 0.7 was selected as the cutoff to determine if a gene is well-explained by iModulons or not. (D) Sankey diagram for the categorization of genes in PRECISE-1K. Genes with a total explained variance (EV) greater than 0.7 were considered well-explained, with further distinctions based on the top iModulon EV: genes with a top iModulon EV above 0.5 were categorized as dominant regulation, while genes with a top iModulon EV of 0.5 or less were attributed to combined independent regulation. Genes with a total EV of 0.7 or less were considered not well-explained, and their classification depended on the MAD: genes with MAD above 0.4 were identified as activated in specific conditions, whereas those with MAD of 0.4 or less were deemed to show no differential condition.

Theoretical considerations suggest that inference should work best in cases of simple regulation; we thus sought to determine whether a link exists between the transcriptional regulation complexity of a gene and the ability of ICA to capture its expression regulation. We utilized the extensive available gene expression and regulon data for *E. coli* to assess the complexity of transcriptional regulation across *E. coli* genes. RegulonDB^50^ serves as a bottom-up experimental reference, providing knowledge on regulatory complexity such as the number of regulatory sites at each gene at certain confidence levels (Figure 3A). For the PRECISE-1K^38^ dataset of 1035 *E. coli* expression profiles, FastICA captures 58.4% of the genes within iModulons (Figure 3B). We constructed a decision tree to identify metrics that best distinguish between genes captured by iModulons and those that are not. The variance explained by the iModulon with the highest contribution (Top iM explained variance) and mean absolute deviation (MAD) were selected as key explanatory features (Figures S19 and S20). In the PRECISE-1K dataset (mostly minimal media for single-substrate growth), iModulons explain the expression of 1,915 genes with an overall explained variance greater than 0.7 (Figure 3C). For the remaining 2,342 genes, either the genes do not exhibit substantial variation (possibly due to no regulation), or ICA fails to capture their regulation for a number of possible reasons as discussed later in discussion.

Among the 1,915 well-explained genes, 1,343 have a dominant regulator, where the top iModulon explains more than 50% of the expression variance (Figure 3D). Here, we define dominance when a single regulator has primary control over gene expression, while other regulators either do not bind simultaneously or, if bound, have minimal or no effect on gene expression. We call this regulator the dominant regulator for that gene. These 1,343 well-explained genes fall into three categories (Figure 3D, denoted in green). First, 303 genes are regulated by a single regulator, making their expression variance straightforwardly explained by one component, such as *ykgM*. Second, 592 genes are regulated by multiple regulators, as annotated in RegulonDB, but still have their expression variance largely explained by a single regulator. For instance, the *dmsA* gene, encoding a subunit of the dimethyl sulfoxide reductase, despite having eight potential regulatory binding sites, shows strong correlation with the Fnr-1 iModulon (R^2^ = 0.84), highlighting the dominant influence under the conditions sampled here even in complex regulatory scenarios. Third, 448 genes without strong evidence for binding sites have their expression variance effectively explained by a single component. One likely explanation is indirect dominance, where the dominant regulator is correlated with the true direct regulator through a feedforward or regulatory cascade. For example, the *yqgA* gene, which lacks high-confidence binding site annotations, is strongly correlated with the Fnr-2 iModulon (R^2^ = 0.62). This suggests that Fnr likely regulates these genes despite their absence from RegulonDB annotations, probably through indirect dominance (Figure 3D). These findings highlight dominant regulation as a key part of *E. coli* gene transcriptional regulation, accounting for 31.5% of the genes.

Next, we identified a group of 572 genes with high overall explained variance by ICA that contributed to multiple ICs, which we categorized as “Combined Independent Regulation.” 177 (30.9%) of the genes in this category have over 0.7 total explained variance just with the top three explainable iModulons. For example, the *sodA* gene, encoding a superoxide dismutase, is bound by 9 distinct TFs at its promoter at the all-evidence level (ArcA, CRP, FNR, Fur, IHF, MarA, Rob, SoxR, SoxS; 17 binding sites in total, 5 distinct TFs and 11 sites at the Confirmed + Strong confidence level), and 88% of its expression variance under PRECISE-1K conditions is explained by iModulons. However, this variance is primarily distributed among three ICs: the SoxS iModulon (41.5%), the Fur-2 iModulon (25.2%), and the ArcA iModulon (14.8%) (Figure 3D, denoted in red). This indicates that the complex regulation of the *sodA* gene arises from the combined contributions of SoxS, Fur, and ArcA. Other regulators in the promoter region have minimal impact under these conditions. These findings suggest that simple additive regulator contributions underlying a linear model such as ICA can, in some cases, capture substantial regulator interactions; however, we observe that these cases are a small minority of cases.

As mentioned earlier, 2,342 genes are not well explained by iModulons. Using MAD, we categorized them into two groups: those activated under specific conditions and those with no differential expression. Among the 944 genes with high MAD (Figure 3D, denoted in orange), 462 genes are activated under specific conditions but lack regulatory annotations, with 57% being y-genes (Figure S21A). Another 246 have a single regulator but are influenced by additional factors, such as *mltA*, whose expression is mostly explained by the ppGpp iModulon due to interference from the nearby *metZ* tRNA initiator, which is inhibited by ppGpp (Figure S21B). Additionally, 236 genes have multiple regulators but are affected by complex contexts, such as *sucA*, which overlaps a pseudogene and includes a small regulatory RNA GlnZ binding site. The only confirmed binding regulator, ArcA, contributes 23% of the total explained variance due to interference (Figure S21C). These examples illustrate how local interference and regulatory complexity limit ICA’s ability to fully capture gene regulation. For the 1,398 genes with low MAD, their expression shows little variability across the PRECISE-1K compendium (Figure 3D, denoted in purple). Examples from this group indicate that these genes maintain consistent expression levels, even when annotated with binding sites. This observation may be due to the absence of activating conditions in the PRECISE-1K dataset or the possibility that the binding sites annotated in RegulonDB are non-functional. In conclusion, it appears that ICA performs excellently at capturing the expression of a substantial minority of genes that are dominated by a single regulator; however, ICA fails to effectively capture expression variation of a majority of genes with either complex regulator interactions or insufficient expression variation.

### Transcriptional regulator independence and dominance underlie the ability of ICA to capture regulon structure

**Figure 4:**
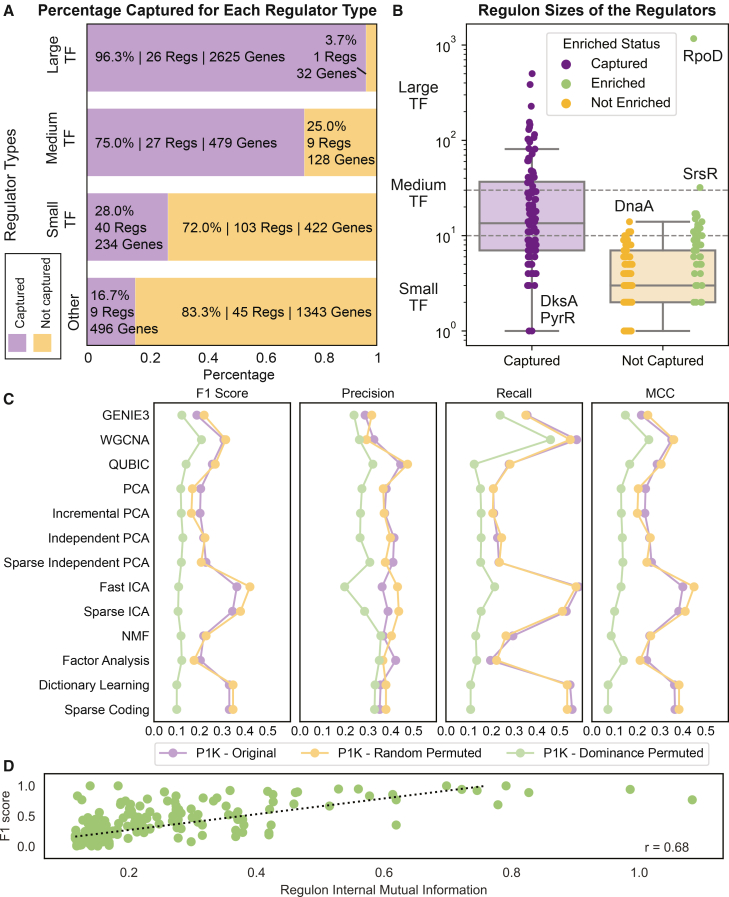
Regulator Capture in PRECISE-1K (A) Stacked bar chart detailing regulators which are captured (denoted in purple) and not captured (denoted in yellow) in PRECISE-1K and their regulator type, as well as putative regulators captured by PRECISE-1K. The categories were given by the size of the regulon: Small TF (regulon size <10), Medium TF (10 ≤ regulon size ≤30), and Large TF (regulon size >30). (B) Box and strip plots representing the size of captured regulators in PRECISE-1K against those that are not captured. (C) Line charts of the F1 score, precision, recall, and MCC on capturing regulons of different inference methods on original PRECISE-1K (P1K - Original), and a randomly selected gene set (877 genes in total) permuted PRECISE-1K as controls (P1K - Random Permuted), and regulatory-dominated genes permuted PRECISE-1K (P1K - Dominance Permuted). FLAME and ISA were excluded from the benchmark in Figure 4C because they do not allow the user to specify the number of components; obtaining an exact component count would require manual parameter tuning for each run, which is not feasible across all the random permuted groups. (D) Scatterplots of F1 score of each regulon versus the average pairwise mutual information among all genes within each regulon, measuring internal regulatory coherence.

Based on the observation that the expression of many genes is tied to a single regulator, we sought to examine whether regulon capture by ICA is influenced by properties of regulator dominance and independence. Based on the iModulon structure inferred from PRECISE-1K and RegulonDB, we identified regulators associated with each ICA component (iModulon). Across the dataset, a total of 260 regulators from the RegulonDB TRN database were divided into 102 captured and 158 not-captured regulators (Figure 4A). The median size of captured regulators was 13.5 regulated genes, while for all not-captured regulators the median was 3 regulated genes, indicating that all large regulators, except RpoD and SrsR, were captured. Among not-captured regulators, 117 were not significantly enriched in gene presence for any component, while 41 were significantly enriched in at least one component, including SrsR and RpoD, although neither was the most significant enrichment for any component (Figure 4B). Many small TFs are not captured, indicating that they likely are either too small to be reliably captured or do not possess activating conditions in the PRECISE-1K dataset. We found that iModulons generally capture regulons with high precision, indicating efficient capture of core regulation members, while recall was often lower, especially for large regulons, indicating incomplete regulon capture (Note S3 and Figure S22A). We also found in certain cases that ICA can capture more complex regulator interactions and hierarchies, but these form a minority of cases (Note S3 and Figure S22B). These results suggest that FastICA performs generally well at capturing regulons of sufficient size, and non-captured regulators appear primarily due to a combination of the limited number of robust components found by the algorithm and limitations of the conditions measured in the expression dataset.

We then examined whether the independence or dominance of the regulators is associated with the ability of ICA to capture their regulons. We found that for medium and large regulons, the independence of the regulon positively correlates with the ability of ICA to capture its structure. We also found that regulator dominance correlates with the ability of ICA to capture its structure for small regulons (Figure S23). To further examine the role of dominated genes on ICA performance, we identified the 877 dominantly regulated genes (Table S1) and shuffled these genes randomly, as well as 10 sets of randomly selected genes. Critically, we observed that regulon capture performance dramatically decreased when perturbing regulator-dominated genes, while perturbing random sets of genes had no effect on performance (Figures 4C and S24). This also holds true for other methods benchmarked in the study, as all the methods have a significant performance drop when regulator-dominated genes are permuted (Figure 4C).

Following up on the result that precision is high but recall can be low for large regulons, we computed the mutual information of genes within the part of regulons captured by ICA, and found that this mutual information correlated positively with regulon capture performance (Figure 4D). We also found that overall, captured regulons tend to have higher gene-gene correlation for all the genes within the regulon (Figure S25). These results suggest that the expression concordance within a regulon is a key feature for TRN inference performance, and ICA appears to capture the subset of a full regulon that has the highest mutual information in gene expression changes. Thus, it appears that, while at the regulator level inference methods perform well across broad sizes of regulons, at the gene level inference methods primarily capture regulation of the subset of genes whose regulation is dominated by a single regulator within the dataset. This subset of dominated genes thus can be interpreted as acting as biomarkers of the *in vivo* activity of their regulator. These results place important bounds on the capabilities of TRN inference methods and motivate improvements to these algorithms.

### Dominant regulation explains differences between binding site-determined and expression-inferred regulons

**Figure 5:**
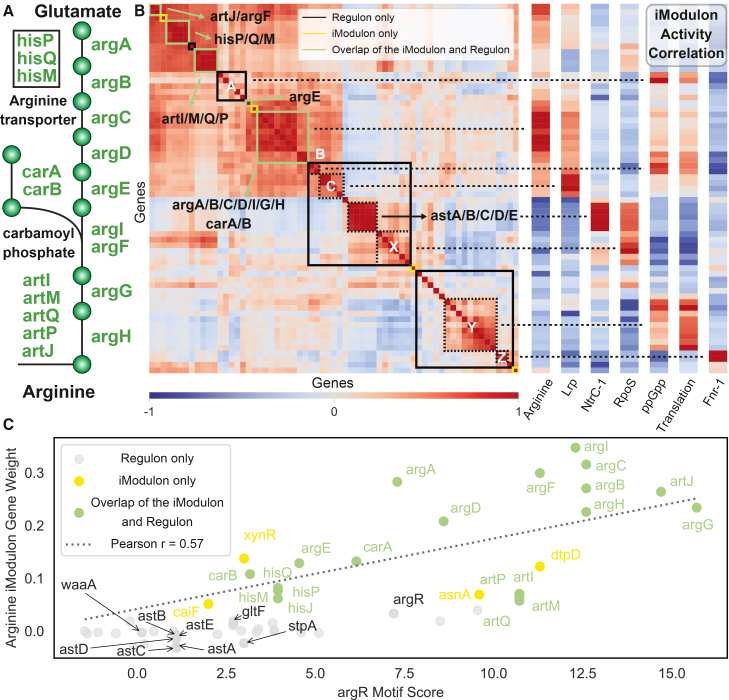
The ArgR regulon consists of gene clusters dominated by diverse regulators (A) Metabolic pathway for arginine biosynthesis contained within the Arginine iModulon. (B) Correlation heatmap of expression profiles for genes in the ArgR regulon and Arginine iModulon. X axis and Y axis both represent genes. The color scale represents pairwise correlation coefficients, ranging from -1 (blue) to 1 (red). The order of the genes is given by hierarchical clustering (average distance) on the gene expression correlation matrix. The black blocks highlight genes exclusive to the ArgR regulon, the yellow blocks indicate genes unique to the Arginine iModulon, and the green blocks highlight genes shared by both the Arginine iModulon and the ArgR regulon. Genes in cluster A include yraQ, waaA, stpA, argR, and gltF. Genes in cluster B include lysO and cvpA. Genes in cluster C include gltD, gltB, aroP, and lrp. Genes in cluster X include ykgA, hchA, dacC, dps, ftsZ, and pfkB. Genes in cluster Y include aroK, rpsO, ydgI, rimP, pnp, infB, nusA, truB, and rbfA. Genes in cluster Z include yhcC, yfcC, and gltP. The right part shows the correlation between the activities of the iModulons and the gene expression profiles shown. Arginine, Lrp, NtrC-1, RpoS, ppGpp, translation, and Fnr-1 are plotted here. (C) Scatterplot of arginine iModulon gene weights and ArgR-binding site motif log-odds score.

Results in the previous section suggest only a subset of large regulons are typically captured by ICA (i.e., low recall), and that the part of the bottom-up regulon being captured within an iModulon is the set of genes that share high expression mutual information. In this section, we build on this result by demonstrating, using the ArgR regulon and Arginine iModulon, that many differences between bottom-up and top-down regulons can be explained by diverse regulator dominance at individual promoters within the regulon. Strikingly, the Arginine iModulon neatly captures the entire biosynthetic pathway from glutamate to arginine, covering both enzymes (argA to argH) and transport systems (hisP, hisQ, hisM, and artI to artJ) (Figures 5A and S26). However, the ArgR TF has a much larger binding site-determined regulon, which prompts investigation into what drives the difference between the Arginine iModulon and the broader ArgR regulon.

Based on the results from Figure 4D, we hypothesized that different parts of the ArgR regulon were coordinated differently. To examine this, we computed gene expression correlations among the members of the ArgR regulon, which revealed a clear clustering pattern of co-regulated genes (Figure 5B). The Arginine iModulon predominantly captures a set of genes in the top-left cluster, which exhibits a high expression correlation both within the gene set as well as with the activity of the Arginine iModulon. Notably, the genes that are included in the iModulon have significantly higher ArgR binding site motif scores, suggesting these genes contain the strongest ArgR binding sites (Figure 5C). Thus, it appears that the part of the ArgR regulon that is captured by the Arginine iModulon is the set of genes most strongly regulated by ArgR. There are additionally four genes that appear in the Arginine iModulon but not in the ArgR regulon, but each of these cases is explainable based on promoter context (Note S3 and Figure S27).

However, for the remainder of the ArgR regulon, we found that other regulator-associated iModulons were more highly correlated with the genes than the Arginine iModulon, suggesting that an alternative regulator was the dominant regulator at these other promoters. Within this large upper-left cluster, there are three sub-clusters of genes that the Arginine iModulon does not include, which we denote as Clusters A, B, and C. Cluster A contains the ArgR gene, which encodes the repressor of arginine biosynthesis, so it is not expected to be in the Arginine iModulon. The other genes in this cluster are more highly correlated with the activity of the ppGpp iModulon (Figure 5B), and their argR-binding motifs have lower binding affinity than those in the Arginine iModulon (Figure 5C). In clusters B and C, the genes are more highly correlated with ppGpp and Lrp, respectively. For the gltB and gltD genes in cluster C, their expression profiles can be largely explained by the Lrp and Leucine iModulon (Figure S28). Importantly, regulation by Lrp at these promoters is reported in RegulonDB, lending credibility to the alternative dominant regulator. Thus, it appears that the non-Arginine iModulon genes in the upper-left gene cluster are influenced, but not dominated, by ArgR regulation.

In the lower-right section of the expression correlation heatmap, the Arginine iModulon is not well correlated with the expression of this subset of the ArgR regulon, and instead many genes are dominantly regulated by other regulators. For example, the expression of the astCADBE operon, which has binding sites for ArgR, CpxR, and NtrC, is dominantly controlled by NtrC (Note S3 and Figure S29). The remainder of the bottom right cluster forms distinct subclusters, which we label X, Y, and Z. Genes in cluster X are highly correlated with an RpoS-associated iModulon, genes in cluster Y are highly correlated with ppGpp and translation iModulons, and genes in cluster Z are highly correlated with an Fnr iModulon (Figure 5B). Once again, in each of the cases where an alternative regulator appears dominant, there is binding site evidence in RegulonDB for the activity of that regulator at the corresponding promoter.

We found support that these differences between bottom-up and top-down regulons are similarly explainable beyond the case of ArgR; for example, the FlhDC regulon and CysB regulon also have genes that appear dominated by other regulators. Similarly, these regulons include genes that are highly correlated with other genes but not identified as part of the RegulonDB regulon (Figures S30 and S31). The dominance partitions here show consistency with the dominant-GO partition of Ledezma-Tejeida et al.,^52^ where FlhDC and CysB sit among the functionally homogeneous *E. coli* regulons. Taken together, these results indicate that both top-down and bottom-up regulons offer valuable and complementary information about regulon structure and activity; top-down analysis captures the most highly active part of the regulon that is dominated by the regulator, while bottom-up regulons appear more inclusive and contain regulatory relationships that may only matter for particular subsets of conditions.

## Discussion

TRN determination in bacteria has been a fundamental challenge in systems biology,^8^^,^^10^ with extensive efforts both to experimentally measure regulator DNA binding sites and to infer network structure from gene expression data. This challenge is likely greater in eukarya, where larger genomes, chromatin regulation, and distal regulatory elements increase the complexity of both experimental mapping and expression-based inference. We sought to determine how effective TRN inference is enabled by the properties of inference algorithms and gene expression data. Using a combination of mechanistic modeling, evaluation of inference methods, and analysis of gene expression data, we argue that regulator independence (few genes are in multiple regulons) and regulator dominance (genes with measurable expression variance are dominantly regulated by a single regulator under most sampled conditions) are properties exhibited by the subset of the bacterial TRN that is recoverable by inference methods.

Bottom-up biophysical modeling suggested that simple linear models can be expected to capture gene expression regulation well when, at a given promoter and under the conditions sampled, a single regulator is the functionally dominant one. Examination of real promoters often reveals many more binding sites per promoter on average, with an average of 3.42 TFs per promoter, even at a “high confidence” level that involves assessing that a regulatory event occurs. Thus, it is initially unclear how the linear model that underlies ICA and other inference methods can effectively capture expression variation. We proposed independence of regulators and regulator dominance at individual promoters as properties of regulons that facilitate their inference. Together, these conditions satisfy the criteria that only a single regulator is functionally active at a promoter, either due to lack of other regulators (independence) or only a single regulator being functionally significant in most conditions (dominance). Prior work^33^^,^^34^ has discussed these properties largely through selected case studies; here, we provide quantitative measures of independence and dominance for each gene and compile a catalog of dominantly regulated genes based on the largest single-organism RNA-seq compendium available to date.

We evaluated a set of inference methods and found several methods, including ICA, as standout performers, consistent with a larger previous analysis.^21^ Algorithms that performed well showed substantial variation in sparsity but generally high statistical independence. We note that independence is a distinct and stricter criterion than orthogonality, which is enforced in algorithms such as PCA and FastICA. We found that real regulons as determined by high-confidence binding sites in RegulonDB, showed relatively moderate sparsity but high independence, consistent with high-performing algorithms. Biologically, independent regulation is logical as it maximizes the modularity of regulation.^53^ This work suggests that methods mimicking real biological properties benefit in performance. However, balancing independence and overlap during inference is challenging due to the presence of global TFs and their context-specific regulation.^54^ There is still a significant minority of genes that appear in multiple regulons, including validated cases, and the biological implications of these overlapping regulons have not been fully explored.

Surprisingly, we observed that a large fraction (∼21%) of genes in the dataset had a dominant regulator as estimated by high correlation with a single ICA component. We found that this principle offered a convincing explanation for a long-standing mystery in the field regarding the differences between expression-inferred regulons and binding site-determined regulons.^55^ Specifically, within the ArgR regulon, the part of the regulon captured by ICA was strongly correlated with this signal; however, other parts of the regulon were instead correlated with the activities of other regulators at those promoters, indicating that while those genes may still have active ArgR binding sites, they have alternate dominant regulators. This raises a question about the physiological role of regulators such as ArgR at promoters where they are not the dominant regulator. Initial investigation indicates that ArgR is still the most important regulator in a minority of conditions even when it is non-dominant, as observed in its interactions with NtrC in this study and a previous study.^56^ Determining whether non-dominant binding plays a key role in adaptation in certain conditions remains a topic for further experiments. We also note that the apparent overall dominance exhibited by particular regulators depends on the breadth of the experimental conditions within the underlying gene expression database, and the relevance of these lab-developed conditions to real environments experienced by *E. coli* in the wild is unknown. Importantly, these results suggest that both top-down and bottom-up determined regulons contain valuable information about regulon structure and activity, and thus moving forward these methods could be seen as complementary as opposed to “approximate” and “gold-standard”, respectively.

This work combines multiple approaches to identify the drivers behind the success of TRN inference. We suggest that regulator independence and dominance are key properties of bacterial TRNs, which is consistent with the assumptions that enabled decoupling of regulatory relationships and regulator activity, as well as designs of best-performing algorithms. These results build confidence in computational inference methods through explaining their behavior, may help guide future algorithm development, and further justify the development of large expression databases to empower these workflows.

### Limitations of the study

Despite the success of the activity of dominant regulators at explaining the expression of many genes, the above statistics of dominant genes indicate that the majority (2,914) of *E. coli* genes still could not be explained by a single regulator. We believe this highlights a key property of ICA and inference methods in general, which is the difference between capturing regulation at the gene level versus capturing regulator activity. Only in a minority of cases is expression regulation captured well, mostly being these dominant regulation cases and a smaller set of 572 genes where linear addition of regulator signals sufficiently captures their interactions. However, we observed that the vast majority of large and medium (26 of 27), and some small (40 of 107), regulators are captured well by ICA. This indicates that sufficient genes exist from each regulator’s regulon that are dominated by the regulator in order to estimate that regulator’s activity. These inferred regulator activities have been validated through regulator knockouts and extensive experience.^22^^,^^28^^,^^29^ Thus, we expect inference methods such as ICA to work best at the regulon level, and only work well at the gene level for those genes whose regulation is dominated by a single regulator.

## Resource availability

### Lead contact

Daniel C. Zielinski (dczielin@ucsd.edu).

### Materials availability

This study did not generate new materials.

### Data and code availability

#### Data

All the data used for analysis in this study have been deposited at the GitHub repository (https://github.com/SBRG/Bacterial_TRN_Dominance_and_Independence) and are publicly available.

#### Code

All the code used for analysis in this study has been deposited at the GitHub repository (https://github.com/SBRG/Bacterial_TRN_Dominance_and_Independence) and is publicly available.

#### Additional information

Any additional information required to reanalyze the data reported in this paper is available from the lead contact upon request.

## Acknowledgments

We would like to thank our reviewers for their helpful comments.

This work was supported by the Novo Nordisk Foundation Biotechnology Research Institute for the Green Transition at the Technical University of Denmark (NNF24SA0100980) and the Novo Nordisk Foundation (NNF) Center for Biosustainability (CfB) at the Technical University of Denmark (NNF20CC0035580).

## Author contributions

Conceptualization and design of study: D.C.Z., G.L., and B.O.P.; data analysis and interpretation: G.L., D.C.Z., J.T.B., and X.A.L.; writing of the article: G.L., D.C.Z., J.T.B., and X.A.L.; review and editing: D.C.Z. and B.O.P.; funding acquisition: B.O.P.; supervision: D.C.Z. and B.O.P.

## Declaration of interests

The authors declare no competing interests.

## STAR★Methods

### Key resources table

**Table:** 

REAGENT or RESOURCE	SOURCE	IDENTIFIER
**Software and algorithms**		

Python	Python	https://www.python.org/
Analysis codes	This study	GitHub: https://github.com/SBRG/Bacterial_TRN_Dominance_and_Independence

### Method details

#### Regulator capture in PRECISE-1K

For calculation and analysis of regulon capture in PRECISE-1K iModulons, only strong and confirmed evidence sites from RegulonDB v14.5.0 were utilized for computation of categories of regulators and size of regulators which are associated with iModulons. A regulator is considered to be captured if it is listed as a regulator for at least one iModulon based on the enrichment protocol for regulators detailed in the PRECISE-1K paper.^38^ For not-captured regulators, a regulator was considered to be enriched if there was at least one iModulon which had a statistically significant enrichment value with an FDR of at most 1e−5. Precision and recall for each iModulon were calculated against the full PRECISE-1K TRN including all evidence levels. Components with multiple regulators operating together on the gene set (having an AND relationship) had precision and recall calculated against the intersection of all regulators’ genes, while regulators operating on different subsets of the gene set (having an OR relationship) had precision and recall calculated against the union of all regulators’ genes.

#### ArgR motif analysis

We utilized the Bitome framework for genetic data^57^ and the Position Specific Scoring Matrix from RegulonDB.^50^ We scanned the promoter region as annotated on Ecocyc^58^ in a region from +50 to −100 from the Transcription Start Site. The highest scoring log odds site was used.

### Quantification and statistical analysis

#### Benchmark of TRN inference methods

We used the PRECISE-1K^38^ dataset as the input for the fifteen methods compared in this study. For the network only methods, we used GENIE3^13^ (Direct Network Inference), WGCNA^12^ (Clustering), FLAME^39^ (Clustering), QUBIC^40^ (Biclustering), ISA^41^ (Biclustering). GENIE3 was performed using the GENIE3 package v1.26.0 in R v4.4.1 with default settings. We selected the top 250 regulators based on the total weights in the weight matrix. WGCNA was applied using the WGCNA package v1.73 in R v4.4.1. The blockwiseModules function was utilized with parameters set to minModuleSize = 3 and mergeCutHeight = 0.228 to get 250 co-expression modules. FLAME was carried out using the python implementation from https://github.com/yclicc/FLAME-python with cluster_neighbours = 3 and iteration_neighbours = 3 (the K parameter governing both the local-density/cluster-supporting-object step and the fuzzy-membership iteration), with all other parameters at the package defaults. QUBIC was executed in R v4.4.1 with rqubic v1.52.0 on the PRECISE-1K log-TPM matrix. Expression was discretized with quantileDiscretize() at the package defaults, seeded with generateSeeds() at the package defaults, and biclustered with quBicluster(report.no = 250) using the rqubic defaults for consistency (c = 0.95) and overlap filter (f = 1.0). ISA was executed in R v4.4.1 with isa2 v0.3.6 on the log-TPM matrix after column- and row-standardization with isa.normalize(). isa() was then called with default settings to generate the robust modules.

Next, we employed PCA,^42^ Incremental PCA,^44^ FastICA,^45^ NMF,^47^ Factor Analysis,^48^ Dictionary Learning,^49^ and SparseCoder^49^ using python v3.12.4 and scikit-learn^59^ v1.5.1. mixOmics^60^ v6.18.1 was utilized to run Independent PCA^43^ and sparse independent PCA^43^ on R v4.1.2. Sparse ICA^46^ was implemented using SparseICA^46^ v0.1.0 in R v4.4.1.

RegulonDB v14.5.0 was used as the reference to benchmark the performance of capturing regulons. For all the regulons in RegulonDB, we only select the genes that have strong/confirmed evidence to ensure the quality and reliability of the reference TRN. RegulonDB labels an interaction as strong evidence (S) when the regulation has one solid direct evidence, whereas confirmed (C) is assigned only when there are at least two independent types of Strong evidence supporting the regulation. Please see the RegulonDB page for the definition of the individual evidence types and how the confidence levels are assigned based on the addition of the individual evidences (https://regulondb.ccg.unam.mx/manual/help/evidenceclassification).

Next, we adopted the method from Sastry et al.^22^ to identify the key genes within each component. Briefly, in an iterative process, genes with the highest absolute values were removed, and the D'Agostino K^2^ test statistic^61^ (measuring skewness and kurtosis) was recalculated. Once the statistic dropped below a threshold, the removed genes were marked as significant. The threshold was determined by a sensitivity analysis by comparing the significant genes to the known regulons as described in Sastry et al.^22^ Next, we varied D'Agostino K^2^ statistics from 50 to 3000 at step 50, and simultaneously computed F1 score between components and regulons. The maximum average F1 score and corresponding D'Agostino K^2^ statistic was selected as the cutoff setting.

Each component was processed through the described threshold determination step and binarized based on the output cutoff. We quantified agreement between inferred gene modules and reference regulons using overlap-based classification metrics. Two binary gene-by-set matrices were used: a module membership matrix (genes × inferred modules) and a regulon membership matrix (genes × known regulators/regulons). Each entry indicated gene membership (1) or non-membership (0).

For each inferred module *m* and each regulon *r*, we treated gene membership as a binary labeling problem over genes *i* = 1, …,*G*. Let *y*_*i*_∈{0,1} denote membership of gene *i* in module *m*, and ${\hat{y}}_{i}\in \left\{0,1\right\}$ denote membership of gene *i* in regulon *r*. We computed confusion-matrix counts:

$$TP=\sum_{i=1}^{G}1\left(y_{i}=1\wedge {\hat{y}}_{i}=1\right)$$

$$FP=\sum_{i=1}^{G}1\left(y_{i}=0\wedge {\hat{y}}_{i}=1\right)$$

$$TN=\sum_{i=1}^{G}1\left(y_{i}=0\wedge {\hat{y}}_{i}=0\right)$$

$$FN=\sum_{i=1}^{G}1\left(y_{i}=1\wedge {\hat{y}}_{i}=0\right)$$

From these counts we computed precision (*P*), recall (*R*), F1 score (*F*_1_), and Matthews correlation coefficient (MCC) as:

$$P=\frac{TP}{TP+FP}$$

$$R=\frac{TP}{TP+FN}$$

$$F_{1}=\frac{2\;P\;R}{P+R}=\frac{2\;TP}{2\;TP+FP+FN}$$

$$\text{MCC}=\frac{TP\cdot TN-FP\cdot FN}{\sqrt{\left(TP+FP\right)\;\left(TP+FN\right)\;\left(TN+FP\right)\;\left(TN+FN\right)}}$$

To assign each inferred module a single best-matching regulon, we evaluated (*P*,*R*,*F*_1_,MCC) for that module against every regulon and selected the regulon with the maximum *F*_1_. The selected metrics for each module were then aggregated across all modules using a macro average (simple arithmetic mean across modules), yielding avg_F_1_, avg_precision, avg_recall, and avg_mcc. Because these are averages of per-module metrics (after per-module best-match selection), avg_F_1_ is not required to lie between avg_precision and avg_recall, even though for any single module-regulon pair the computed *F*_1_ lies between that pair’s *P* and *R*.

We ran the same process with dimensions 150, 200, 250, 300, and 350 on the PRECISE-1K dataset to show a robust benchmark across dimensions.

We also benchmarked the performance of these methods on a *Bacillus subtilis* expression data^62^ on 100, 150, and 200 components. The TRN reference was obtained from SubtiWiki.^63^ Average Precision, Average Recall, Average F1 score, and Average MCC were calculated with the same methodology as the PRECISE-1K steps.

#### Prior-based activity inference

Network Component Analysis^17^ was performed using the code provided in Lempp et al.^64^ The input network was downloaded from RegulonDB^50^ v14.5.0 (released January 2026; downloaded 2026-04-27) with the following manual curation. The decoupleR^20^’s python implementation (v1.8.0) of the Univariate Linear Model (ULM) was used to estimate TF activity. The same input of TRN was used for both the Network Component Analysis and decoupleR.

Components from the various TRN reconstruction methods were correlated with activities calculated by Network Component Analysis and decoupleR. Components to be correlated were selected by their enrichment of genes (determined by the above cutoff) in the network from RegulonDB. The most significant enrichment for a given component was considered the assigned regulator for comparison with activities from Network Component Analysis and decoupleR. For a regulator, the highest correlated component was kept to be compared to the activities computed by the activity-only methods.

#### Decision tree

To select the metrics for characterizing genes that are included in the iModulons, a decision tree was built using scikit-learn v1.0.2 and python 3.7. The decision tree performed 5-fold cross validation and pruned to max_depth = 2.

#### Independence and dominance levels for the regulons

The independence level of a regulon was calculated as follows: First, the pairwise mutual information (MI) between the regulon and every other regulon in the TRN was computed using the binary gene-membership indicator vectors of the two regulons over the full gene universe and the standard binary mutual information, computed using sklearn.metrics.mutual_info_score (scikit-learn v1.5.1).

$$MI\left(r_{i},r_{j}\right)=\sum_{a,b\in \left\{0,1\right\}}p_{ab}\left(r_{i},r_{j}\right)\;\ln\left(\frac{p_{ab}\left(r_{i},r_{j}\right)}{p_{a}\left(r_{i}\right)\;p_{b}\left(r_{j}\right)}\right)$$

These MI values were averaged to give the regulon’s overall MI with the other regulons

$$\bar{MI}\left(r_{i}\right)=\frac{1}{\left|R\right|-1}\sum_{\begin{array}{c} r_{j}\in R\\ j\neq i \end{array}}MI\left(r_{i},r_{j}\right)$$

To standardize the metric, the averaged MI was normalized by dividing it by the maximum averaged MI observed across all regulons, constraining the value to a range of 0–1. Since higher mutual information corresponds to stronger regulatory overlap (and thus lower independence), the normalized value was subtracted from 1 to invert the scale. The final independence level therefore increases with greater regulatory independence.

$$\text{Independence}\left(r_{i}\right)=1-\frac{\bar{MI}\left(r_{i}\right)}{\max_{r_{k}\in R}\;\bar{MI}\left(r_{k}\right)}$$

The dominance level of a regulon was calculated as follows. For each gene in the PRECISE-1K dataset, the explained variance contributed by its top-associated iModulon (the iModulon accounting for the highest variance in that gene’s expression) was calculated as the single-component coefficient of determination of the *X* = *MA* decomposition, where *X*_*g*,*s*_ is the log-TPM expression of gene *g* in sample *s*, *M*_*g*,*k*_ is the loading of gene *g* on iModulon *k*, *A*_*k*,*s*_ is the activity of iModulon *k* in sample *s*, and ${\bar{X}}_{g}$ is the mean expression of gene *g* across samples):

$$EV_{\text{top}}\left(g\right)=\max_{k=1,\ldots ,K}\;\left(1-\frac{\sum_{s}{\left(X_{g,s}-M_{g,k}\;A_{k,s}\right)}^{2}}{\sum_{s}{\left(X_{g,s}-{\bar{X}}_{g}\right)}^{2}}\right)$$

Within each regulon *r*_*i*_ with member-gene set *G*(*r*_*i*_), these top-iModulon explained variance values were averaged across all member genes

$${\bar{EV}}_{\text{top}}\left(r_{i}\right)=\frac{1}{\left|G\left(r_{i}\right)\right|}\sum_{g\in G\left(r_{i}\right)}EV_{\text{top}}\left(g\right)$$

The averaged value was then divided by the maximum averaged explained variance observed across all regulons, yielding a dominance level in the range [0,1]. Higher dominance levels indicate that genes in the regulon are predominantly regulated by a single iModulon:

$$\text{Dominance}\left(r_{i}\right)=\frac{{\bar{EV}}_{\text{top}}\left(r_{i}\right)}{\max_{r_{k}\in R}\;{\bar{EV}}_{\text{top}}\left(r_{k}\right)}$$

## Supplemental information

Document S1. Figures S1–S31 and Notes S1–S3

Table S1. Table of regulatory dominant genes in PRECISE-1K
