# Supplementary material for: Disentangling factors affecting bacterial transcriptional regulatory network inference
Source: iScience. 2026 Jul 16;29(8):116834. doi: 10.1016/j.isci.2026.116834 (PMC13401006; doi:10.1016/j.isci.2026.116834)
Supplement: Document S1. Figures S1–S31 and Notes S1–S3 [file mmc1.pdf]

**iScience, Volume *29***

## Supplemental information

# Disentangling factors affecting bacterial transcriptional regulatory network inference

**Gaoyuan Li, Joshua T. Burrows, Xuwen A. Lou, Bernhard O. Palsson, and Daniel C. Zielinski**

# Supplementary Note S1

## Rate Law Derivations for Different Transcriptional Regulation

To connect TRN inference algorithms to underlying transcriptional regulatory mechanisms, we introduce a chemical kinetic description of transcriptional regulation at a single promoter utilizing an established framework [S1]. In these models, we assume, as others have [S1, S2], that transcription is initiated in three primary steps. First, the RNA polymerase (RNAP) binds to the promoter, which can involve interactions with different promoter sites (e.g. -10, -35, upstream region) that can be modeled separately or not. Second, a transcriptional bubble is opened. Third, RNAP escapes the promoter to synthesize the RNA, restoring the promoter to its original state. Transcriptional regulation is then incorporated through binding of transcription factors to the promoter, which either reduces or increases effective RNAP binding. Finally, the dynamics of mRNA levels are captured through a mass balance between production via transcription at the promoter and consumption via degradation and growth dilution **(Supplementary Figure S1A and S1B)**.

Utilizing a chemical kinetic model of transcriptional regulation, the expression of a regulated gene relative to no regulation (which we term the "**mRNARatio**") can be derived. The resulting terms, one containing **promoter-specific** variables and one containing **condition-specific** variables **(Supplementary Figure S1C)**, can be seen to separate in an analogous fashion to the linear model assumed by many TRN inference methods, suggesting that TRN inference to an extent can be thought of as data-driven parameterization of a simple mechanistic promoter model. This relationship extends to several forms of the model to capture different common regulatory scenarios.

In this Michaelis-Menten-like model, promoter activity reaches a maximal rate at high RNAP and low inhibitor concentrations. From this equation, we applied two assumptions: first, that RNAP concentration is relatively saturating at its binding site, and second, that transcription factor regulation is relatively small. ICA is run on data that is normalized as log(TPM+1), and thus we try to derive a relationship on data normalized in the same manner. In this scenario, the **mRNARatio** can be shown to be equal to the multiplication of two terms, one of which is a condition-invariant promoter specific term, and the other is a condition-specific promoter-invariant term (See below). Strikingly, these terms correspond to the regulon structure and activity matrices in the **X = MA** model underlying many TRN inference methods. The condition-specific **A** matrix can capture nonlinearities despite an overall linear model structure of regulator contributions to expression of a single gene. Thus, there is potentially a strong basis to capture these effects within an **X = MA** structure in the case of a single inhibitor. Similar derivations are possible for regulation by transcriptional activators and sigma factors, but notably this decoupling is not possible if multiple regulators are considered simultaneously, suggesting that we would expect TRN inference to work significantly worse where significant regulator interactions occur (See below).

## Fundamental Principles

Mass balance equation

$$Mass\ Balance = v_{\text{production}} - v_{\text{consumption}} \qquad (1)$$

Production

$$v_{\text{production}} = v_{\text{transcription}} \tag{2}$$

Consumption

$$v_{\text{consumption}} = v_{\text{dilution}} + v_{\text{degradation}} = \mu \cdot mRNA + k_{\text{deg}} \cdot mRNA \tag{3}$$

Transcription rate

$$v_{\text{transcription}} = k_{\text{escape}} \cdot \sum(\text{open complexes}) \tag{4}$$

- $k_{\text{Escape}}$ - The rate constant for RNA polymerase escaping into active transcription.

For the scheme with both activator and inhibitor

$$v_{\text{transcription}} = k_{\text{escape}} \cdot \left(x_{\text{RNAP\_Open}} + x_{\text{RNAP\_Act\_Open}}\right) \tag{5}$$

## Derivations of the Transcription Rate Equation

Total promoter constraint

$$1 = x_{\text{RboundOpen}} + x_{\text{RboundClosed}} + x_{\text{TFboundClosed}} + x_{\text{Closed}} \tag{6}$$

- $x_{\text{RboundClosed}}$ - The promoter is bound to RNA polymerase but in a closed, non-transcribing state.
- $x_{\text{RboundOpen}}$ - The promoter is bound to RNA polymerase and has transitioned into an open (actively transcribing) state.
- $x_{\text{TFboundClosed}}$ - The promoter is bound to a transcription factor but remains in a closed state.
- $x_{\text{Closed}}$ - The promoter is in a closed state, unbound to any molecule (e.g., RNA polymerase or transcription factors).

Transcription rate

$$v_{\text{Transc}} = k_{\text{Escape}} \cdot x_{\text{RboundOpen}} \tag{7}$$

Equilibrium constant for promoter opening

$$K_{\text{eqOpening}} = \frac{x_{\text{RboundOpen}}}{x_{\text{RboundClosed}}} \tag{8}$$

$$x_{\text{RboundClosed}} = \frac{\text{RNAP} \cdot x_{\text{Closed}}}{K_{\text{D\_RNAP}}} \tag{9}$$

- $K_{\text{D\_RNAP}}$ - Dissociation constant for RNA polymerase binding

$$x_{\text{TFboundClosed}} = \frac{\text{TF} \cdot x_{\text{Closed}}}{K_{\text{D\_TF}}} \tag{10}$$

- $K_{\text{D\_TF}}$ - Dissociation constant for transcription factor binding

Solve (6) - (10) we have

$$x_{\text{RboundOpen}} = \frac{K_{\text{D\_TF}} K_{\text{eq\_Opening}} \text{RNAP}}{K_{\text{D\_RNAP}} K_{\text{D\_TF}} + K_{\text{D\_TF}} \text{RNAP} + K_{\text{D\_TF}} K_{\text{eq\_Opening}} \text{RNAP} + K_{\text{D\_RNAP}} \text{TF}} \tag{11}$$

$$x_{\text{RboundClosed}} = \frac{K_{\text{D\_RNAP}} K_{\text{D\_TF}} + K_{\text{D\_TF}} K_{\text{eq\_Opening}} \text{RNAP} + K_{\text{D\_RNAP}} \text{TF}}{K_{\text{D\_RNAP}} K_{\text{D\_TF}} + K_{\text{D\_TF}} \text{RNAP} + K_{\text{D\_TF}} K_{\text{eq\_Opening}} \text{RNAP} + K_{\text{D\_RNAP}} \text{TF}} \tag{12}$$

$$x_{\text{Closed}} = \frac{K_{\text{D\_RNAP}} K_{\text{D\_TF}}}{K_{\text{D\_RNAP}} K_{\text{D\_TF}} + K_{\text{D\_TF}} \text{RNAP} + K_{\text{D\_TF}} K_{\text{eq\_Opening}} \text{RNAP} + K_{\text{D\_RNAP}} \text{TF}} \tag{13}$$

$$x_{\text{TFboundClosed}} = \frac{K_{\text{D\_RNAP}} K_{\text{D\_TF}} \text{TF}}{K_{\text{D\_RNAP}} K_{\text{D\_TF}} + K_{\text{D\_TF}} \text{RNAP} + K_{\text{D\_TF}} K_{\text{eq\_Opening}} \text{RNAP} + K_{\text{D\_RNAP}} \text{TF}} \tag{14}$$

$$\begin{aligned} v_{\text{Transc}} &= k_{\text{Escape}} x_{\text{RboundOpen}} \\ &= \frac{K_{\text{D\_TF}} K_{\text{eq\_Opening}} k_{\text{Escape}} \text{RNAP}}{K_{\text{D\_RNAP}} K_{\text{D\_TF}} + K_{\text{D\_TF}} \text{RNAP} + K_{\text{D\_TF}} K_{\text{eq\_Opening}} \text{RNAP} + K_{\text{D\_RNAP}} \text{TF}} \end{aligned} \tag{15}$$

## Derivation of the mRNA ratio decomposition for single inhibitor

Mass Balance

$$\frac{dmRNA}{dt} = v_{\text{transc}} - mRNA\left(\mu + k_{\text{deg}}\right) \tag{16}$$

At steady state (SS)

$$\frac{dmRNA}{dt} = 0 \tag{17}$$

We have

$$mRNA = \frac{v_{\text{transc}}}{\mu + k_{\text{deg}}} \tag{18}$$

In this formulation, total mRNA, growth rate ($\mu$), and degradation rate ($k_{\text{deg}}$) are treated as constants. This is justified as $\mu$ is typically much smaller than $k_{\text{deg}}$, making growth rate variations negligible. Additionally, regulation of $k_{\text{deg}}$ is assumed to be a minor factor, which we acknowledge may not be the case. These factors collectively suggest their limited impact on the model.

SS solution without any regulation

$$mRNA_{\text{basal}} = mRNA(\text{Act} \to 0, \text{Inh} \to 0) \tag{19}$$

Relative expression

$$\begin{aligned} mRNARatio &= \frac{mRNA}{mRNA_{\text{basal}}} \\ &= \frac{K_{\text{D\_RNAP}} K_{\text{D\_TF}} + K_{\text{D\_TF}} \text{RNAP} + K_{\text{D\_TF}} K_{\text{eq\_Opening}} \text{RNAP}}{K_{\text{D\_RNAP}} K_{\text{D\_TF}} + K_{\text{D\_TF}} \text{RNAP} + K_{\text{D\_TF}} K_{\text{eq\_Opening}} \text{RNAP} + K_{\text{D\_RNAP}} \text{TF}} \end{aligned} \tag{20}$$

For a single inhibitor, we define

$$c_{\mathrm{Inhibitor}} = \frac{TF}{K_{\mathrm{D\_TF}}} \tag{21}$$

Simplify (20) to

$$mRNARatio = \frac{K_{\mathrm{D\_RNAP}} + \mathrm{RNAP} + K_{\mathrm{eq\_Opening}}\mathrm{RNAP}}{K_{\mathrm{D\_RNAP}} + \mathrm{RNAP} + K_{\mathrm{eq\_Opening}}\mathrm{RNAP} + c_{\mathrm{Inhibitor}}K_{\mathrm{D\_RNAP}}} \tag{22}$$

Inverse mRNA ratio

$$mRNARatioInv = \frac{c_{\mathrm{Inhibitor}}}{1 + \mathrm{RNAP}/K_{\mathrm{D\_RNAP}} + K_{\mathrm{eq\_Opening}} \cdot \mathrm{RNAP}/K_{\mathrm{D\_RNAP}}} + 1 \tag{23}$$

Take the logarithm of the equation and use $\log(1 + x) \approx x$ for small deviations from 1, assuming small regulation.

$$log(\mathrm{mRNARatioInv}) \approx mRNARatioInv - 1 \tag{24}$$

With (23) and (24) we have

$$\begin{aligned} \log(\mathrm{mRNARatioInv}) &= \log\left(\frac{c_{\mathrm{Inhibitor}}}{1 + \mathrm{RNAP}/K_{\mathrm{D\_RNAP}} + K_{\mathrm{eq\_Opening}} \cdot \mathrm{RNAP}/K_{\mathrm{D\_RNAP}}} + 1\right) \\ &= \frac{c_{\mathrm{Inhibitor}}}{1 + \mathrm{RNAP}/K_{\mathrm{D\_RNAP}} + K_{\mathrm{eq\_Opening}} \cdot \mathrm{RNAP}/K_{\mathrm{D\_RNAP}}} \end{aligned} \tag{25}$$

In matrix factorization formulation

$$log(\mathrm{mRNARatioInv}) = MA = \frac{c_{\mathrm{Inhibitor}}}{1 + \mathrm{RNAP}/K_{\mathrm{D\_RNAP}} + K_{\mathrm{eq\_Opening}} \cdot \mathrm{RNAP}/K_{\mathrm{D\_RNAP}}} \tag{26}$$

From (21) and (26)

$$\begin{aligned} MA &= TF\left(\frac{1/K_{\mathrm{D\_TF}}}{1 + \mathrm{RNAP}/K_{\mathrm{D\_RNAP}} + K_{\mathrm{eq\_Opening}} \cdot \mathrm{RNAP}/K_{\mathrm{D\_RNAP}}}\right) \\ &= \frac{TF}{\mathrm{RNAP}}\left(\frac{1/K_{\mathrm{D\_TF}}}{1/\mathrm{RNAP} + \left(1 + K_{\mathrm{eq\_Opening}}\right)/K_{\mathrm{D\_RNAP}}}\right) \end{aligned} \tag{27}$$

If $\frac{1}{\mathrm{RNAP}} \ll \frac{\left(1+K_{\mathrm{eq\_Opening}}\right)}{K_{\mathrm{D\_RNAP}}}$, which is when either $K_{\mathrm{eq\_Opening}}$ is favorable or RNAP is saturating, we can neglect the small term for simplification.

$$MA = \frac{TF}{\mathrm{RNAP}}\left(\frac{1/K_{\mathrm{D\_TF}}}{\left(1 + K_{\mathrm{eq\_Opening}}\right)/K_{\mathrm{D\_RNAP}}}\right) \tag{28}$$

This equation now separates into two distinct terms:

- One term related to the transcription factor activity (A)

- One term describing the intrinsic properties of transcription (M)

$$M = \frac{1/K_{\mathrm{D\_TF}}}{\left(1 + K_{\mathrm{eq\_Opening}}\right)/K_{\mathrm{D\_RNAP}}} \tag{29}$$

$$A = \frac{TF}{\mathrm{RNAP}} \tag{30}$$

## Derivation of the mRNA ratio decomposition for single activator

For a single activator

$$mRNARatio = \frac{\left(1 + c_{\mathrm{Activator}} K_{\mathrm{D\_RNAPAct}}\right)\left(K_{\mathrm{D\_RNAP}} + \mathrm{RNAP} + K_{\mathrm{eq\_Opening}}\mathrm{RNAP}\right)}{\left[(1 + c_{\mathrm{Activator}}) K_{\mathrm{D\_RNAP}} K_{\mathrm{D\_RNAPAct}} + c_{\mathrm{Activator}} K_{\mathrm{D\_RNAP}}\left(1 + K_{\mathrm{eq\_Opening}}\right)\mathrm{RNAP} + K_{\mathrm{D\_RNAPAct}}\left(1 + K_{\mathrm{eq\_Opening}}\mathrm{RNAP}\right))\right]} \tag{31}$$

$$\mathrm{mRNARatio} = \frac{\left(c_{\mathrm{Activator}} \frac{K_{\mathrm{D\_RNAP}}}{K_{\mathrm{D\_RNAPAct}}} + 1\right)\left(1 + \frac{\mathrm{RNAP}}{K_{\mathrm{D\_RNAP}}} + K_{\mathrm{eq\_Opening}} \frac{\mathrm{RNAP}}{K_{\mathrm{D\_RNAP}}}\right)}{\left((1 + c_{\mathrm{Activator}}) + c_{\mathrm{Activator}}\left(1 + K_{\mathrm{eq\_Opening}}\right) \frac{\mathrm{RNAP}}{K_{\mathrm{D\_RNAP}}} + \frac{1 + K_{\mathrm{eq\_Opening}}\mathrm{RNAP}}{K_{\mathrm{D\_RNAPAct}}}\right)} \tag{32}$$

Assume $\mathrm{RNAP}/K_{\mathrm{D\_RNAP}} \ll 1$

$$mRNARatioInv = \frac{1 + c_{\mathrm{Activator}}\left(1 + \frac{\mathrm{RNAP}}{K_{\mathrm{D\_RNAP\_Act}}}\left(1 + K_{\mathrm{eq\_Opening}}\right)\right)}{1 + c_{\mathrm{Activator}} K_{\mathrm{D\_RNAP}}/K_{\mathrm{D\_RNAP\_Act}}} \tag{33}$$

Multiply by $K_{\mathrm{D\_RNAP\_Act}}/K_{\mathrm{D\_RNAP}}$, which makes all of the terms 'small' when $c_{\mathrm{Activator}}$ is small

$$mRNARatioInv = \frac{\frac{K_{\mathrm{D\_RNAP\_Act}}}{K_{\mathrm{D\_RNAP}}} + c_{\mathrm{Activator}}\left(\frac{K_{\mathrm{D\_RNAP\_Act}}}{K_{\mathrm{D\_RNAP}}} + \frac{\mathrm{RNAP}}{K_{\mathrm{D\_RNAP}}}\left(1 + K_{\mathrm{eq\_Opening}}\right)\right)}{\frac{K_{\mathrm{D\_RNAP\_Act}}}{K_{\mathrm{D\_RNAP}}} + c_{\mathrm{Activator}}} \tag{34}$$

Assume $\mathrm{RNAP}$ is Saturating, $K_{\mathrm{D\_RNAP\_Act}}$ and $c_{\mathrm{Activator}}$ are small

$$mRNARatioInv = \frac{\frac{K_{\mathrm{D\_RNAP\_Act}}}{K_{\mathrm{D\_RNAP}}}}{\frac{K_{\mathrm{D\_RNAP\_Act}}}{K_{\mathrm{D\_RNAP}}} + c_{\mathrm{Activator}}} \tag{35}$$

$$mRNARatioInv = \frac{1}{1 + \frac{c_{\mathrm{Activator}} K_{\mathrm{D\_RNAP}}}{K_{\mathrm{D\_RNAP\_Act}}}} \tag{36}$$

$$mRNARatio = 1 + c_{\mathrm{Activator}} \frac{K_{\mathrm{D\_RNAP}}}{K_{\mathrm{D\_RNAP\_Act}}} \tag{37}$$

Now we have the (37), which is analogous to (23)

$$log(\mathrm{mRNARatio}) \approx mRNARatio - 1 \tag{38}$$

$$
\begin{aligned}
\log(\mathrm{mRNARatio}) &= \log\left(1 + c_{\mathrm{Activator}} \frac{K_{\mathrm{D\_RNAP}}}{K_{\mathrm{D\_RNAP\_Act}}}\right) \\
&= c_{\mathrm{Activator}} \frac{K_{\mathrm{D\_RNAP}}}{K_{\mathrm{D\_RNAP\_Act}}}
\end{aligned} \tag{39}
$$

By definition

$$
c_{\mathrm{Activator}} = \frac{TF}{K_{\mathrm{D\_TF}}} \tag{40}
$$

$$
log(\mathrm{mRNARatio}) = MA = TF \frac{K_{\mathrm{D\_RNAP}}}{K_{\mathrm{D\_RNAP\_Act}} K_{\mathrm{D\_TF}}} \tag{41}
$$

This equation now separates into two distinct terms:

- One term related to the transcription factor activity (A)

- One term describing the intrinsic properties of transcription (M)

## Derivation of the mRNA ratio decomposition for sigma factor

Total promoter constraint

$$
1 = x_{Closed} + x_{RboundClosed} + x_{RboundOpen} \tag{42}
$$

$$
\begin{aligned}
x_{\mathrm{RboundClosed}} &= \frac{\mathrm{RNAP} \cdot x_{\mathrm{Closed}}}{K_{\mathrm{D\_RNAP}}} \\
x_{\mathrm{RboundOpen}} &= x_{\mathrm{RboundClosed}} \cdot K_{\mathrm{eq\_Opening}}
\end{aligned} \tag{43, 44}
$$

Solve for Promoter States

$$
\begin{aligned}
x_{\mathrm{Closed}} &= \frac{K_{\mathrm{D\_RNAP}}}{K_{\mathrm{D\_RNAP}} + \mathrm{RNAP} + K_{\mathrm{eq\_Opening}} \cdot \mathrm{RNAP}} \\
x_{\mathrm{RboundClosed}} &= \frac{\mathrm{RNAP}}{K_{\mathrm{D\_RNAP}} + \mathrm{RNAP} + K_{\mathrm{eq\_Opening}} \cdot \mathrm{RNAP}} \\
x_{\mathrm{RboundOpen}} &= \frac{K_{\mathrm{eq\_Opening}} \cdot \mathrm{RNAP}}{K_{\mathrm{D\_RNAP}} + \mathrm{RNAP} + K_{\mathrm{eq\_Opening}} \cdot \mathrm{RNAP}}
\end{aligned} \tag{45,46,47}
$$

Transcription Rate

$$
v_{\mathrm{Transc}} = k_{\mathrm{Escape}} \cdot x_{\mathrm{RboundOpen}} + k_{\mathrm{EscapeBasal}} \cdot (x_{\mathrm{RboundClosed}} + x_{\mathrm{Closed}}) \tag{48}
$$

With (45) - (47)

$$v_{\mathrm{Transc}} = \frac{K_{\mathrm{eq\_Opening}} k_{\mathrm{Escape}} \mathrm{RNAP}/K_{\mathrm{D\_RNAP}}}{1 + \mathrm{RNAP}/K_{\mathrm{D\_RNAP}} + K_{\mathrm{eq\_Opening}} \mathrm{RNAP}/K_{\mathrm{D\_RNAP}}} + k_{\mathrm{EscapeBasal}} \left( \frac{1}{1 + \mathrm{RNAP}/K_{\mathrm{D\_RNAP}} + K_{\mathrm{eq\_Opening}} \mathrm{RNAP}/K_{\mathrm{D\_RNAP}}} + \frac{\mathrm{RNAP}/K_{\mathrm{D\_RNAP}}}{1 + \mathrm{RNAP}/K_{\mathrm{D\_RNAP}} + K_{\mathrm{eq\_Opening}} \mathrm{RNAP}/K_{\mathrm{D\_RNAP}}} \right) \tag{49}$$

Define

$$fRNAP = \frac{\mathrm{RNAP}}{K_{\mathrm{dRNAP}}} \tag{50}$$

From (49) and (50) we have

$$v_{\mathrm{Transc}} = \frac{K_{\mathrm{eq\_Opening}} k_{\mathrm{Escape}} \mathrm{fRNAP}}{1 + \mathrm{fRNAP} + K_{\mathrm{eq\_Opening}} \mathrm{fRNAP}} + k_{\mathrm{EscapeBasal}} \left( \frac{1}{1 + \mathrm{fRNAP} + K_{\mathrm{eq\_Opening}} \mathrm{fRNAP}} + \frac{\mathrm{fRNAP}}{1 + \mathrm{fRNAP} + K_{\mathrm{eq\_Opening}} \mathrm{fRNAP}} \right) \tag{51}$$

Relative Expression

$$mRNARatio = \frac{mRNA}{mRNA_{\mathrm{basal}}} \tag{52}$$

Steady State solution without any regulation

$$mRNA_{\mathrm{basal}} = mRNA(\mathrm{fRNAP} \to 0) \tag{53}$$

Relative mRNA Ratio

$$mRNARatio = \frac{\mathrm{fRNAP} K_{\mathrm{eq\_Opening}} k_{\mathrm{Escape}} + k_{\mathrm{EscapeBasal}} + \mathrm{fRNAP} k_{\mathrm{EscapeBasal}}}{\left(1 + \mathrm{fRNAP} + \mathrm{fRNAP} K_{\mathrm{eq\_Opening}}\right) k_{\mathrm{EscapeBasal}}} \tag{54}$$

$$mRNARatio = \frac{\mathrm{fRNAP} K_{\mathrm{eq\_Opening}} k_{\mathrm{Escape}}/k_{\mathrm{EscapeBasal}} + 1 + \mathrm{fRNAP}}{1 + \mathrm{fRNAP} + \mathrm{fRNAP} K_{\mathrm{eq\_Opening}}} \tag{55}$$

$$mRNARatio = 1 + \frac{\mathrm{fRNAP} K_{\mathrm{eq\_Opening}} k_{\mathrm{Escape}}/k_{\mathrm{EscapeBasal}} - \mathrm{fRNAP} K_{\mathrm{eq\_Opening}}}{1 + \mathrm{fRNAP} + \mathrm{fRNAP} K_{\mathrm{eq\_Opening}}} \tag{56}$$

Now we have the (56), which is analogous to (23) and (37)

$$mRNARatio = 1 \quad \text{when} \quad fRNAP \to 0 \tag{57}$$

## Equations of the mRNA ratio for one activator one inhibitor

$$mRNARatio = \frac{\left(c_{\mathrm{Activator}}K_{\mathrm{D\_RNAP}} + K_{\mathrm{D\_RNAP\_Act}}\right)\left(K_{\mathrm{D\_RNAP}} + \mathrm{RNAP} + K_{\mathrm{eq\_Opening}}\mathrm{RNAP}\right)}{\begin{array}{l}[(1 + c_{\mathrm{Activator}} + c_{\mathrm{Inhibitor}})]K_{\mathrm{D\_RNAP}}K_{\mathrm{D\_RNAP\_Act}} \\ +c_{\mathrm{Activator}}K_{\mathrm{D\_RNAP}}\left(1 + K_{\mathrm{eq\_Opening}}\right)\mathrm{RNAP} + K_{\mathrm{D\_RNAP\_Act}}\left(1 + K_{\mathrm{eq\_Opening}}\right)\mathrm{RNAP}\end{array}} \quad (58)$$

The decoupling is no longer possible when multiple regulators are simultaneously considered.

## Equations of the mRNA ratio for two inhibitors

$$mRNARatio = \frac{K_{\mathrm{D\_RNAP}} + \mathrm{RNAP} + K_{\mathrm{eq\_Opening}}\mathrm{RNAP}}{K_{\mathrm{D\_RNAP}} + c_{\mathrm{Inh1}}K_{\mathrm{D\_RNAP}} + c_{\mathrm{Inh2}}K_{\mathrm{D\_RNAP}} + \mathrm{RNAP} + K_{\mathrm{eq\_Opening}}\mathrm{RNAP}} \quad (59)$$

The decoupling is no longer possible when multiple regulators are simultaneously considered.

# Supplementary Notes S2

## Inference Method Primer

### GENIE3 [S3]

GENIE3 decomposes regulatory-network inference into one feature-importance regression problem per target gene: each gene's expression is predicted from the expression of all other genes by a Random Forest (or Extra-Trees) ensemble, and the variable-importance scores are read off as candidate edge weights $\hat{w}_{ij} = \mathrm{importance}(x_i;\ \mathrm{ensemble}_j)$. Input: an $S$-sample by $G$-gene expression matrix and, optionally, a list of candidate regulators. Output: a dense, directed, weighted $G \times G$ matrix that is thresholded post hoc into a ranked edge list.

### WGCNA [S4]

WGCNA builds a weighted gene co-expression network from a soft-thresholded adjacency

$$a_{ij} = |\,\mathrm{cor}(x_i, x_j)\,|^{\beta},$$

with $\beta$ chosen so the network approximates a scale-free topology, derives a topological-overlap dissimilarity from the adjacency, and partitions genes by average-linkage hierarchical clustering with a dynamic tree cut. Input: a gene by sample expression matrix. Output: a hard assignment of each gene to one of $K$ modules plus a per-module eigengene (the first PC of the module) as a sample-level summary of module activity.

### FLAME [S5]

FLAME identifies Cluster Supporting Objects (CSOs) as local-density maxima in the $k$-nearest-neighbour graph of genes, treats each CSO as the seed of one fuzzy cluster (plus a single outlier class), and iteratively updates every non-CSO gene's membership vector as the weighted average of its neighbours' memberships,

$$\mu_i^{(t+1)} = \sum_{j \in N(i)} w_{ij}\ \mu_j^{(t)},$$

until convergence. Input: a gene by sample expression matrix and the neighborhood size $k$. Output: a soft $G \times (K+1)$ fuzzy membership matrix, from which a hard partition is obtained by argmax.

### QUBIC [S6]

QUBIC first discretizes each gene's expression profile into a small signed-integer alphabet of qualitative levels (down-regulated, baseline, up-regulated) chosen from per-gene quantiles, then constructs a weighted gene-pair graph whose edges score the consistency of qualitative co-behaviour across conditions, and finally identifies biclusters by seeded greedy expansion that adds rows or columns while maintaining a preset consistency threshold (default $c = 0.95$). Input: a gene by sample expression matrix. Output: a list of overlapping gene by condition biclusters, each sharing a discretised pattern.

**ISA [S7]**

The Iterative Signature Algorithm finds self-consistent gene-condition modules by alternating two thresholded projections through the row- and column-standardized expression matrix $E$: starting from a gene signature $g^{(t)}$, the condition signature is

$$c^{(t)} = T_{\theta_c}\big(E^\top g^{(t)}\big), \qquad g^{(t+1)} = T_{\theta_g}\big(E\, c^{(t)}\big),$$

where $T_\theta(\cdot)$ retains entries whose $z$-score exceeds $\theta$. Input: a standardised gene by sample expression matrix and the two thresholds $(\theta_g, \theta_c)$. Output: a library of overlapping signature biclusters, each a gene set paired with the conditions on which it is coherently expressed.

**PCA [S8]**

Principal Component Analysis finds the orthonormal directions in gene space along which the sample-by-sample variance is maximized, equivalently the rank-$K$ truncated singular value decomposition

$$X \approx U\Sigma V^\top, \quad \min_{U,\Sigma,V} \parallel X - U\Sigma V^\top \parallel_F.$$

Input: a centered (and usually variance-scaled) gene by sample matrix. Output: a gene by $K$ loading matrix $U$ (components, ordered by explained variance) and a $K$ by sample score matrix $\Sigma V^\top$ (activities).

**Incremental PCA [S9]**

Incremental PCA solves the same rank-$K$ approximation as PCA but streams the data: it maintains a running eigen-basis and updates it as each new mini-batch of samples arrives, so the full sample matrix never has to be held in memory at once. In the limit of a single batch the algorithm is equivalent to batch PCA. Output: the same gene by $K$ loading matrix and $K$ by sample score matrix as PCA, with peak memory bounded by the batch size rather than by the full matrix.

**Independent PCA (IPCA) [S10]**

IPCA runs PCA first to obtain orthogonal loading vectors and then applies an ICA rotation to those loadings, so the recovered gene loadings are not merely uncorrelated but also as statistically independent as possible across genes ("ICA used as a denoising of the PCA loading vectors"). Input: a centred gene by sample matrix and a chosen $K$. Output: a gene by $K$ loading matrix whose columns are independent in the ICA sense, plus a $K$ by sample activity matrix; the implementation in the benchmark is the ipca function of mixOmics.

**Sparse Independent PCA (sIPCA) [S10]**

sIPCA augments IPCA with an $L_1$ (LASSO-type) penalty on the loading vectors, so the post-PCA ICA rotation is computed on simultaneously shrunken loadings and an internal gene-level variable selection is performed during the fit. Input: a centred gene by sample matrix, $K$, and a sparsity hyperparameter $\lambda$. Output: a sparse gene by $K$ loading matrix in which many entries are exactly zero, plus a dense $K$ by sample activity matrix; sparsity is enforced on the gene side only.

**FastICA [S11]**

FastICA seeks statistically independent sources by maximizing a non-Gaussianity proxy, the negentropy approximation

$$J(y) \;\approx\; (\,\mathbb{E}\{G(y)\} - \mathbb{E}\{G(\nu)\}\,)^2,$$

for a non-quadratic contrast $G$ (typically $G(u) = \log\cosh u$) with Gaussian reference $\nu$, under a whitening constraint that decorrelates the sources. The one-unit fixed-point update is

$$w \;\leftarrow\; \mathbb{E}\{x\, g(w^\top x)\} \;-\; \mathbb{E}\{g'(w^\top x)\}\, w, \qquad w \leftarrow w / \| w \|,$$

where $g = G'$; the algorithm returns a gene by $K$ mixing matrix $M$ and a $K$ by sample activity matrix $A$ with $X \approx MA$. This is the central matrix-factorization method evaluated in the present paper.

**Sparse ICA [S12]**

Sparse ICA combines an ICA negentropy or log-likelihood objective with an $L_1$ penalty on the source matrix,

$$\mathcal{L}_{\mathrm{sICA}}(M, A) \;=\; -\mathrm{loglik}_{\mathrm{ICA}}(M, A;\, X) \;+\; \lambda \, \| M \|_1,$$

and solves the joint non-smooth problem with a relax-and-split scheme that alternates a smooth ICA update with a soft-thresholding (proximal) step on $M$. Input: a centred gene by sample matrix, $K$, and the sparsity weight $\lambda$ (selected by a BIC-like criterion). Output: a sparse gene by $K$ component matrix in which many loadings are exactly zero, plus the corresponding $K$ by sample activity matrix.

**NMF [S13]**

Non-negative Matrix Factorization minimizes

$$\min_{M \geq 0,\, A \geq 0} \; \| X - MA \|_F^2,$$

(or a Kullback-Leibler divergence variant) so both gene loadings and sample activities are interpretable as additive parts rather than as signed contrasts. The Lee and Seung multiplicative updates are

$$M \leftarrow M \odot \frac{XA^\top}{MAA^\top}, \qquad A \leftarrow A \odot \frac{M^\top X}{M^\top MA},$$

with element-wise multiplication and division. Input: a non-negative gene by sample matrix (counts, TPMs, or shifted log-TPMs) and a chosen rank $K$.

**Factor Analysis [S14]**

Classical Gaussian Factor Analysis models each gene's expression as a linear combination of $K$ latent factors plus an independent per-gene Gaussian noise term,

$$X = MA + \varepsilon, \qquad \varepsilon \sim \mathcal{N}\big(0,\, \mathrm{diag}(\psi_1, \ldots, \psi_G)\big),$$

and fits the gene by $K$ loading matrix $M$, the $K$ by sample latent activities $A$, and the per-gene "uniquenesses" $\psi_g$ by maximum likelihood (typically EM). Input: a centred gene by sample matrix and a chosen $K$. Output: a gene by $K$ loading matrix, a $K$ by sample latent-factor activity matrix, and a vector of per-gene noise variances.

**Dictionary Learning [S15]**

Dictionary Learning jointly learns a (typically over-complete) gene by $K$ dictionary $D$ and a $K$ by sample sparse code $A$ by minimising

$$\min_{D,A} \parallel X - DA \parallel_F^2 + \lambda \parallel A \parallel_1,$$

alternating a sparse-coding step (find $A$ given $D$, e.g. by LASSO) with a dictionary-update step (refit $D$ given $A$). Input: a centred gene by sample matrix, the dictionary size $K$, and a sparsity weight $\lambda$. Output: a dense gene by $K$ dictionary whose atoms read as gene programmes, plus a sparse $K$ by sample code in which each sample uses only a few atoms (sparsity is enforced on the sample side).

**Sparse Coding (SparseCoder) [S15]**

SparseCoder is the inference-only counterpart of Dictionary Learning: given a *fixed* gene by $K$ dictionary $D$ (here pre-learned by Dictionary Learning on the same matrix), it solves one sparse regression problem per sample,

$$\min_{a_s} \parallel x_s - Da_s \parallel_2^2 + \lambda \parallel a_s \parallel_1,$$

to obtain the sparse code that best reconstructs that sample under an $L_1$ (LASSO) or $L_0$ (OMP) penalty. The dictionary is not modified during inference. Output: the $K$ by sample sparse activity matrix.

**NCA [S16]**

Network Component Analysis solves the bilinear factorization $X \approx MA$ under the constraint that the gene by TF mixing matrix $M$ has the same zero-pattern as a user-supplied binary connectivity matrix $Z$ ($M$ is non-zero only where $Z$ says a TF can regulate a gene), with $Z$ required to satisfy the NCA identifiability criteria,

$$\min_{M,A} \parallel X - MA \parallel_F^2 \qquad \text{s.t.} \quad M_{gt} = 0 \;\; \text{whenever} \;\; Z_{gt} = 0.$$

$M$ and the TF by sample activity matrix $A$ are estimated by alternating least squares. Input: an expression matrix $X$ and a binary connectivity matrix $Z$ (here RegulonDB v14.5 C+S); output: a TF by sample activity matrix $A$ and a refined gene by TF connectivity-strength matrix $M$.

**decoupleR ULM [S17]**

decoupleR's Univariate Linear Model (ULM) fits, for each sample $s$ and each TF $t$, a univariate ordinary least-squares regression of the sample's gene-expression vector $x_s$ on the TF's signed target-indicator vector $r_t$ (with $+1$ for activating targets, $-1$ for repressing targets, $0$ for non-targets),

$$x_s = \alpha + \beta\, r_t + \varepsilon, \qquad \text{activity}_{t,s} = \frac{\hat{\beta}}{\widehat{\text{SE}}(\hat{\beta})},$$

and reports the $t$-statistic of the slope as the activity of TF $t$ in sample $s$. Input: a gene by sample expression matrix and a signed TF-to-gene reference network as an edge list (here RegulonDB v14.5.0 C+S). Output: a TF by sample activity matrix whose entries are signed $t$-statistics with a built-in statistical scale.

# Supplementary Note S3

### Regulator capture by ICA

Precision and recall of components were computed for all components computed by FastICA on the dataset for the assigned regulators. 86.7% of components had a precision of greater than or equal to 0.5, while only 56.6% of components had a recall of 0.5 or greater **(Supplementary Figure S22A)**. The Spearman correlation between regulon size and recall was -0.864 (low recall is consistent with the role of the dominant subset of genes) while the correlation between regulon size and precision was only 0.173, as large regulators were often associated with a component which captured a high-precision subset of the regulator while smaller regulators or cases of multiple-regulation were responsible for almost all cases of high recall values.

ICA often decomposes areas of complex multiple regulation or hierarchical regulation into individual components separate from related single-regulator components. The Ribose iModulon captures an operon regulated by dsrA, RbsR, and Crp, all three of which also regulate genes outside of this regulon. This iModulon captures the genes present in this operon only and none regulated by any of the individual regulators, indicating that there is complex regulation occurring from all regulators acting on this locus **(Supplementary Figure S22B)**. Herein, ICA fails to capture as a signal how each of these regulators influences the genes in this locus as a series of components and instead captures their regulation as a single component. These genes are therefore captured in an iModulon which describes their expression, but it does not display an accurate model of dominant regulation modes at this locus. The FucR/ExuR iModulon captures one operon regulated by FucR and five genes regulated by ExuR. These regulators do not have co-regulation of any of these genes and respond to different metabolites **(Supplementary Figure S22C)**. However, due to lack of differentiation in activation conditions across the dataset, the hierarchical regulation of both regulators and their associated genes via CRP results in both regulators being captured in this component.

### iModulon-only Genes in the Arginine iModulon

The Arginine iModulon includes four genes that are not part of the ArgR regulon. While these genes exhibit strong correlation with the other genes in the Arginine iModulon, they are not officially classified within the regulon **(Figure 5B)**. For instance, *asnA* and *dtpD* are positioned near two promoters, and show high motif log-odds scores for ArgR binding sites upstream of these promoters. The expression of *dtpD* is correlated with Arginine iModulon activity **(Supplementary Figure S27A and S27D)**. Meanwhile, *caiF* and *xynR* are located near genes or operons regulated by ArgR, as *xynR* expression is highly correlated with *argF* expression, suggesting the possibility of co-transcription **(Supplementary Figure S27B and S27C)**. This suggests that they might also be part of the ArgR regulon.

### Dominance of the NtrC on ast Operon

The green dot represents a low nitrogen condition **(Supplementary Figure S29A and S29B)**, where only NtrC is activated, resulting in high expression of *astC*. The orange dot represents a cytidine-supplemented condition, where both NtrC and ArgR are activated, leading to a similarly high level of *astC* expression, which shows that the activation of ast operon is dominantly controlled by NtrC **(Supplementary Figure S29A and S29B)**.

# Supplementary Figures

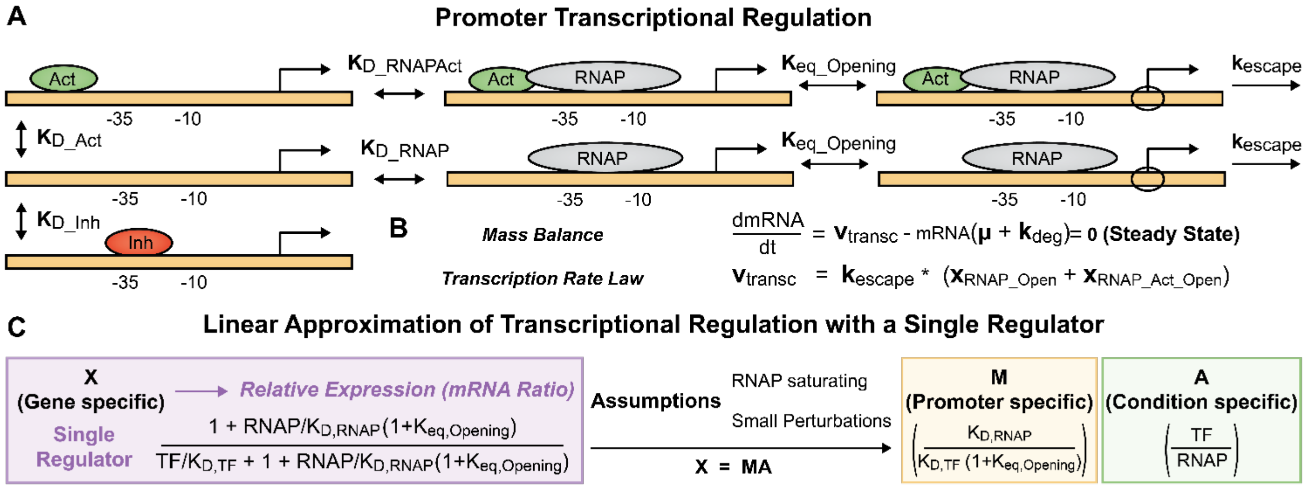


**Figure S1: Kinetic description and equations of transcriptional regulation.**

**A)** Simple biophysical kinetic mechanism for transcription and its regulation by an inhibitor or an activator (single strong regulator). $K_{D\_Act}$ is the dissociation constant for activator binding; $K_{D\_Inh}$ is the dissociation constant for inhibitor binding; $K_{D\_RNAPAct}$ is the dissociation constant for activator-RNAP binding; $K_{D\_RNAP}$ is the dissociation constant for RNAP binding; $Keq_{Opening}$ is the equilibrium constant for promoter opening. $k_{escape}$ is the rate constant for RNA polymerase escaping into active transcription. **B)** Resulting mass balance and transcription rate law derived from the transcription mechanism described in panel A. $\mu$ is the growth rate and $k_{deg}$ is the degradation rate, and they are treated as constant in this formulation. $V_{transc}$ is the transcription rate. $X_{RNAP\_Open}$ and $X_{RNAP\_Act\_Open}$ are the proportions of promoters that are bound to RNAP or Activator-RNAP and have transitioned into an open, actively transcribing state. **C)** An equation for the relative expression between a treated and a control condition factored into a promoter-specific term and condition-specific term after applying several assumptions. The resulting relationship forms a mechanistic basis for the X = MA linear model underlying the TRN inference from gene expression data.

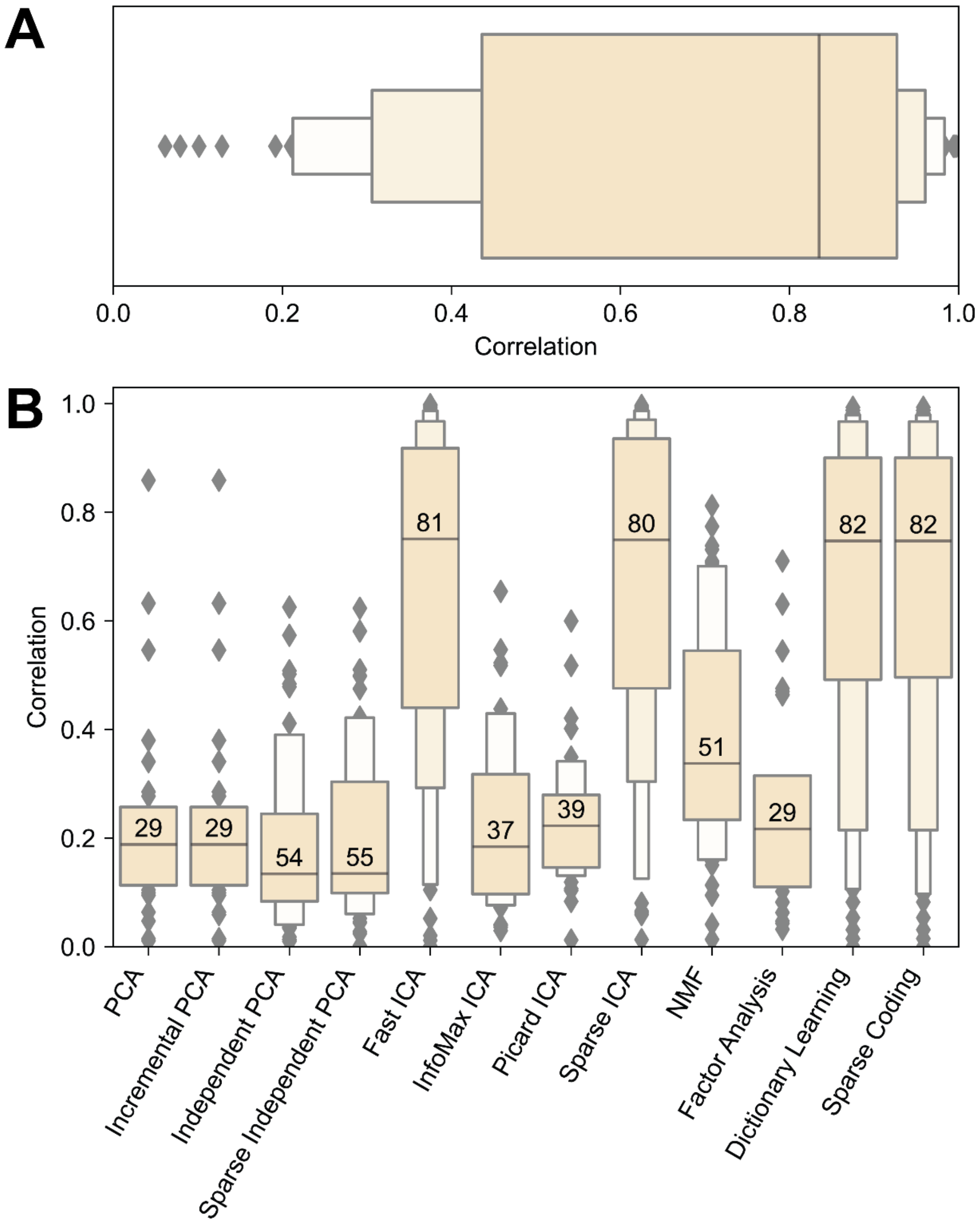


**Figure S2. Consistency between the Activity-only inference methods.**

**A)** Boxenplot (enhanced box plot with more detailed distribution visualization in the tails) of the Correlations between the activities inferred from two Activity-only methods, decoupleR ULM and NCA. **B)** Benchmark for inferred activities. Network-and-Activity methods are evaluated by measuring the correlation between the inferred activities from these methods and those from Activity-only methods. Each network-and-activity method has the number of regulators (n) enriched for the method and the correlation of the corresponding components plotted. Results from Network Component Analysis (NCA) are presented here.

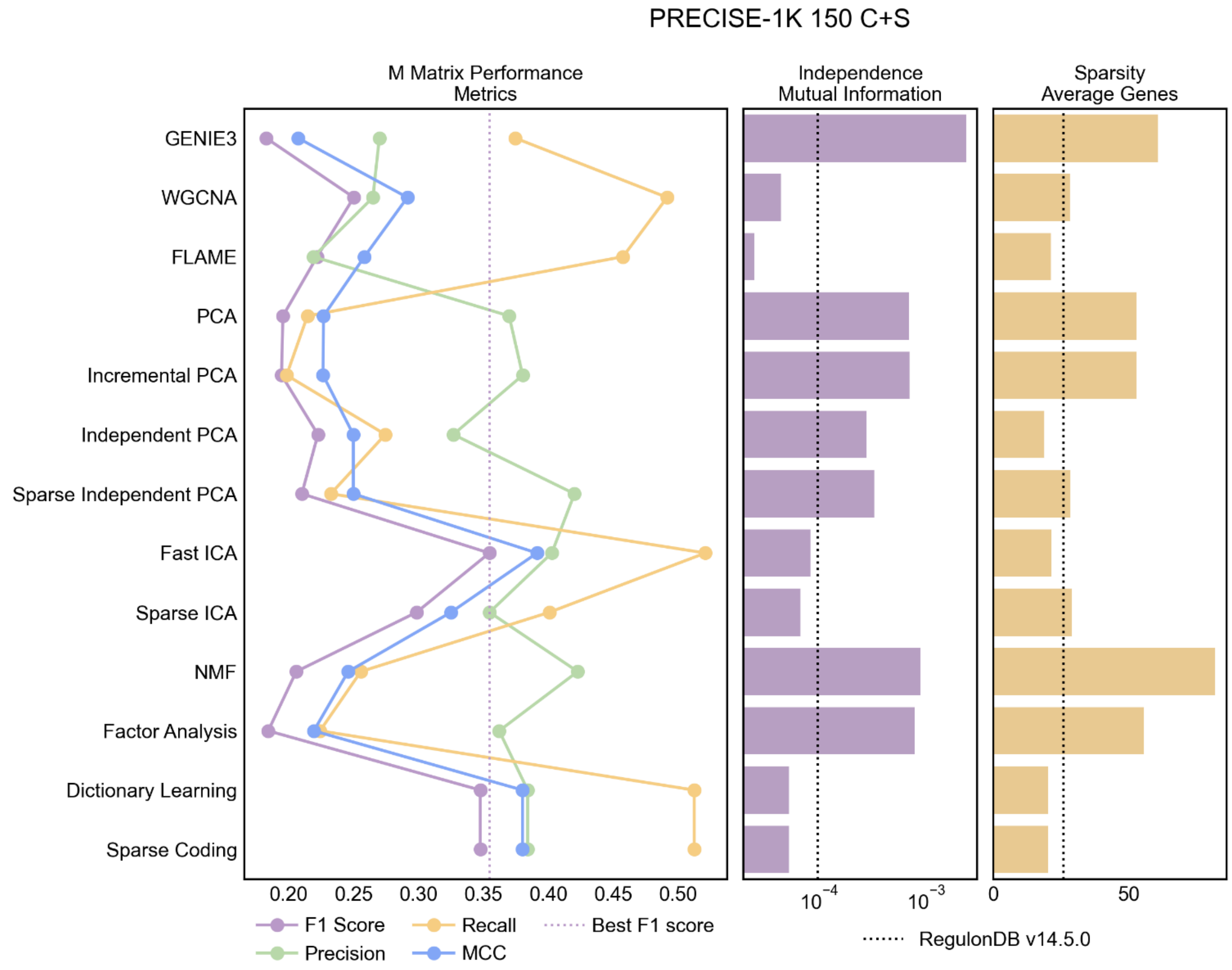


**Figure S3. Benchmark for the inference methods on PRECISE-1K (150 Components)**

Benchmark for the inference methods on PRECISE-1K (150 Components). Average Precision, Average Recall, Average F1 score, and Average Matthews Correlation Coefficient (MCC) were used for the M matrix benchmark. For the Network-only methods, we evaluated GENIE3 (direct network inference), WGCNA (clustering) and QUBIC (biclustering). A total of ten matrix factorization methods were examined covering Principal Component Analysis (PCA), Independent PCA, Sparse Independent PCA, Incremental PCA, FastICA, Sparse ICA, Non-negative matrix factorization (NMF), Factor Analysis, Dictionary Learning, and Sparse Coding. The M matrices obtained from these methods were binarized following the approach described by Sastry et al. The methods are evaluated based on their ability to accurately capture regulons from RegulonDB v14.5.0 Confirmed and Strong (C+S) interactions (see Methods section for details). The average mutual information and sparsity of the M matrices from various TRN inference methods were calculated. The values from RegulonDB TRN were plotted on the vertical dashed lines as a reference.

Note: FLAME and ISA were excluded from the benchmarks in Figures S3–S15 because they do not allow the user to specify the number of components; obtaining an exact component count would require manual parameter tuning for each run, which is not feasible across all dimensions.

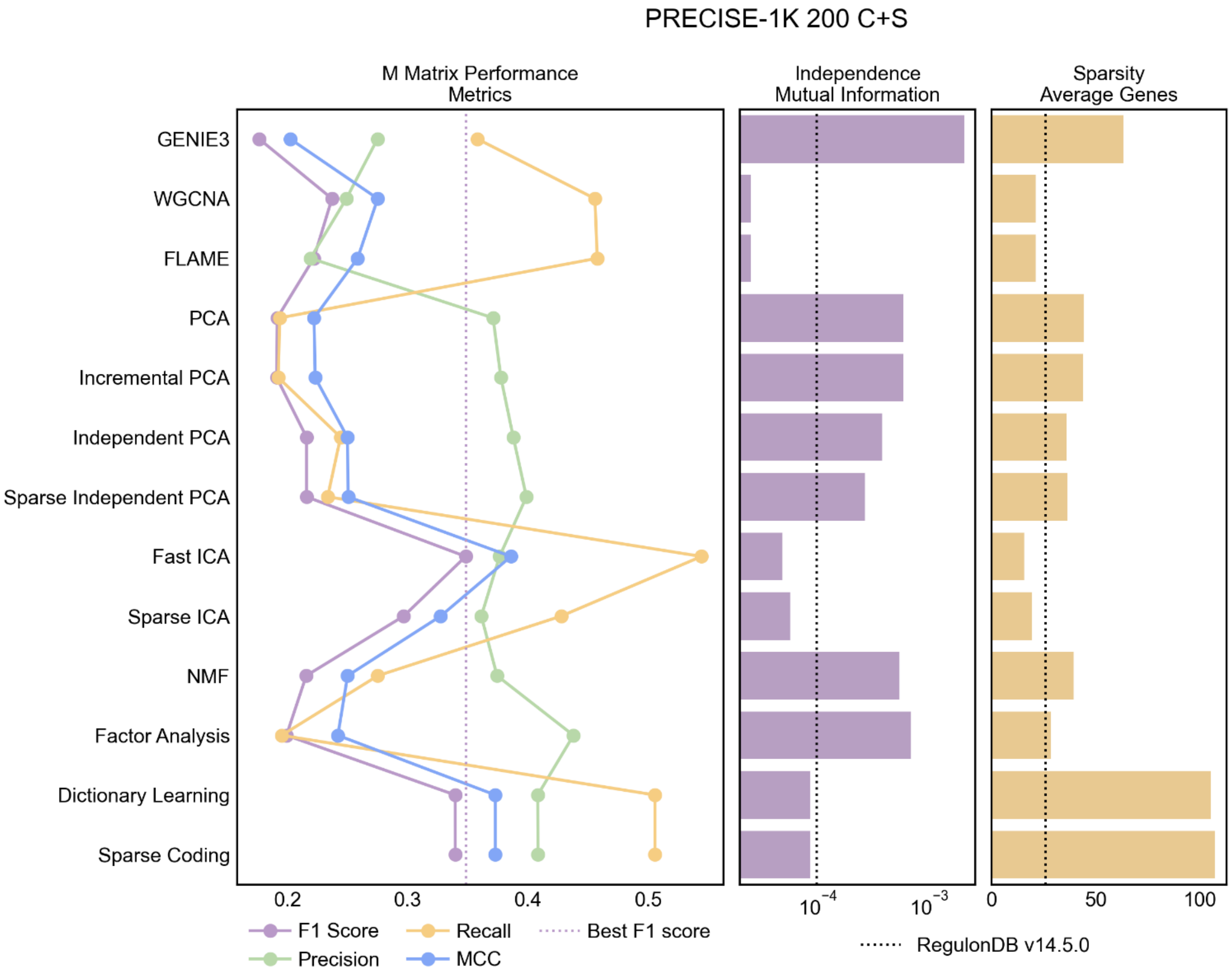


**Figure S4. Benchmark for the inference methods on PRECISE-1K (200 Components)**

Benchmark for the inference methods on PRECISE-1K (200 Components). Average Precision, Average Recall, Average F1 score, and Average Matthews Correlation Coefficient (MCC) were used for the M matrix benchmark. For the Network-only methods, we evaluated GENIE3 (direct network inference), WGCNA (clustering) and QUBIC (biclustering). A total of ten matrix factorization methods were examined covering Principal Component Analysis (PCA), Independent PCA, Sparse Independent PCA, Incremental PCA, FastICA, Sparse ICA, Non-negative matrix factorization (NMF), Factor Analysis, Dictionary Learning, and Sparse Coding. The M matrices obtained from these methods were binarized following the approach described by Sastry et al. The methods are evaluated based on their ability to accurately capture regulons from RegulonDB v14.5.0 Confirmed and Strong (C+S) interactions (see Methods section for details). The average mutual information and sparsity of the M matrices from various TRN inference methods were calculated. The values from RegulonDB TRN were plotted on the vertical dashed lines as a reference.

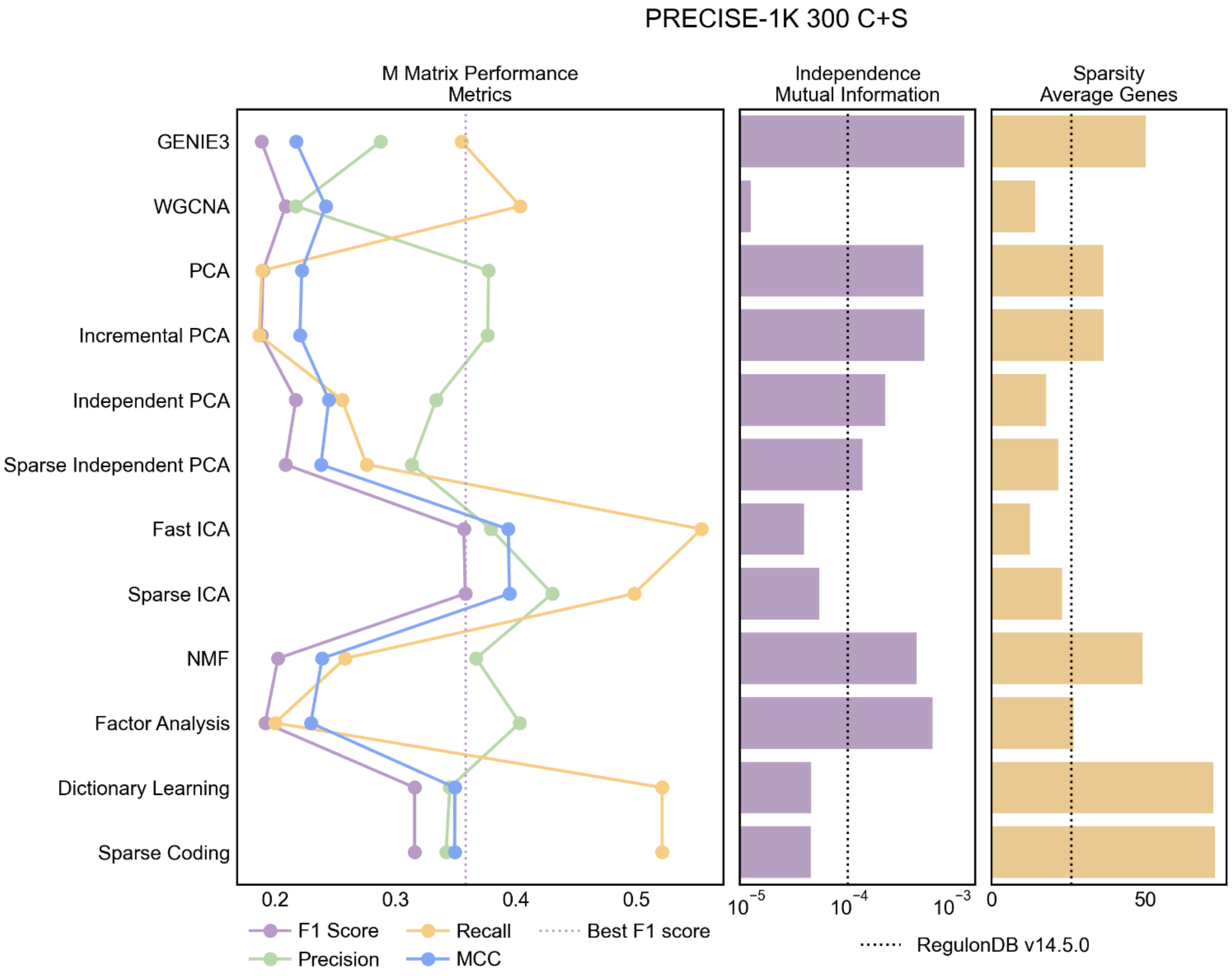


**Figure S5. Benchmark for the inference methods on PRECISE-1K (300 Components)**

Benchmark for the inference methods on PRECISE-1K (300 Components). Average Precision, Average Recall, Average F1 score, and Average Matthews Correlation Coefficient (MCC) were used for the M matrix benchmark. For the Network-only methods, we evaluated GENIE3 (direct network inference), WGCNA (clustering) and QUBIC (biclustering). A total of ten matrix factorization methods were examined covering Principal Component Analysis (PCA), Independent PCA, Sparse Independent PCA, Incremental PCA, FastICA, Sparse ICA, Non-negative matrix factorization (NMF), Factor Analysis, Dictionary Learning, and Sparse Coding. The M matrices obtained from these methods were binarized following the approach described by Sastry et al. The methods are evaluated based on their ability to accurately capture regulons from RegulonDB v14.5.0 Confirmed and Strong (C+S) interactions (see Methods section for details). The average mutual information and sparsity of the M matrices from various TRN inference methods were calculated. The values from RegulonDB TRN were plotted on the vertical dashed lines as a reference.

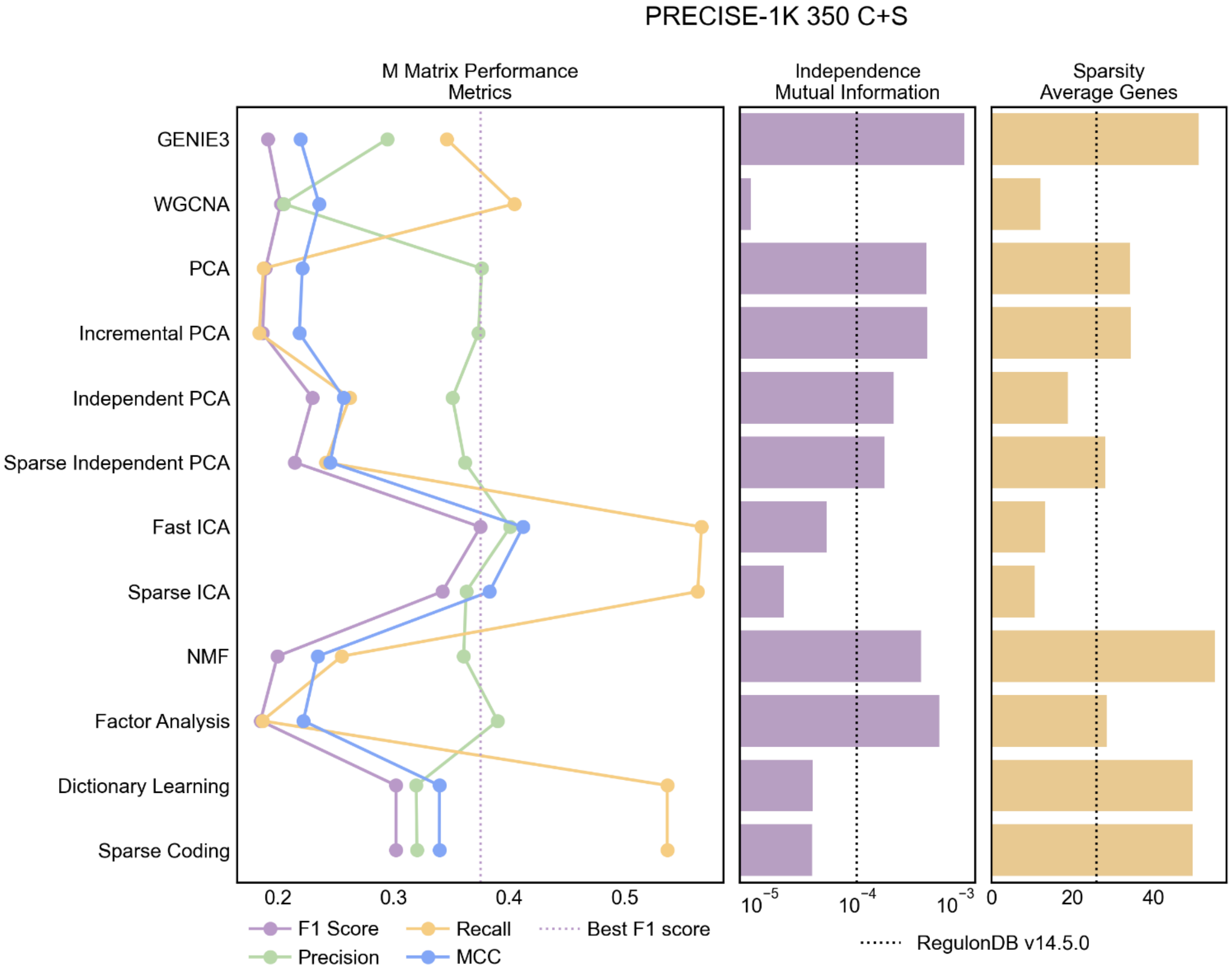


**Figure S6. Benchmark for the inference methods on PRECISE-1K (350 Components)**

Benchmark for the inference methods on PRECISE-1K (350 Components). Average Precision, Average Recall, Average F1 score, and Average Matthews Correlation Coefficient (MCC) were used for the M matrix benchmark. For the Network-only methods, we evaluated GENIE3 (direct network inference), WGCNA (clustering) and QUBIC (biclustering). A total of ten matrix factorization methods were examined covering Principal Component Analysis (PCA), Independent PCA, Sparse Independent PCA, Incremental PCA, FastICA, Sparse ICA, Non-negative matrix factorization (NMF), Factor Analysis, Dictionary Learning, and Sparse Coding. The M matrices obtained from these methods were binarized following the approach described by Sastry et al. The methods are evaluated based on their ability to accurately capture regulons from RegulonDB v14.5.0 Confirmed and Strong (C+S) interactions (see Methods section for details). The average mutual information and sparsity of the M matrices from various TRN inference methods were calculated. The values from RegulonDB TRN were plotted on the vertical dashed lines as a reference.

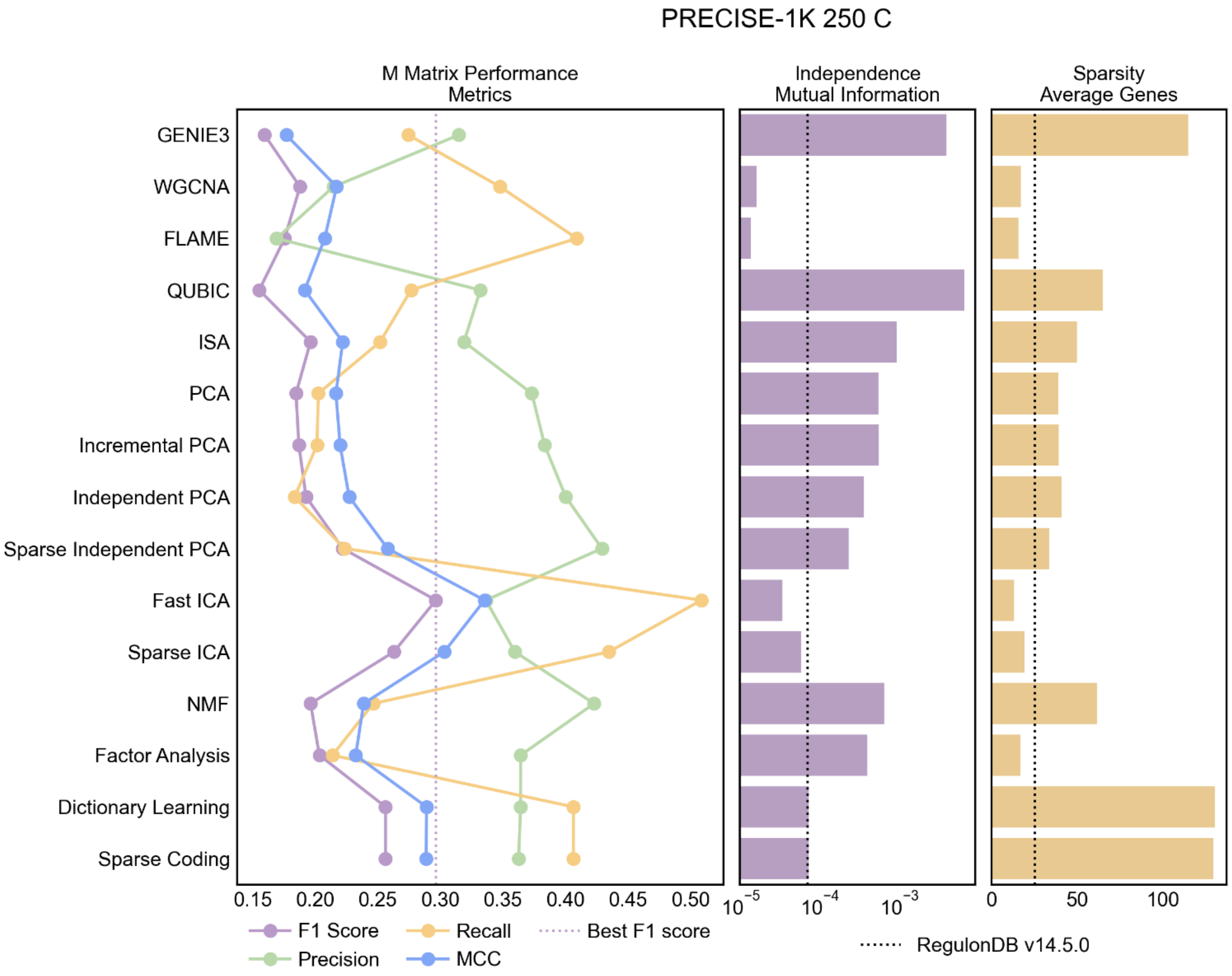


**Figure S7. Benchmark for the inference methods on PRECISE-1K (250 Components, Confirmed)**

Benchmark for the inference methods on PRECISE-1K (250 Components, Confirmed). Average Precision, Average Recall, Average F1 score, and Average Matthews Correlation Coefficient (MCC) were used for the M matrix benchmark. For the Network-only methods, we evaluated GENIE3 (direct network inference), WGCNA (clustering) and QUBIC (biclustering). A total of ten matrix factorization methods were examined covering Principal Component Analysis (PCA), Independent PCA, Sparse Independent PCA, Incremental PCA, FastICA, Sparse ICA, Non-negative matrix factorization (NMF), Factor Analysis, Dictionary Learning, and Sparse Coding. The M matrices obtained from these methods were binarized following the approach described by Sastry et al. The methods are evaluated based on their ability to accurately capture regulons from RegulonDB v14.5.0 Confirmed (C) interactions (see Methods section for details). The average mutual information and sparsity of the M matrices from various TRN inference methods were calculated. The values from RegulonDB TRN were plotted on the vertical dashed lines as a reference.

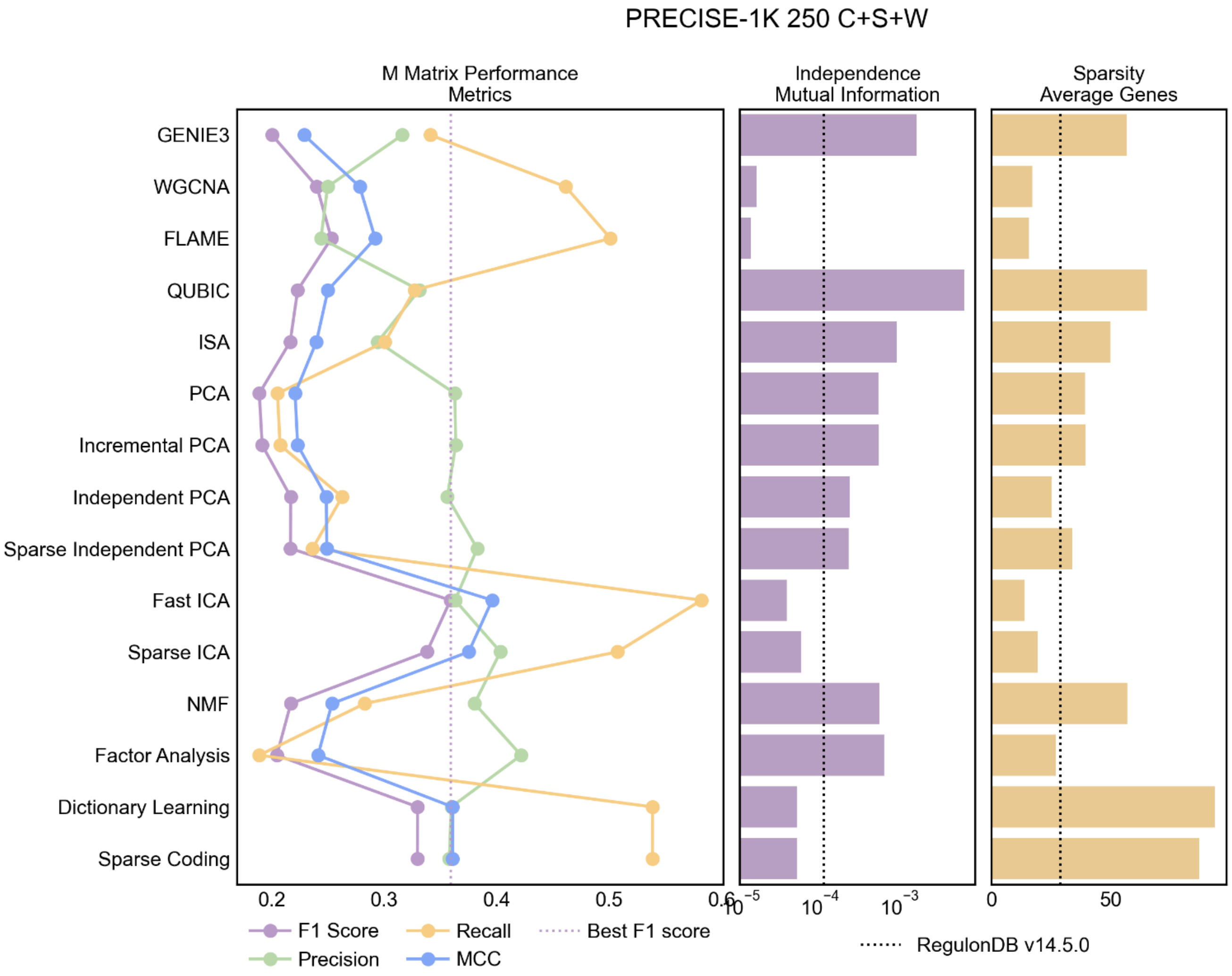


**Figure S8. Benchmark for the inference methods on PRECISE-1K (250 Components, Confirmed+Strong+Weak)**

Benchmark for the inference methods on PRECISE-1K (250 Components, Confirmed+Strong+Weak). Average Precision, Average Recall, Average F1 score, and Average Matthews Correlation Coefficient (MCC) were used for the M matrix benchmark. For the Network-only methods, we evaluated GENIE3 (direct network inference), WGCNA (clustering) and QUBIC (biclustering). A total of ten matrix factorization methods were examined covering Principal Component Analysis (PCA), Independent PCA, Sparse Independent PCA, Incremental PCA, FastICA, Sparse ICA, Non-negative matrix factorization (NMF), Factor Analysis, Dictionary Learning, and Sparse Coding. The M matrices obtained from these methods were binarized following the approach described by Sastry et al. The methods are evaluated based on their ability to accurately capture regulons from RegulonDB v14.5.0 Confirmed+Strong+Weak (C+S+W) interactions (see Methods section for details). The average mutual information and sparsity of the M matrices from various TRN inference methods were calculated. The values from RegulonDB TRN were plotted on the vertical dashed lines as a reference.

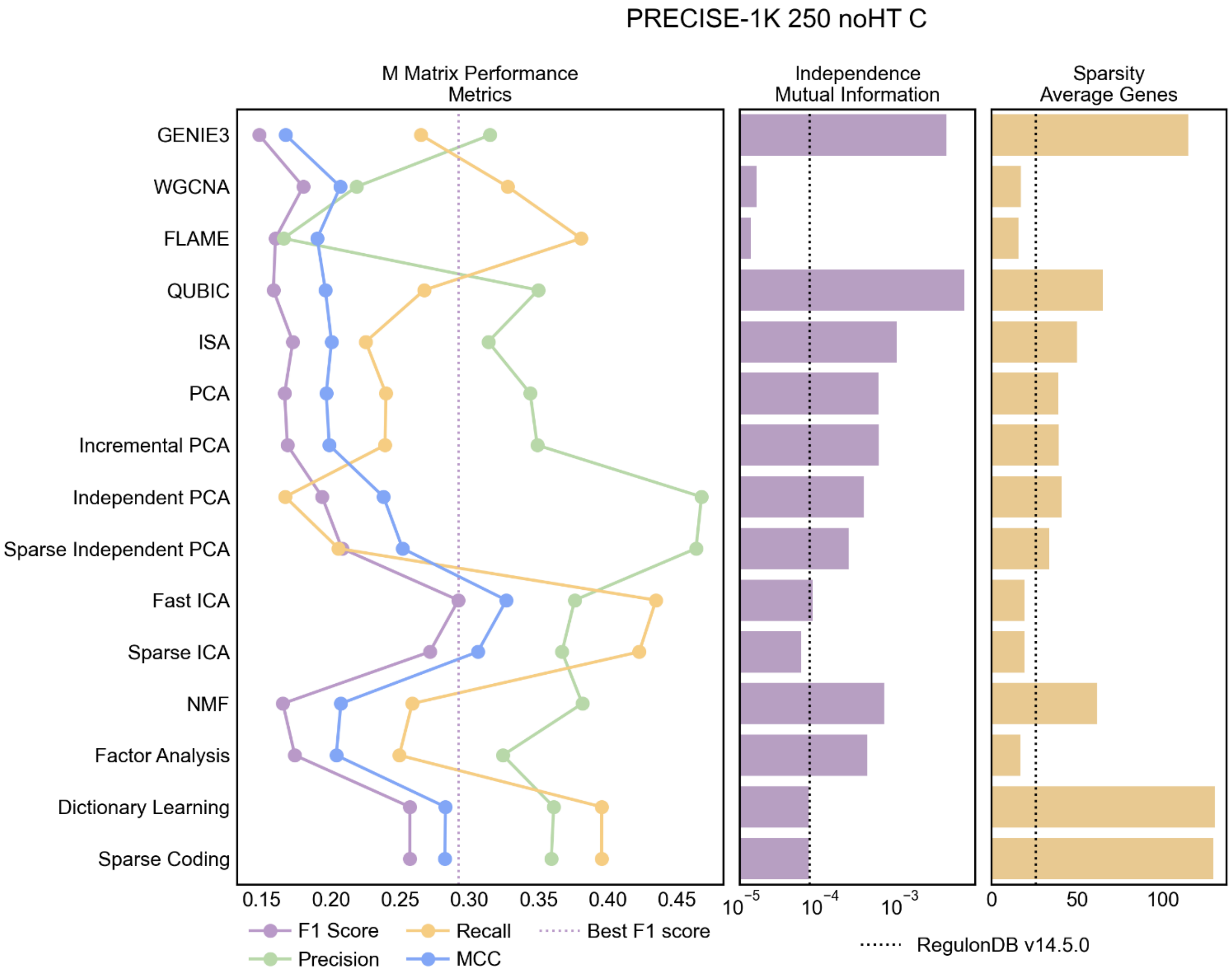


**Figure S9. Benchmark for the inference methods on PRECISE-1K (250 Components, Confirmed, excluding all HT methodologies)**

Benchmark for the inference methods on PRECISE-1K (250 Components, Confirmed, excluding all HT methodologies). Average Precision, Average Recall, Average F1 score, and Average Matthews Correlation Coefficient (MCC) were used for the M matrix benchmark. For the Network-only methods, we evaluated GENIE3 (direct network inference), WGCNA (clustering) and QUBIC (biclustering). A total of ten matrix factorization methods were examined covering Principal Component Analysis (PCA), Independent PCA, Sparse Independent PCA, Incremental PCA, FastICA, Sparse ICA, Non-negative matrix factorization (NMF), Factor Analysis, Dictionary Learning, and Sparse Coding. The M matrices obtained from these methods were binarized following the approach described by Sastry et al. The methods are evaluated based on their ability to accurately capture regulons from RegulonDB v14.5.0 Confirmed (C) interactions excluding all HT methodologies (see Methods section for details). The average mutual information and sparsity of the M matrices from various TRN inference methods were calculated. The values from RegulonDB TRN were plotted on the vertical dashed lines as a reference.

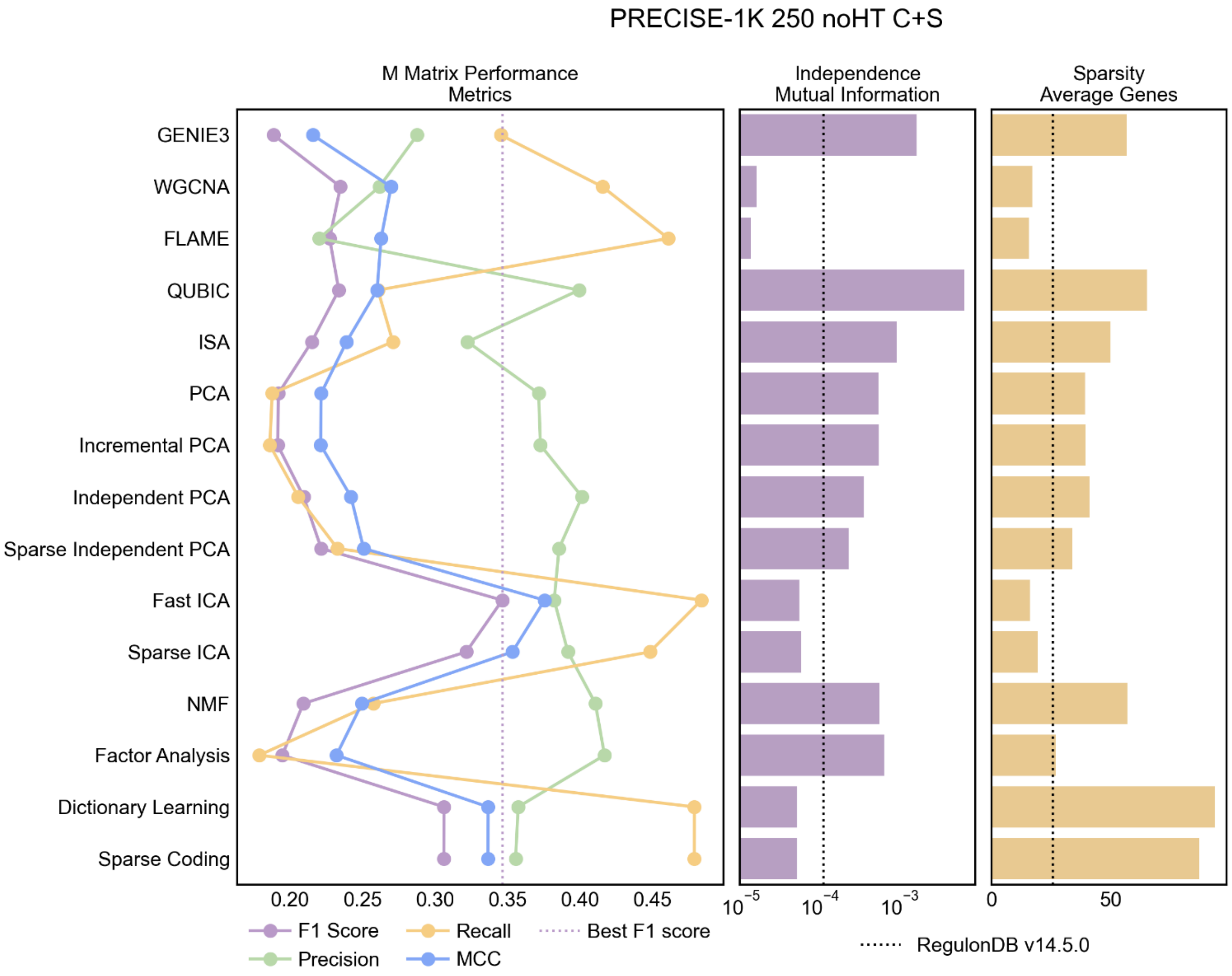


**Figure S10. Benchmark for the inference methods on PRECISE-1K (250 Components, Confirmed+Strong, excluding all HT methodologies)**

Benchmark for the inference methods on PRECISE-1K (250 Components, Confirmed+Strong, excluding all HT methodologies). Average Precision, Average Recall, Average F1 score, and Average Matthews Correlation Coefficient (MCC) were used for the M matrix benchmark. For the Network-only methods, we evaluated GENIE3 (direct network inference), WGCNA (clustering) and QUBIC (biclustering). A total of ten matrix factorization methods were examined covering Principal Component Analysis (PCA), Independent PCA, Sparse Independent PCA, Incremental PCA, FastICA, Sparse ICA, Non-negative matrix factorization (NMF), Factor Analysis, Dictionary Learning, and Sparse Coding. The M matrices obtained from these methods were binarized following the approach described by Sastry et al. The methods are evaluated based on their ability to accurately capture regulons from RegulonDB v14.5.0 Confirmed+Strong (C+S) interactions excluding all HT methodologies (see Methods section for details). The average mutual information and sparsity of the M matrices from various TRN inference methods were calculated. The values from RegulonDB TRN were plotted on the vertical dashed lines as a reference.

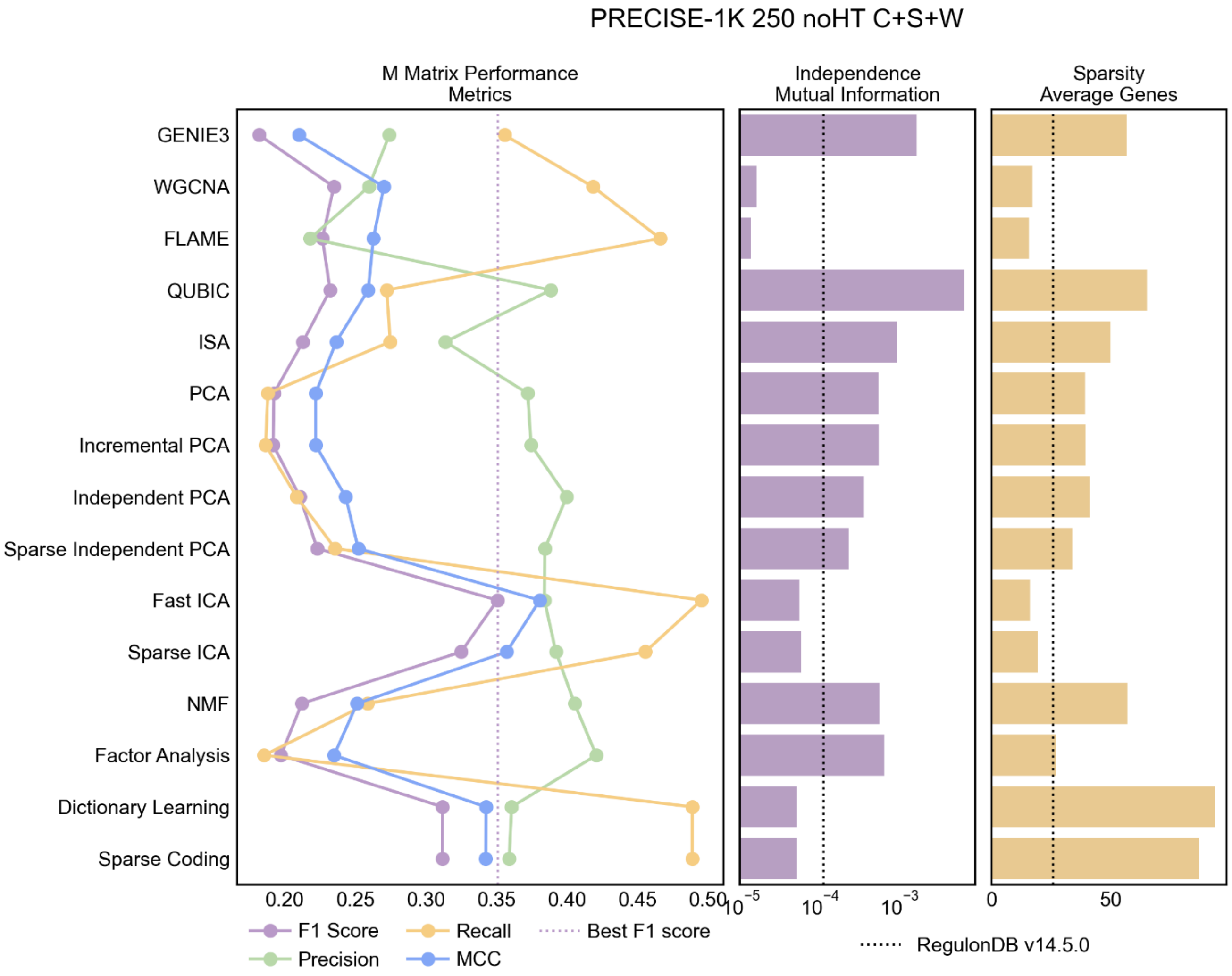


**Figure S11. Benchmark for the inference methods on PRECISE-1K (250 Components, Confirmed+Strong+Weak, excluding all HT methodologies)**

Benchmark for the inference methods on PRECISE-1K (250 Components, Confirmed+Strong+Weak, excluding all HT methodologies). Average Precision, Average Recall, Average F1 score, and Average Matthews Correlation Coefficient (MCC) were used for the M matrix benchmark. For the Network-only methods, we evaluated GENIE3 (direct network inference), WGCNA (clustering) and QUBIC (biclustering). A total of ten matrix factorization methods were examined covering Principal Component Analysis (PCA), Independent PCA, Sparse Independent PCA, Incremental PCA, FastICA, Sparse ICA, Non-negative matrix factorization (NMF), Factor Analysis, Dictionary Learning, and Sparse Coding. The M matrices obtained from these methods were binarized following the approach described by Sastry et al. The methods are evaluated based on their ability to accurately capture regulons from RegulonDB v14.5.0 Confirmed+Strong+Weak (C+S+W) interactions excluding all HT methodologies (see Methods section for details). The average mutual information and sparsity of the M matrices from various TRN inference methods were calculated. The values from RegulonDB TRN were plotted on the vertical dashed lines as a reference.

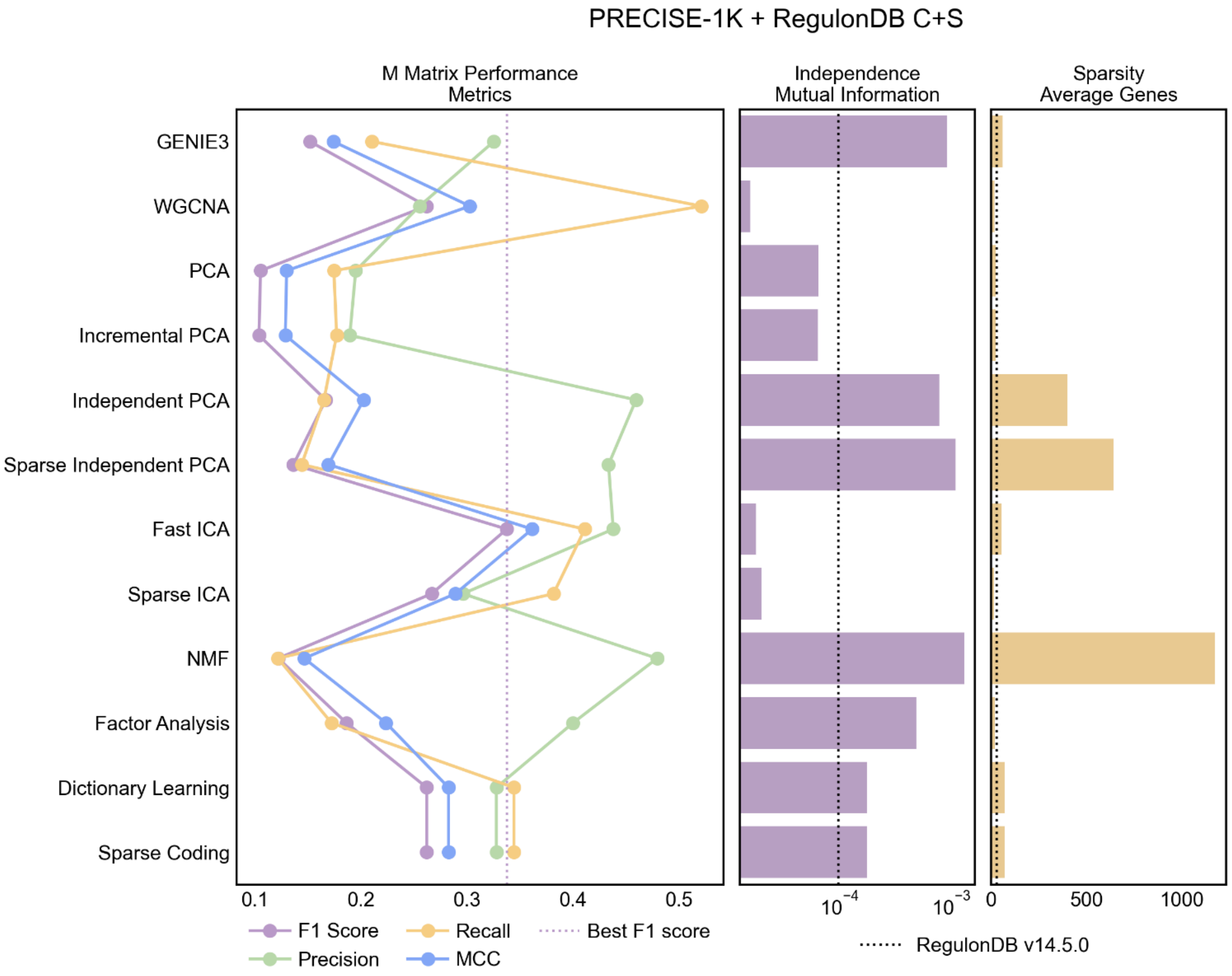


**Figure S12. Benchmark for the inference methods on PRECISE-1K + RegulonDB RNA-seq (2687 samples)**

Benchmark for the inference methods on PRECISE-1K + RegulonDB RNA-seq (2687 samples, 250 Components, Confirmed+Strong reference) Average Precision, Average Recall, Average F1 score, and Average Matthews Correlation Coefficient (MCC) were used for the M matrix benchmark. For the Network-only methods, we evaluated GENIE3 (direct network inference), WGCNA (clustering) and QUBIC (biclustering). A total of ten matrix factorization methods were examined covering Principal Component Analysis (PCA), Independent PCA, Sparse Independent PCA, Incremental PCA, FastICA, Sparse ICA, Non-negative matrix factorization (NMF), Factor Analysis, Dictionary Learning, and Sparse Coding. The M matrices obtained from these methods were binarized following the approach described by Sastry et al. The methods are evaluated based on their ability to accurately capture regulons from RegulonDB v14.5.0 Confirmed+Strong+Weak (C+S+W) interactions excluding all HT methodologies (see Methods section for details). The average mutual information and sparsity of the M matrices from various TRN inference methods were calculated. The values from RegulonDB TRN were plotted on the vertical dashed lines as a reference.

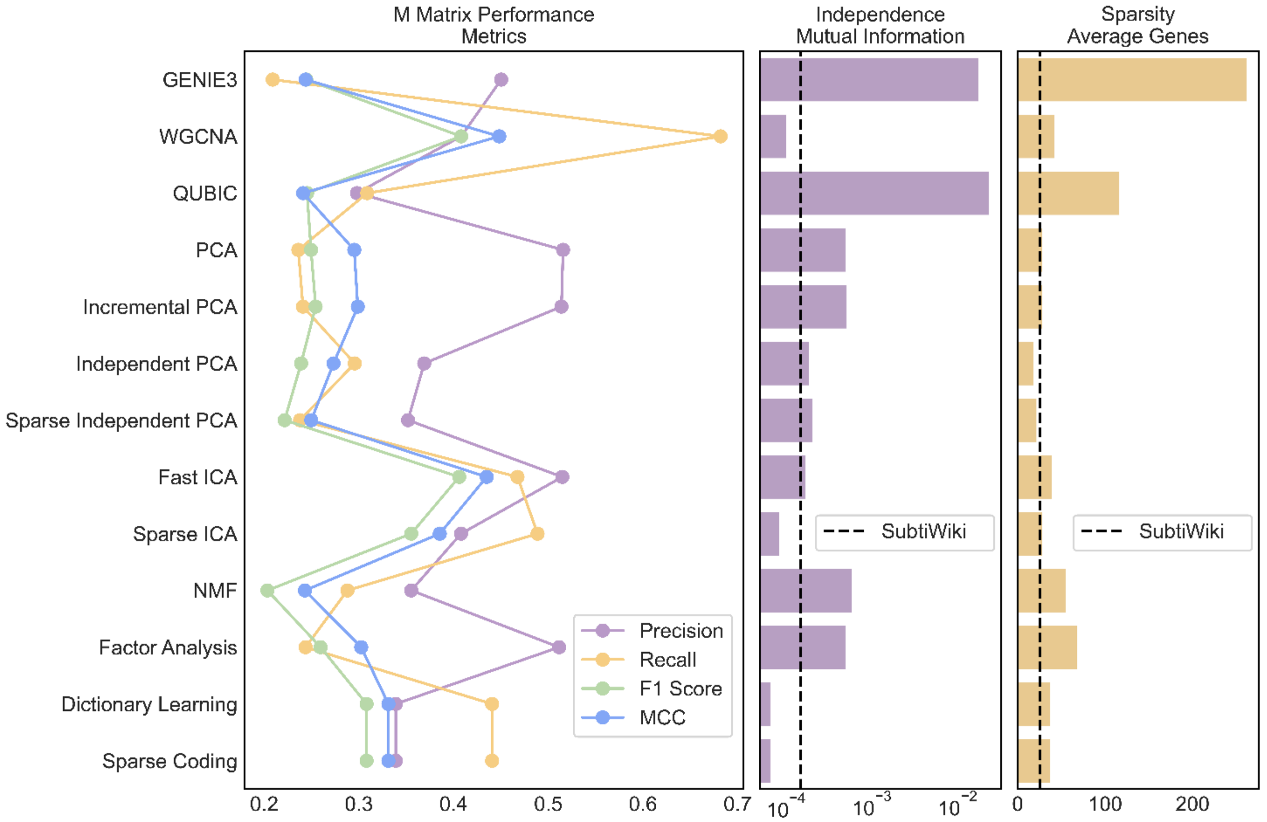


**Figure S13. Benchmark for the inference methods on *B. subtilis* (100 Components)**

Benchmark for the inference methods on *B. subtilis* (100 Components). Average Precision, Average Recall, Average F1 score, and Average Matthews Correlation Coefficient (MCC) were used for the M matrix benchmark. For the Network-only methods, we evaluated GENIE3 (direct network inference), WGCNA (clustering) and QUBIC (biclustering). A total of ten matrix factorization methods were examined covering Principal Component Analysis (PCA), Independent PCA, Sparse Independent PCA, Incremental PCA, FastICA, Sparse ICA, Non-negative matrix factorization (NMF), Factor Analysis, Dictionary Learning, and Sparse Coding. The M matrices obtained from these methods were binarized following the approach described by Sastry et al. The methods are evaluated based on their ability to accurately capture regulons from SubtiWiki (see Methods section for details). The average mutual information and sparsity of the M matrices from various TRN inference methods were calculated. The values from SubtiWiki TRN were plotted on the vertical dashed lines as a reference.

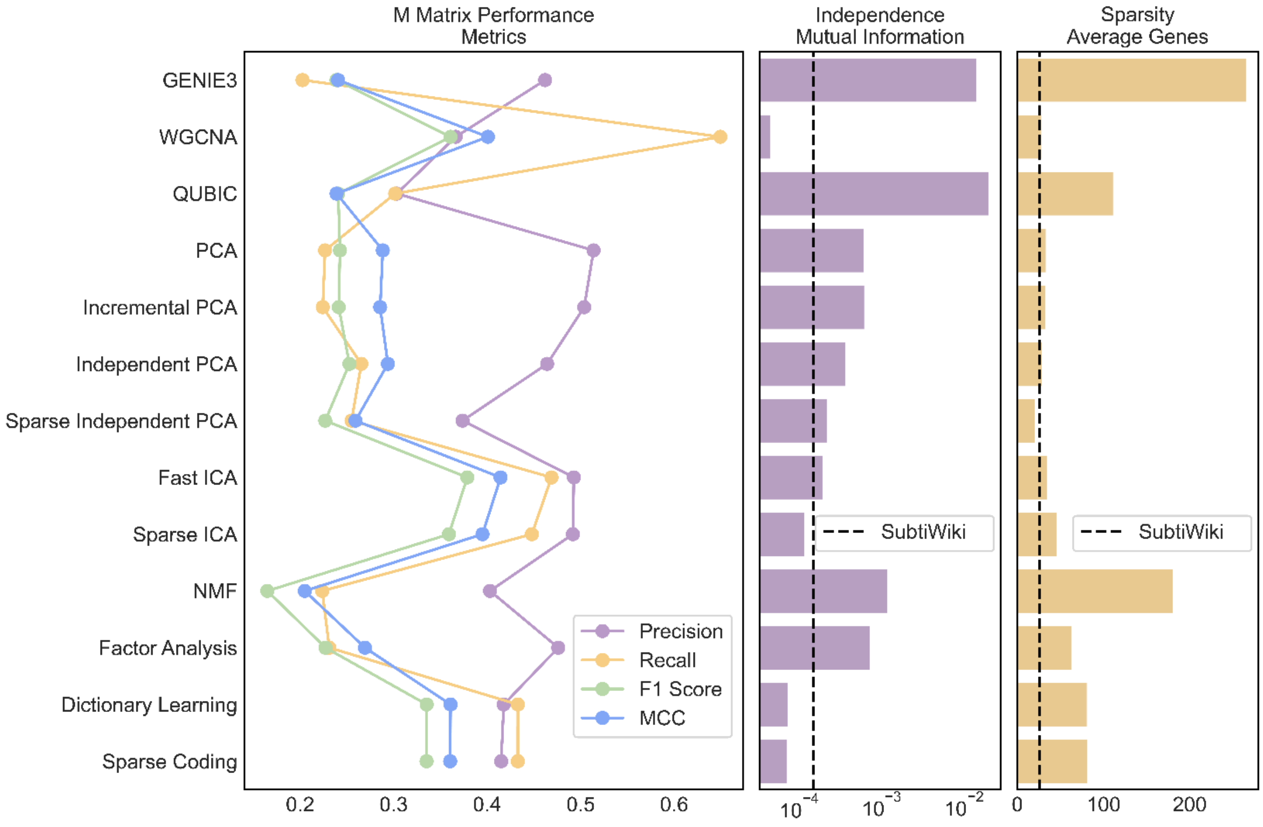


**Figure S14. Benchmark for the inference methods on *B. subtilis* (150 Components)**

Benchmark for the inference methods on *B. subtilis* (150 Components). Average Precision, Average Recall, Average F1 score, and Average Matthews Correlation Coefficient (MCC) were used for the M matrix benchmark. For the Network-only methods, we evaluated GENIE3 (direct network inference), WGCNA (clustering) and QUBIC (biclustering). A total of ten matrix factorization methods were examined covering Principal Component Analysis (PCA), Independent PCA, Sparse Independent PCA, Incremental PCA, FastICA, Sparse ICA, Non-negative matrix factorization (NMF), Factor Analysis, Dictionary Learning, and Sparse Coding. The M matrices obtained from these methods were binarized following the approach described by Sastry et al. The methods are evaluated based on their ability to accurately capture regulons from SubtiWiki (see Methods section for details). The average mutual information and sparsity of the M matrices from various TRN inference methods were calculated. The values from SubtiWiki TRN were plotted on the vertical dashed lines as a reference.

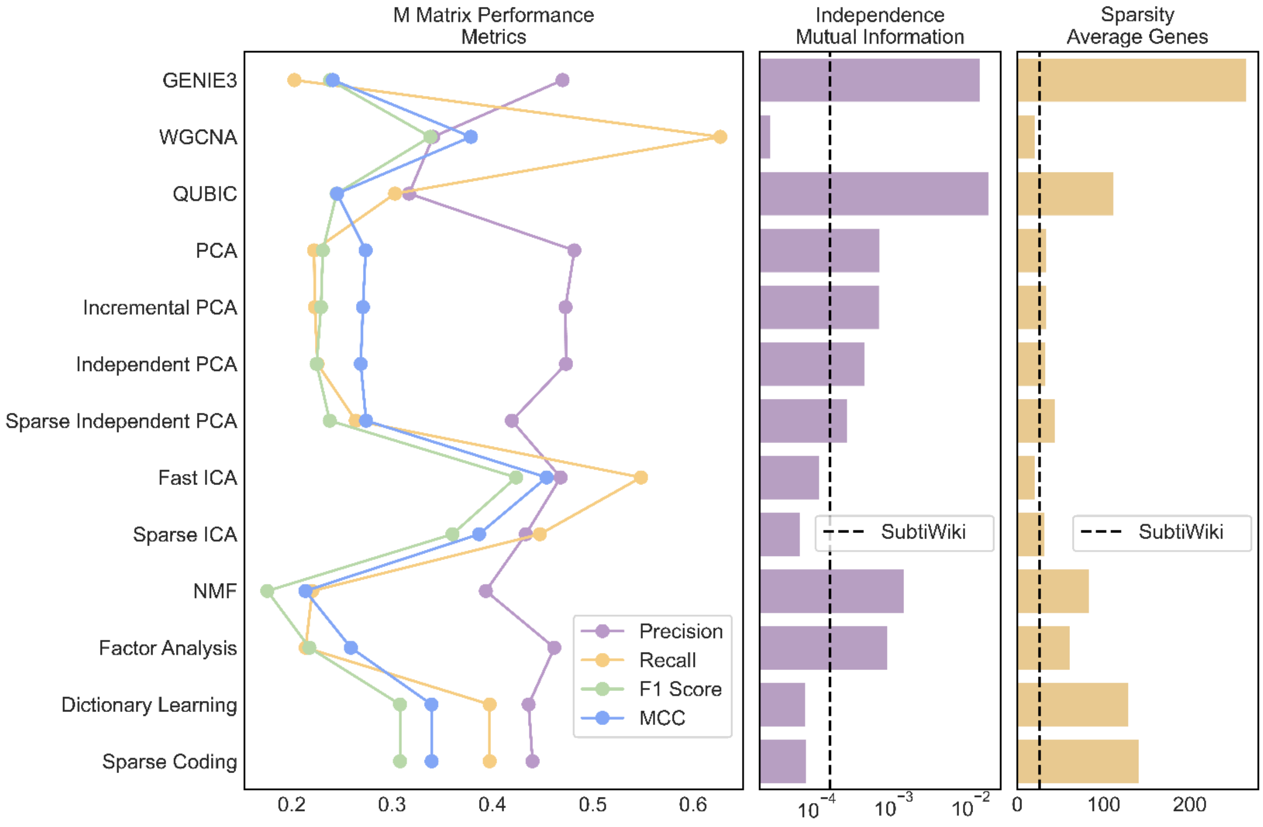


**Figure S15. Benchmark for the inference methods on *B. subtilis* (200 Components)**

Benchmark for the inference methods on *B. subtilis* (200 Components). Average Precision, Average Recall, Average F1 score, and Average Matthews Correlation Coefficient (MCC) were used for the M matrix benchmark. For the Network-only methods, we evaluated GENIE3 (direct network inference), WGCNA (clustering) and QUBIC (biclustering). A total of ten matrix factorization methods were examined covering Principal Component Analysis (PCA), Independent PCA, Sparse Independent PCA, Incremental PCA, FastICA, Sparse ICA, Non-negative matrix factorization (NMF), Factor Analysis, Dictionary Learning, and Sparse Coding. The M matrices obtained from these methods were binarized following the approach described by Sastry et al. The methods are evaluated based on their ability to accurately capture regulons from SubtiWiki (see Methods section for details). The average mutual information and sparsity of the M matrices from various TRN inference methods were calculated. The values from SubtiWiki TRN were plotted on the vertical dashed lines as a reference.

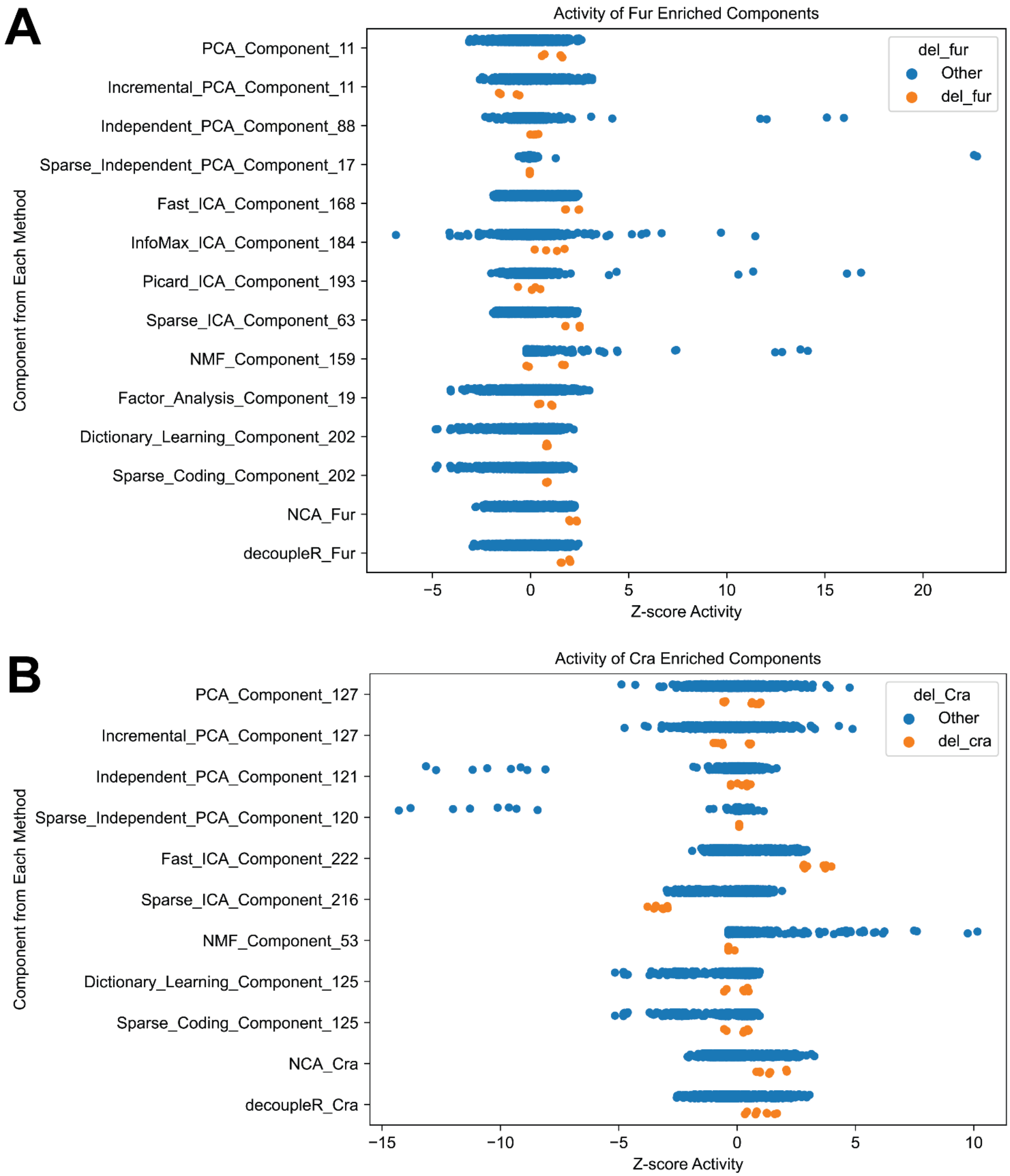


**Figure S16. Comparison of the activities inferred by Network-and-Activity methods and Activity-only methods.**

**A)** Activities of components enriched for the Fur regulon as identified through inference methods. **B)** Activities of components enriched for the Cra regulon identified through inference methods. For the deletion samples, the activity will be either highest or lowest, depending on the sign selection of the M components, where the component with the largest absolute weight

is set to be positive (Activity values would be flipped together with the weights). Note that Factor Analysis are excluded as no Cra-enriched components were detected using these methods. Notably, the NCA and decoupleR activities inferred from the Cra regulon did not show extreme values upon Cra deletion, unlike the Fur deletion. This discrepancy may arise because Cra functions as a dual regulator, making activity inference based on the entire regulon potentially unreliable.

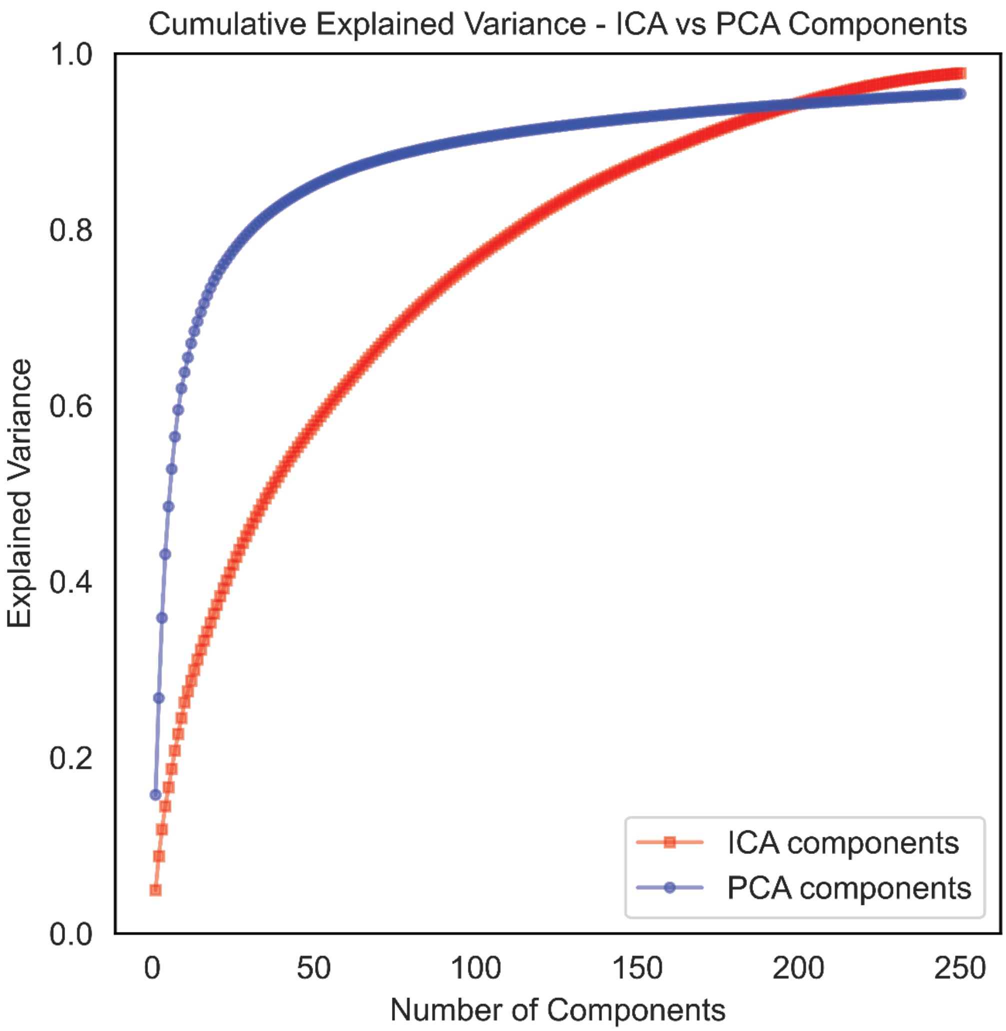


**Figure S17. Cumulative explained variance for ICA and PCA.**

Cumulative explained variance for the 250 components from ICA and PCA. Red curve represents the ICA explained variance, Blue curve represents the PCA explained variance, the values are sorted from high to low.

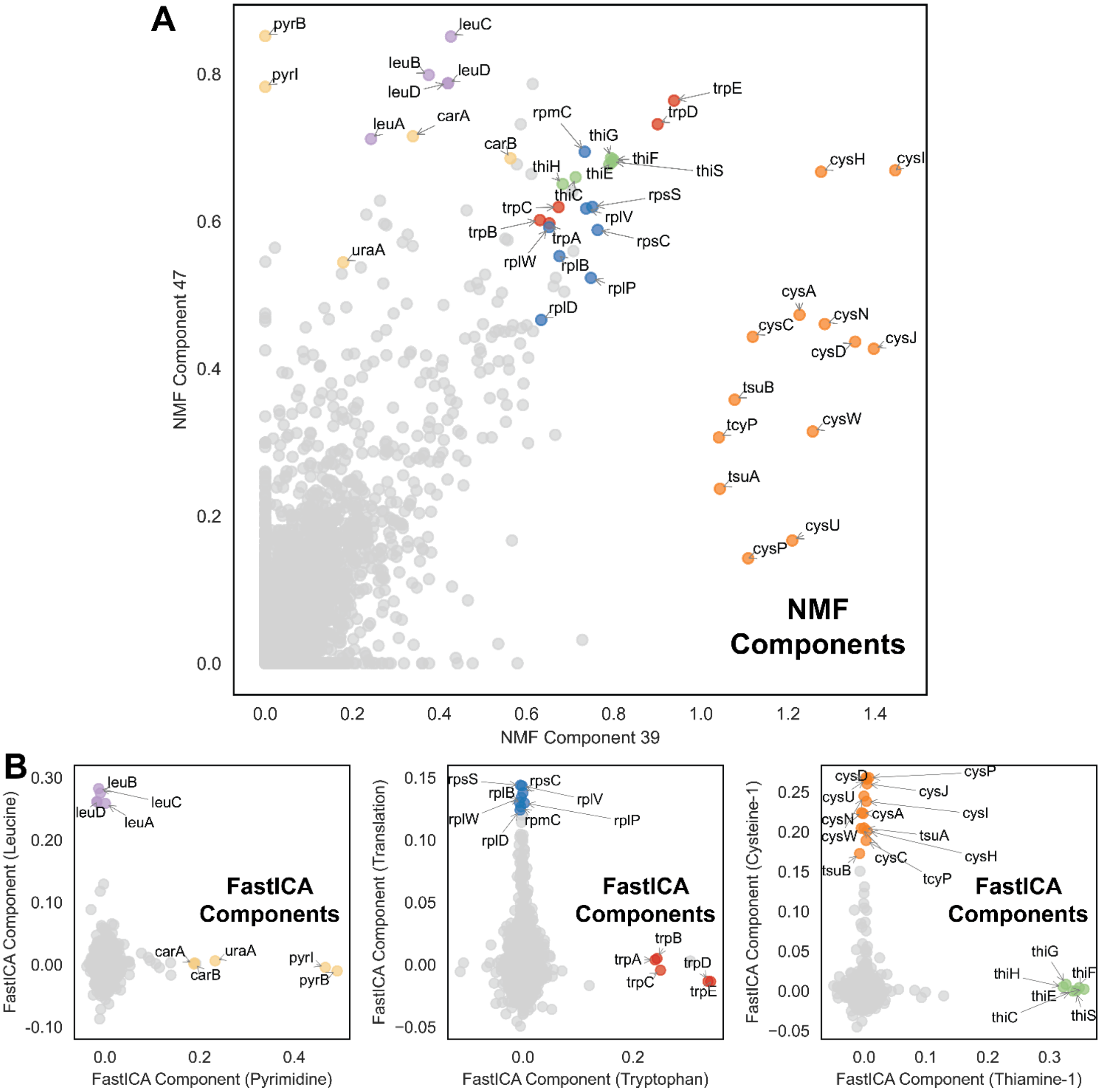


586 **Figure S18. Comparison between NMF components and FastICA components.**

587 **A)** Scatterplot of the gene weights of two components from NMF. **B)** Independent components
588 corresponding to sets of genes within the two NMF components highlighted in Panel A.

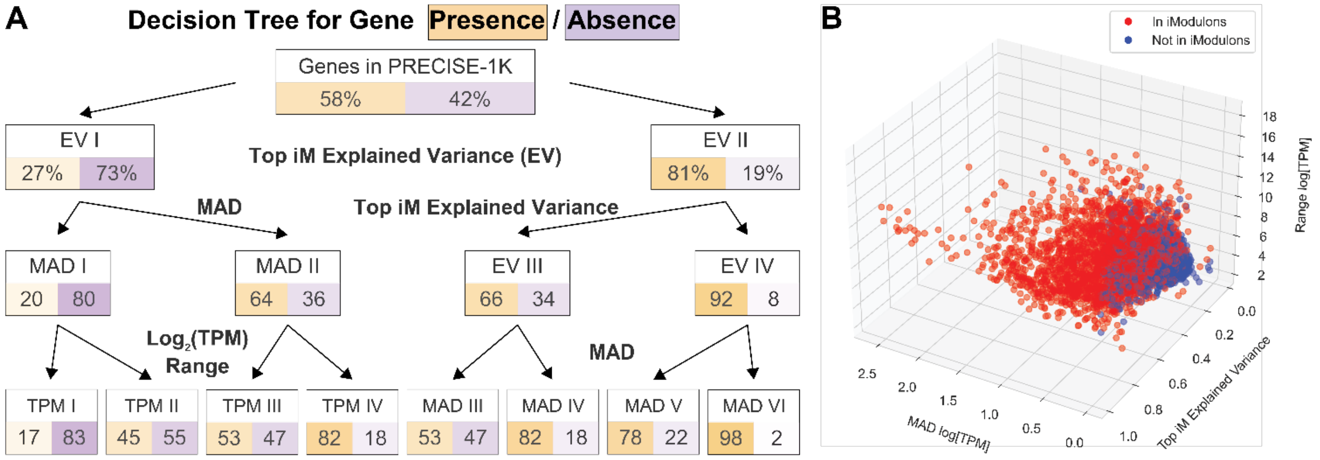


**Figure S19. Selection of the metrics to characterize the genes that were captured by iModulons.**

**A)** Decision Tree of the gene presence/absence in the iModulons. Top iModulon explained variance and MAD are the most deterministic from 7 variables - Top iModulon explained variance (Importance: 0.426), Top 2 iModulon explained variance (Importance: 0.057), Top 3 iModulon explained variance (Importance: 0.067), range of the log tpm values (Importance: 0.103), MAD (Importance: 0.150), mean of the log tpm values (Importance: 0.097), total explained variance from all the iModulons (Importance: 0.070), and number of regulators as annotated in RegulonDB (Importance: 0.030). EV, explained variance; MAD, median absolute deviation; TPM, transcripts per million. Roman numerals indicate categories defined by the branches of the decision tree. **B)** Scatterplot of the MAD, Top iM explained variance and range of the log tpm values for all the genes in PRECISE-1K. Genes that were captured by iModulons were highlighted in red, the others were colored in blue.

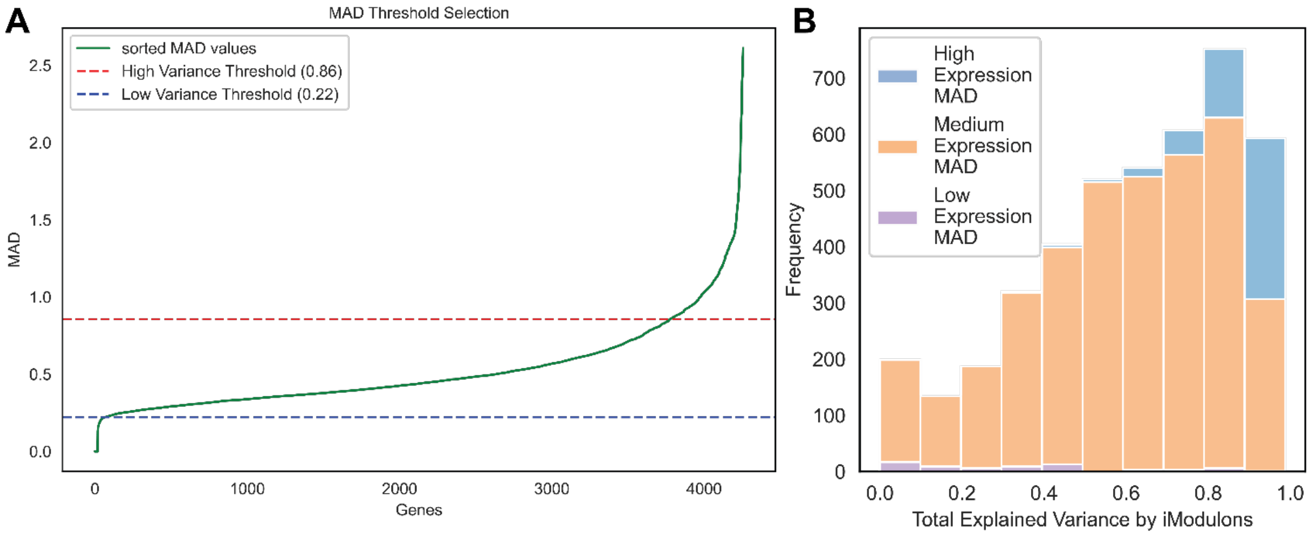


602 **Figure S20. Genes with high expression MAD were well-explained by iModulons.**

603 **A)** Distribution of the MAD (median absolute deviation) of the expression level for all the genes
604 in PRECISE-1K. Elbows were selected to determine High/Medium/Low expression MAD. **B)**
605 Histogram of the total explained variance by iModulons for all the genes.

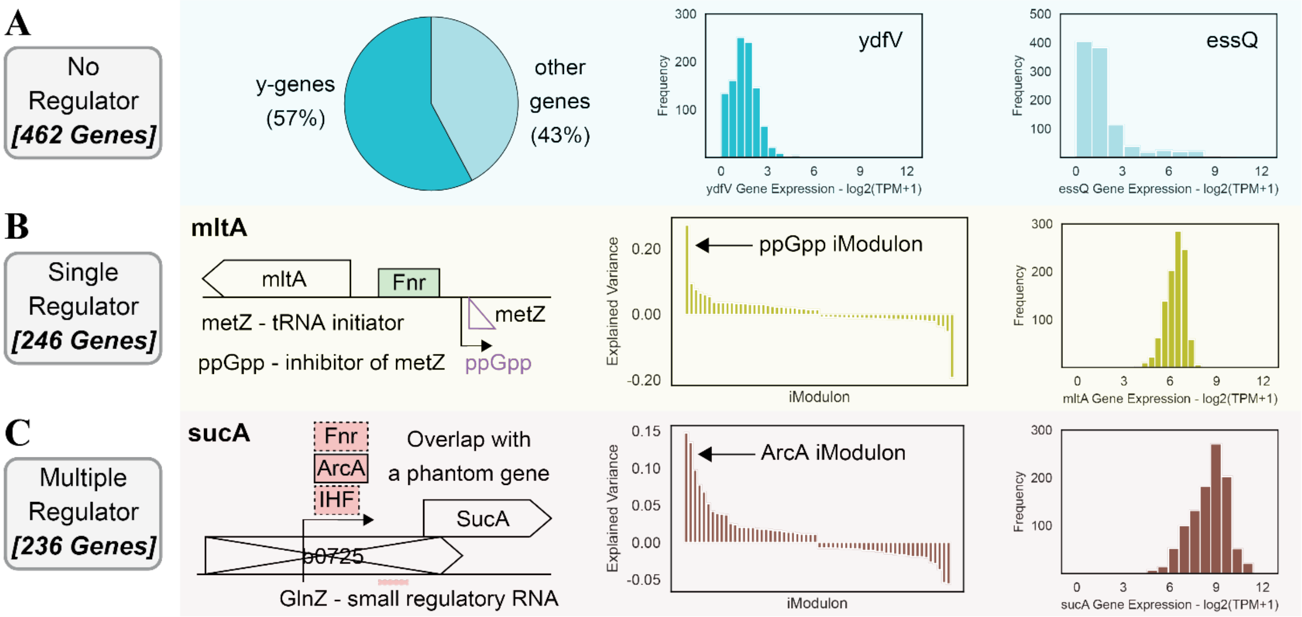


**Figure S21. Genes that are not well-captured by iModulons but activated in specific conditions.**

**A)** Genes with no confirmed regulators. Approximately 57% of these are y-genes. For example, *ydfV* and *essQ* have no annotated regulator binding sites according to RegulonDB. **B)** Genes with one confirmed regulator in RegulonDB. The iModulon with the highest explained variance contribution for *mltA* is ppGpp, suggesting that the expression of *mltA* is primarily driven by ppGpp rather than by Fnr. **C)** Genes with more than one confirmed regulator in RegulonDB. *sucA* has three annotated binding sites, one of which overlaps with a pseudogene and the small regulatory RNA GlnZ.

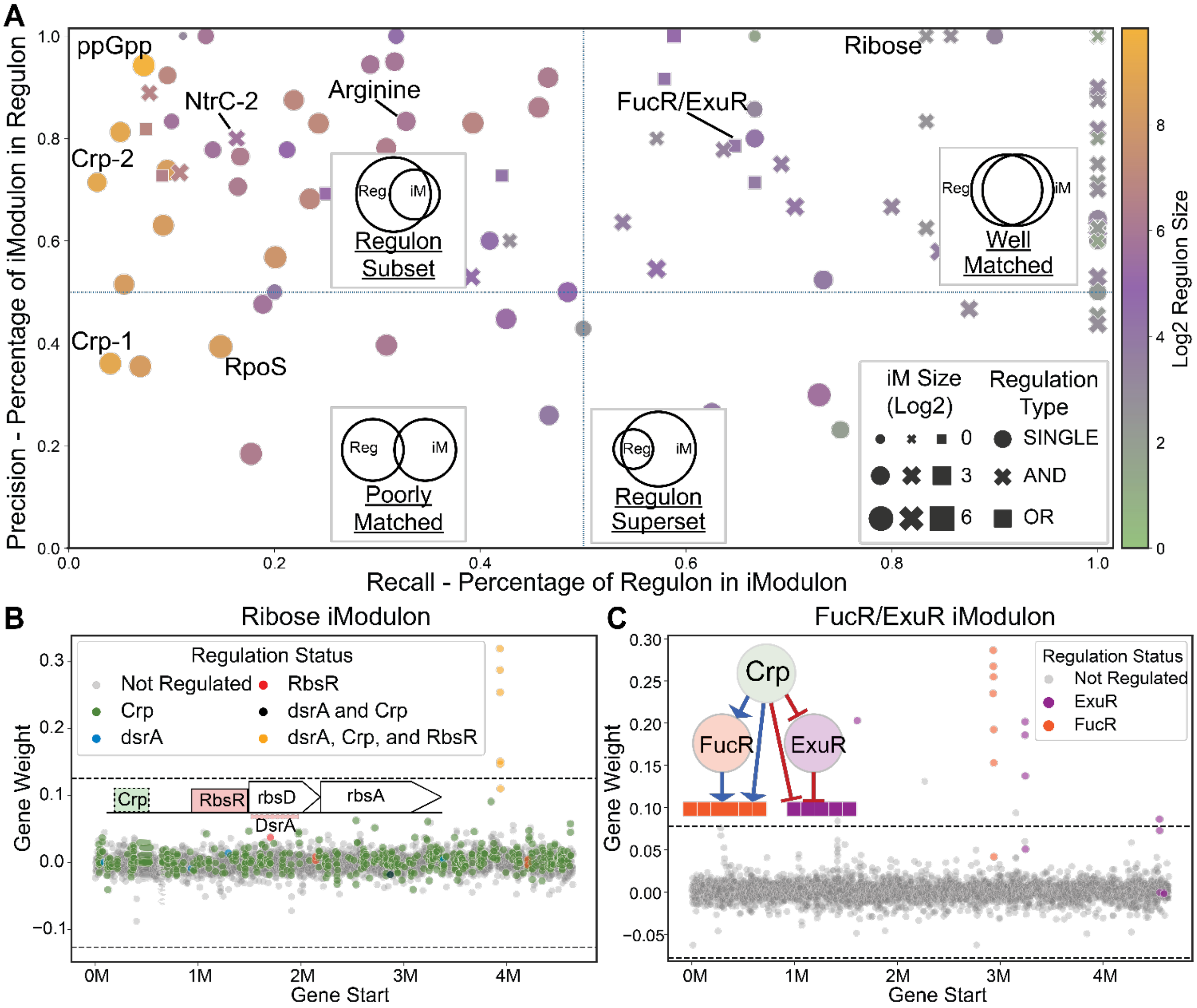


**Figure S22. iModulon performance on capturing known regulons**

**A)** Precision/Recall plot for all components in PRECISE-1K. Point size represents the size of the iModulon while color represents the size of the associated regulon (both in log2 scale). The shape of the point represents if the component has a single dominant regulator, multiple overlapping regulators, or multiple separate regulators. **B)** Gene weight plot of the Ribose iModulon. Point color represents the combination of regulators for a given gene. All of the genes regulated by DsrA, RbsR, and CRP are highly weighted in this iModulon as compared to genes regulated by a subset of these regulators. **C)** Gene weight plot of the FucR/ExuR iModulon. Point color represents the regulator of a given point. There is no overlap between genes regulated by ExuR and FucR, however all genes regulated by either regulator are regulated by CRP, as well as both ExuR and FucR being regulated by CRP.

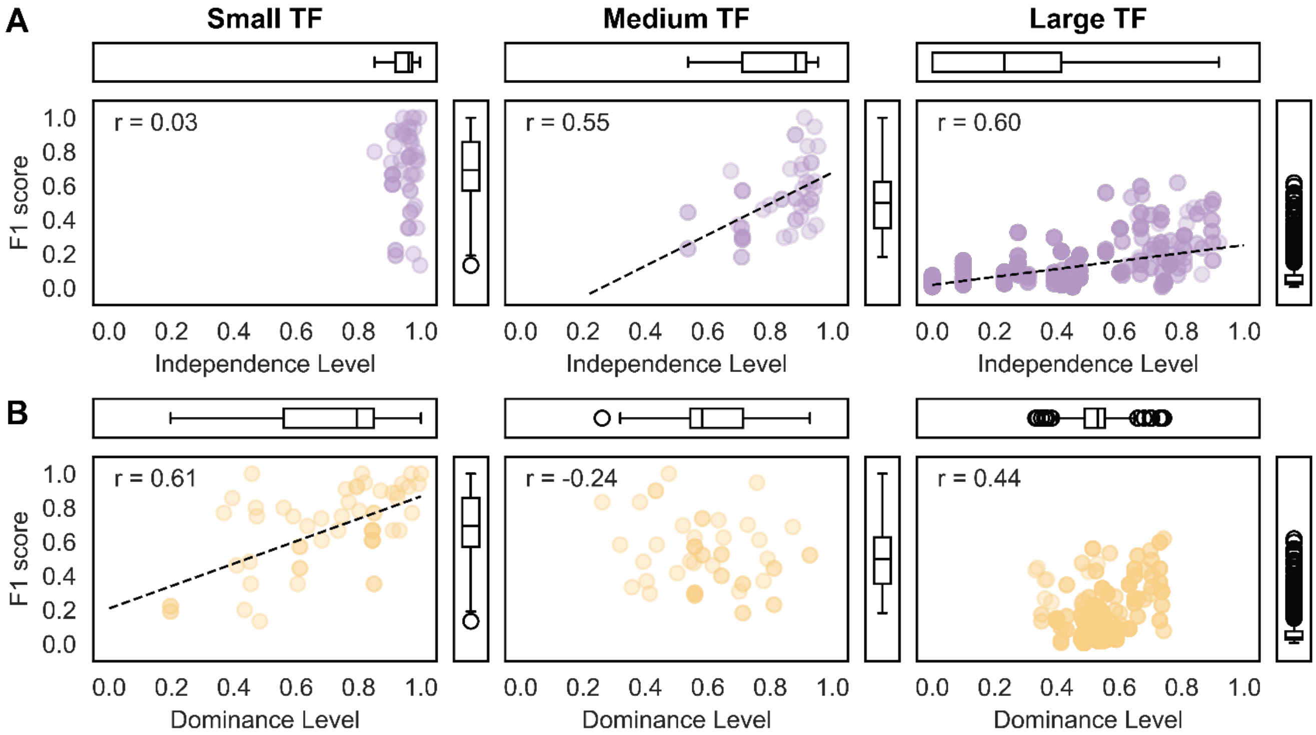


**Figure S23. Association of Regulon F1 Score with Independence and Dominance Levels**

**A)** Scatterplots relating the F1 score of each regulon against its independence level, stratified by regulon size. Independence level quantifies the regulatory independence of a regulon (higher values indicate greater independence) and was derived by normalizing the average mutual information of the regulon with other regulons in the TRN structure (see Methods). Subplots are categorized by regulon size: Small TF (size<10), Medium TF (10≤size≤30), and Large TF (size>30), the same as Figure 4B. **B)** Scatterplots relating the F1 score of each regulon to its dominance level. Dominance level reflects the degree to which genes in the regulon are controlled by a single regulator (higher values indicate stronger dominance). This metric was computed by normalizing the average of the top iModulon explained variances across all genes in the regulon (see Methods). Subplots are partitioned by regulon size as in (A).

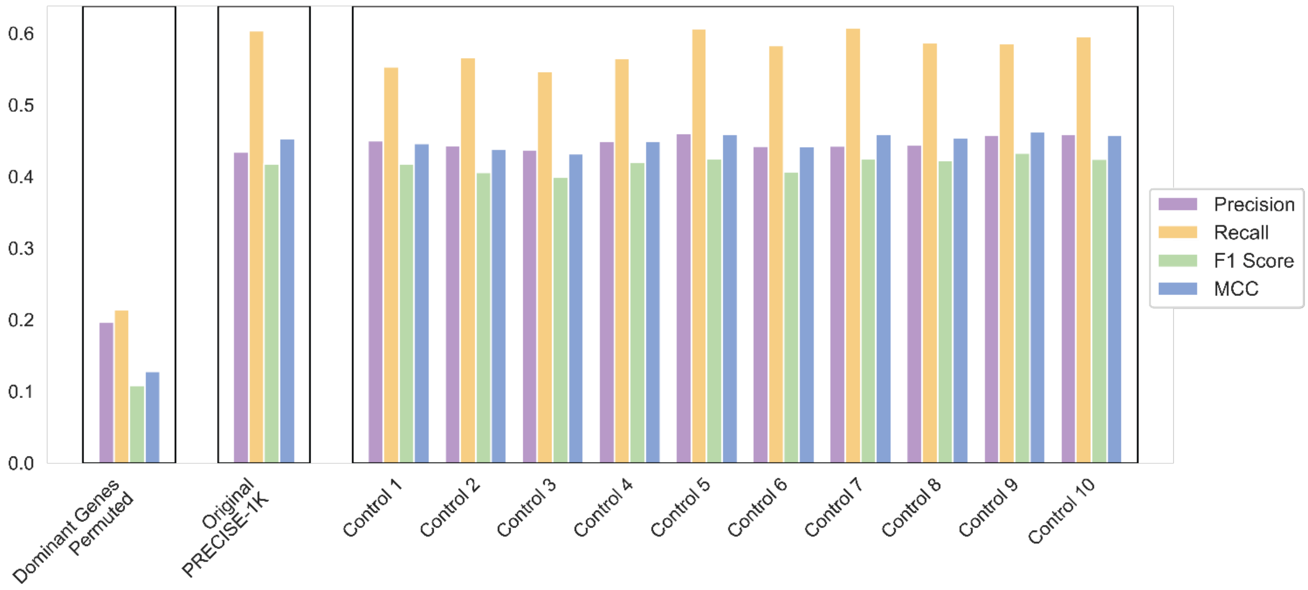


**Figure S24. Comparison of the performance of recapturing the regulons on original PRECISE-1K expression matrix, dominated genes permuted PRECISE-1K expression matrix, and non-dominated genes permuted PRECISE-1K expression matrix.**

Histogram of the Precision, Recall, F1 score, and MCC on capturing regulons on regulatory dominated genes (877 genes in total) permuted PRECISE-1K, original PRECISE-1K, and 10 other randomly selected gene set (877 genes in total) permuted PRECISE-1K as controls.

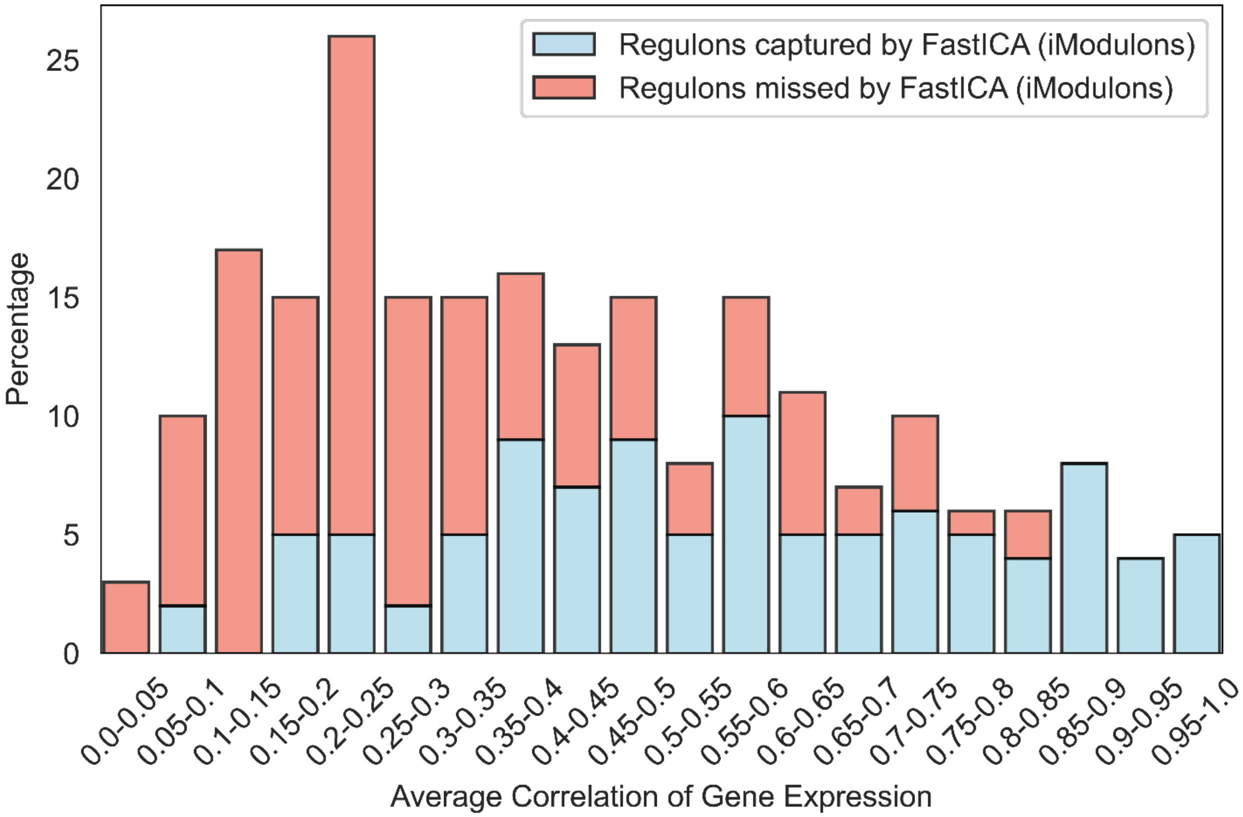


**Figure S25. Average Gene-Gene Correlation Within Each Regulon**

Average gene-gene correlations for the regulons were calculated by averaging the pairwise expression correlations among all genes within each regulon. Blue indicates regulons that were captured by iModulons, while red indicates those that were missed.

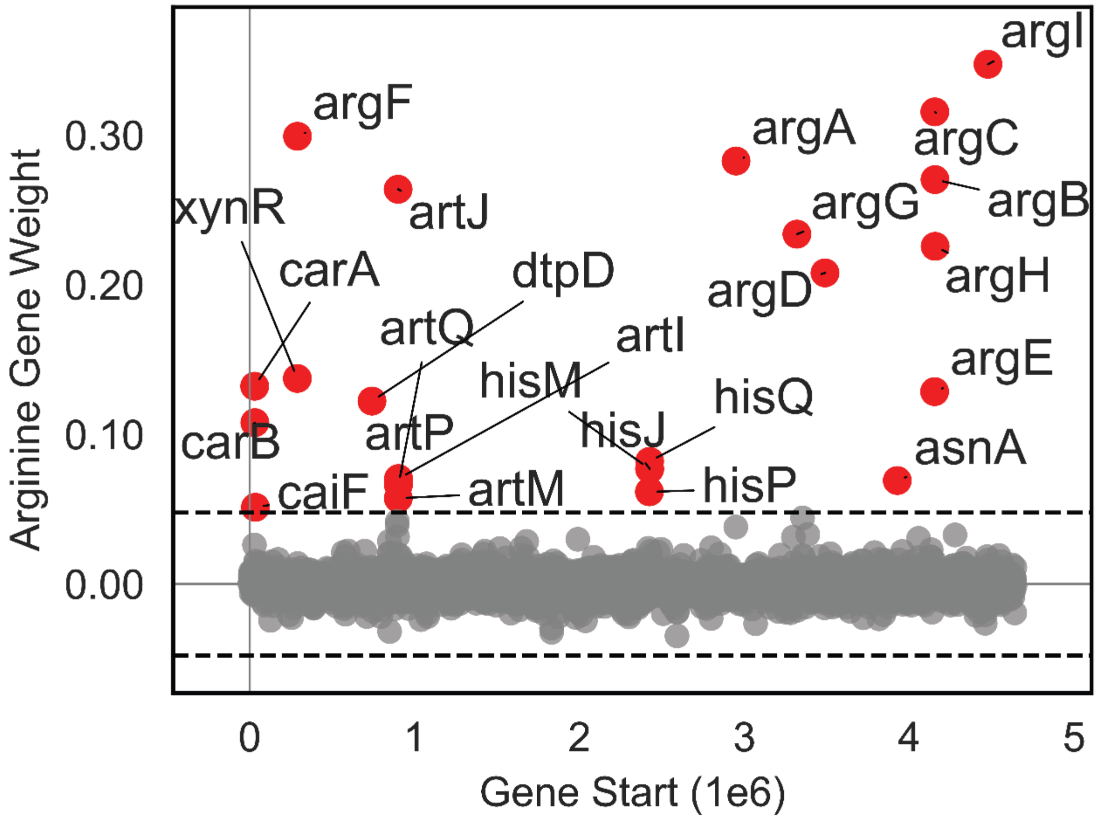


**Figure S26. Gene weights of the Arginine iModulon.**

Red colored genes are genes that are in the Arginine iModulon. Y axis is the gene weights from M matrix. X-axis is the location on the genome.

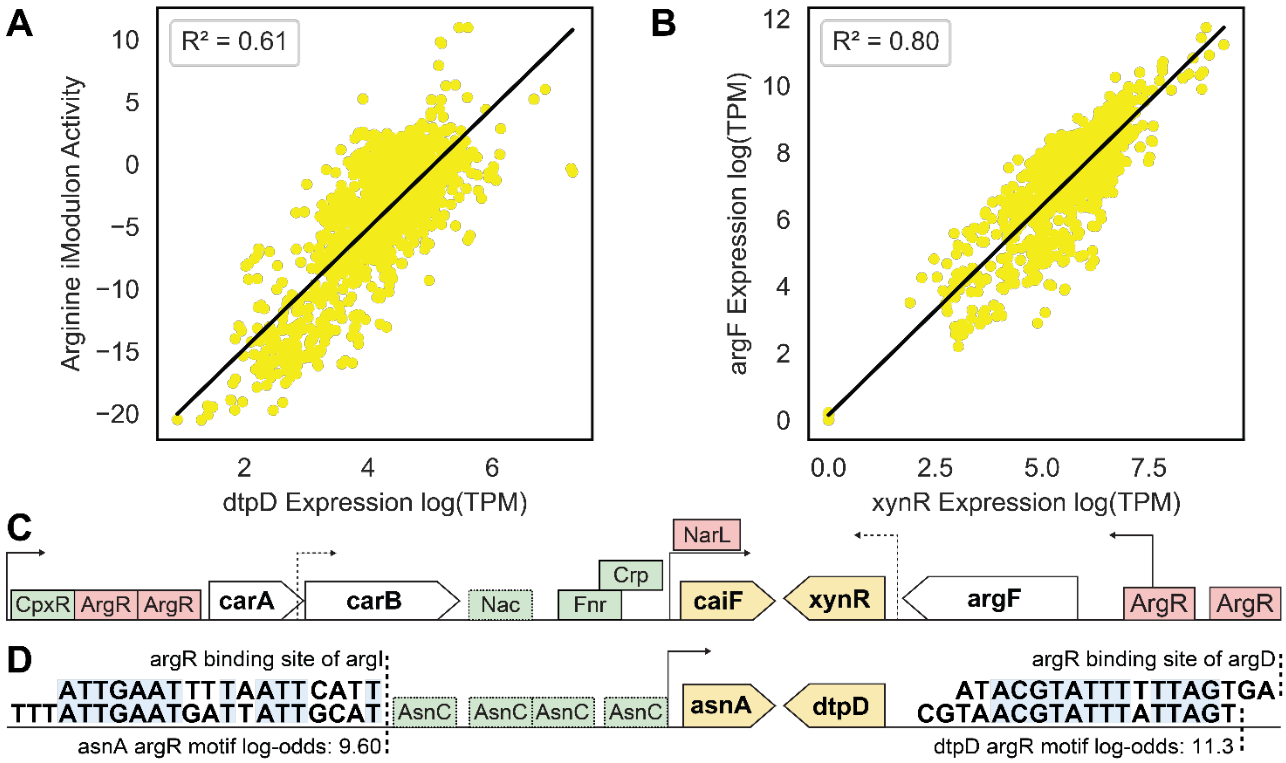


**Figure S27. Arginine iModulon Exclusive Genes**

A) Scatterplot of the *dtpD* gene expression and Arginine iModulon activity. B) Scatterplot of the *xynR* gene expression and the argF gene expression. C) Promoter diagrams of *caiF* and *xynR* (genes exclusive to the Arginine iModulon but not part of the ArgR regulon). D) Promoter diagrams of *asnA* and *dtpD* (genes exclusive to the Arginine iModulon but not part of the ArgR regulon).

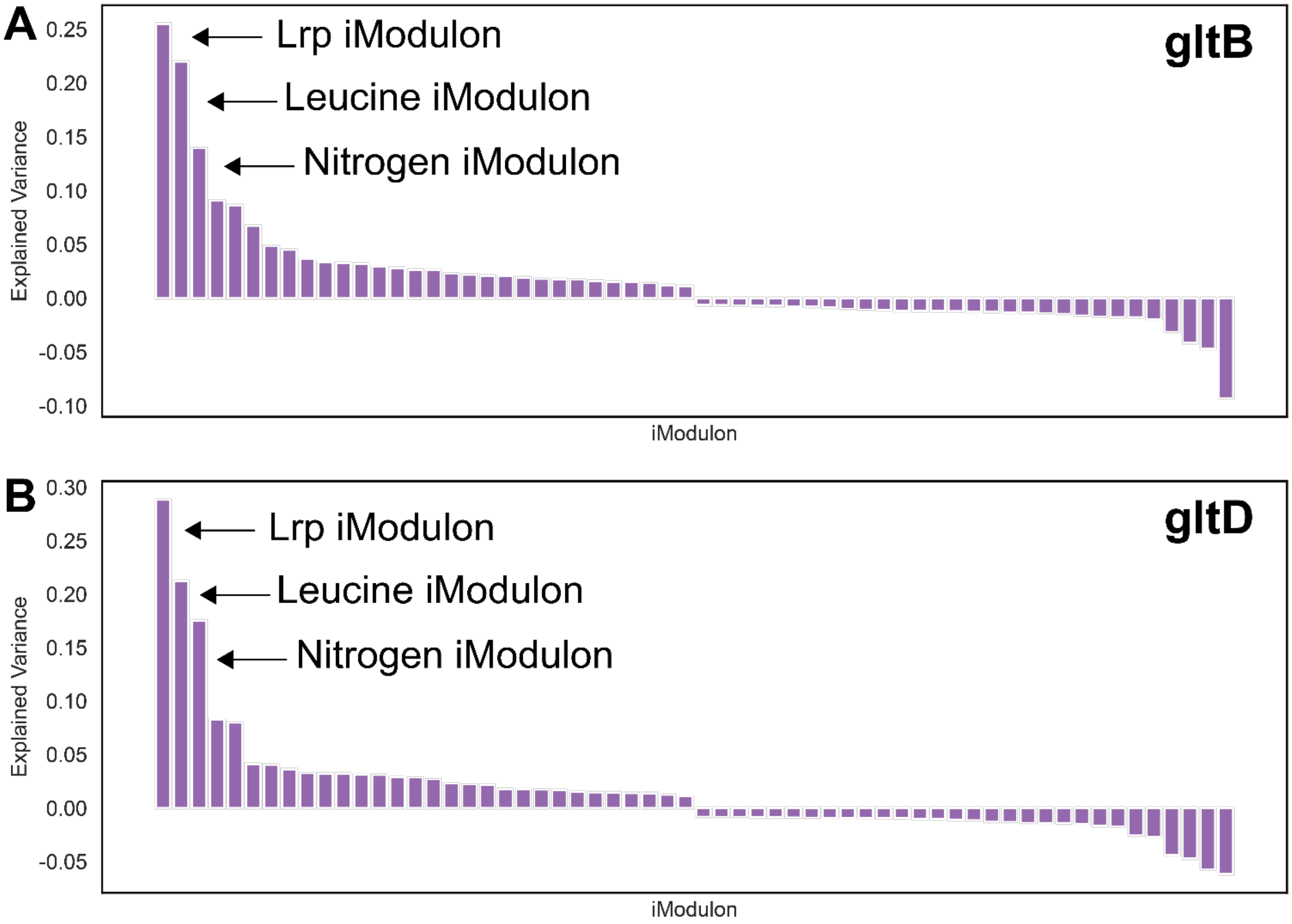


**Figure S28. Contributions to the total explained variance of gltB and gltD by iModulons.**

A) Barplot for the per component explained variance from the top 30 and bottom 30 iModulons for gltB. Lrp, Leucine, and Nitrogen are the top 3 iModulons. B) Barplot for the per component explained variance from the top 30 and bottom 30 iModulons for gltD. Lrp, Leucine, and Nitrogen are the top 3 iModulons.

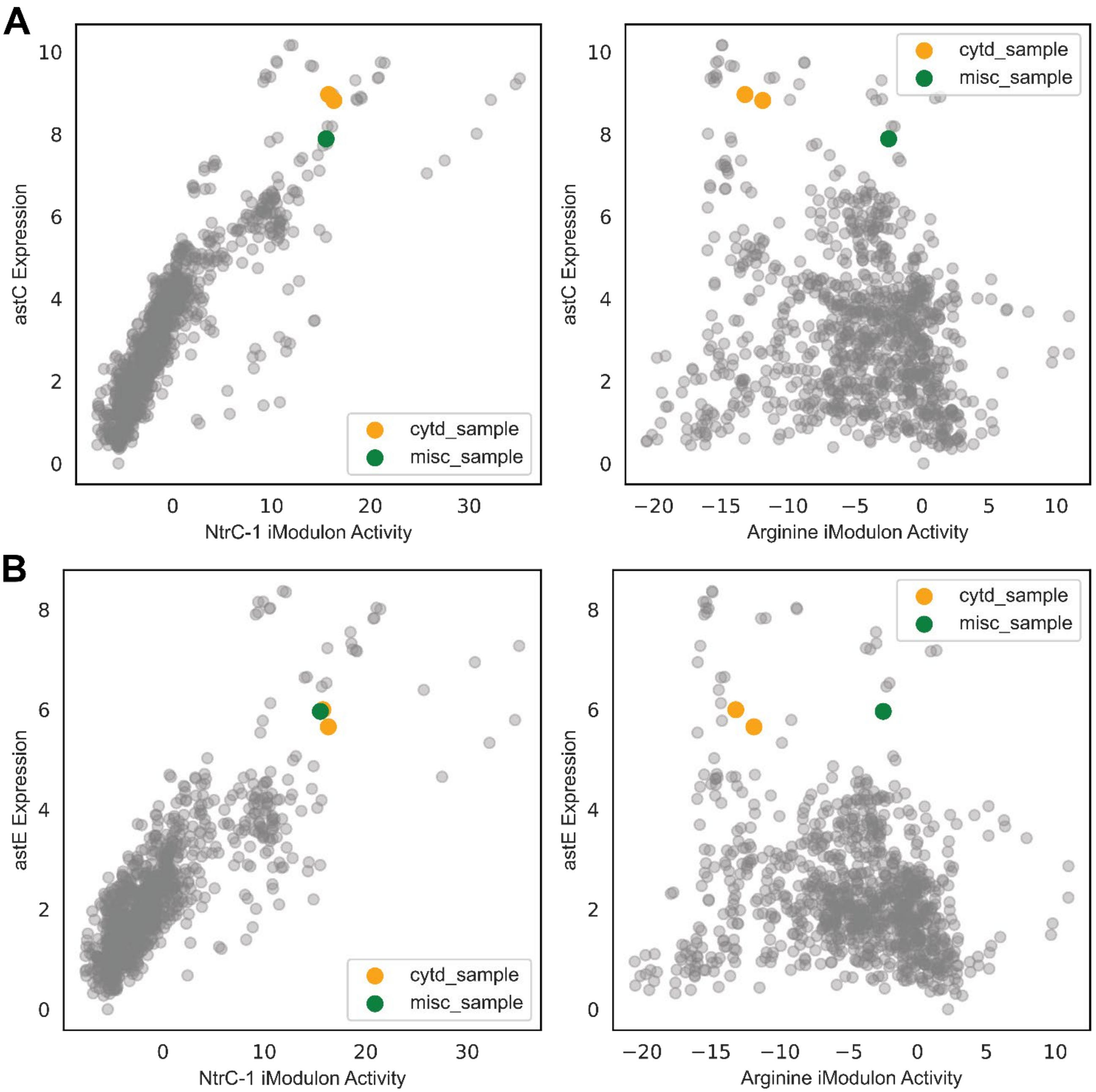


**Figure S29. Dominance of the NtrC on ast Operon**

A) Scatterplot of the astC gene expression versus NtrC-1 and Arginine iModulon activity. misc_sample is a low nitrogen condition on M9 base media and the cytd_sample is a cytidine (1mM) added condition on M9 base media. B) Scatterplot of the astE gene expression versus NtrC-1 and Arginine iModulon activity.

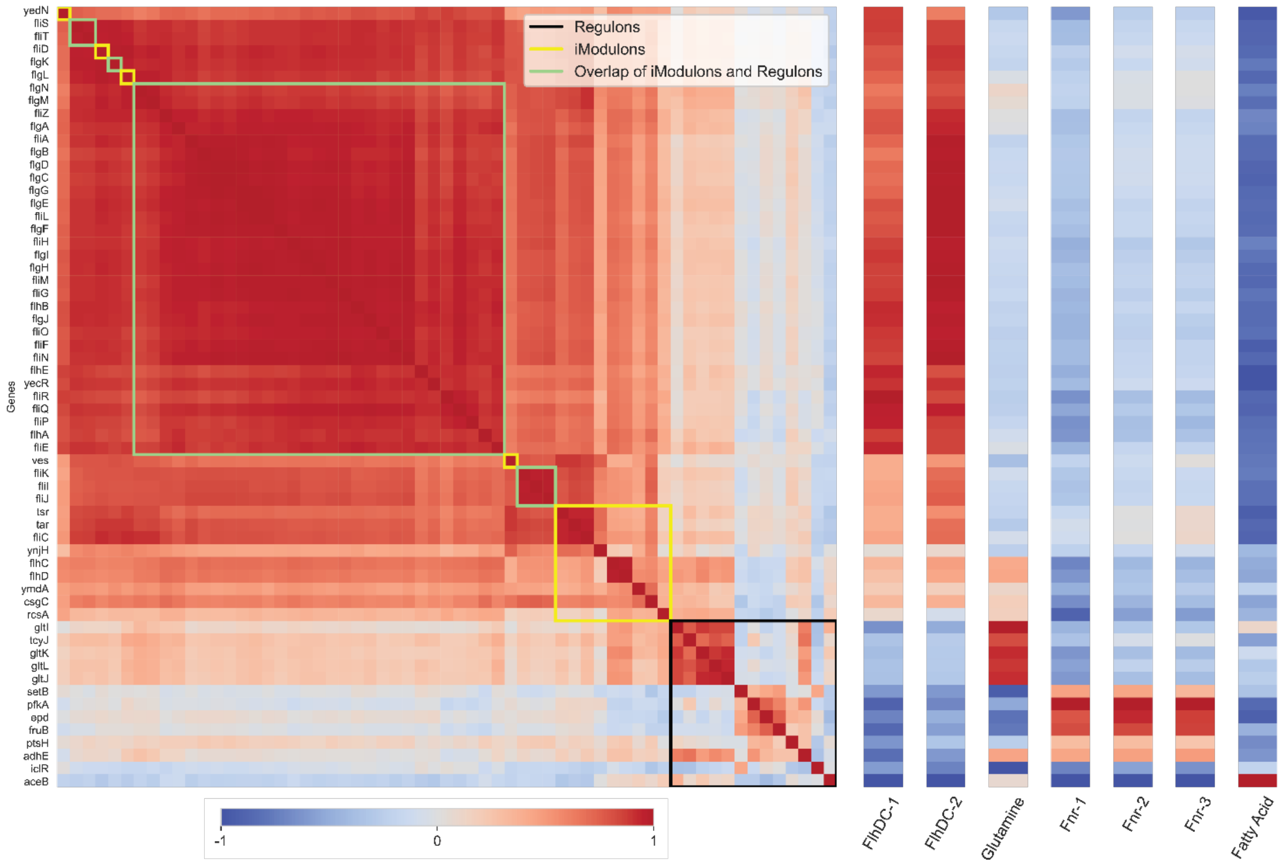


**Figure S30. Correlation heatmap of expression profiles for genes in FlhDC regulon and FlhDC-1/FlhDC-2 iModulon.**

X-axis and Y-axis both represent genes. The black block highlights genes exclusive to the FlhDC regulon, the yellow block indicates genes unique to the FlhDC-1/FlhDC-2 iModulon, and the green block highlights genes shared by both the FlhDC-1/FlhDC-2 iModulon and the FlhDC regulon. The right part has the correlation between the activities of the iModulons and gene expression profiles shown. FlhDC-1, FlhDC-2, Glutamine, Fnr-1, Fnr-2, Fnr-3, and Fatty Acid are plotted here.

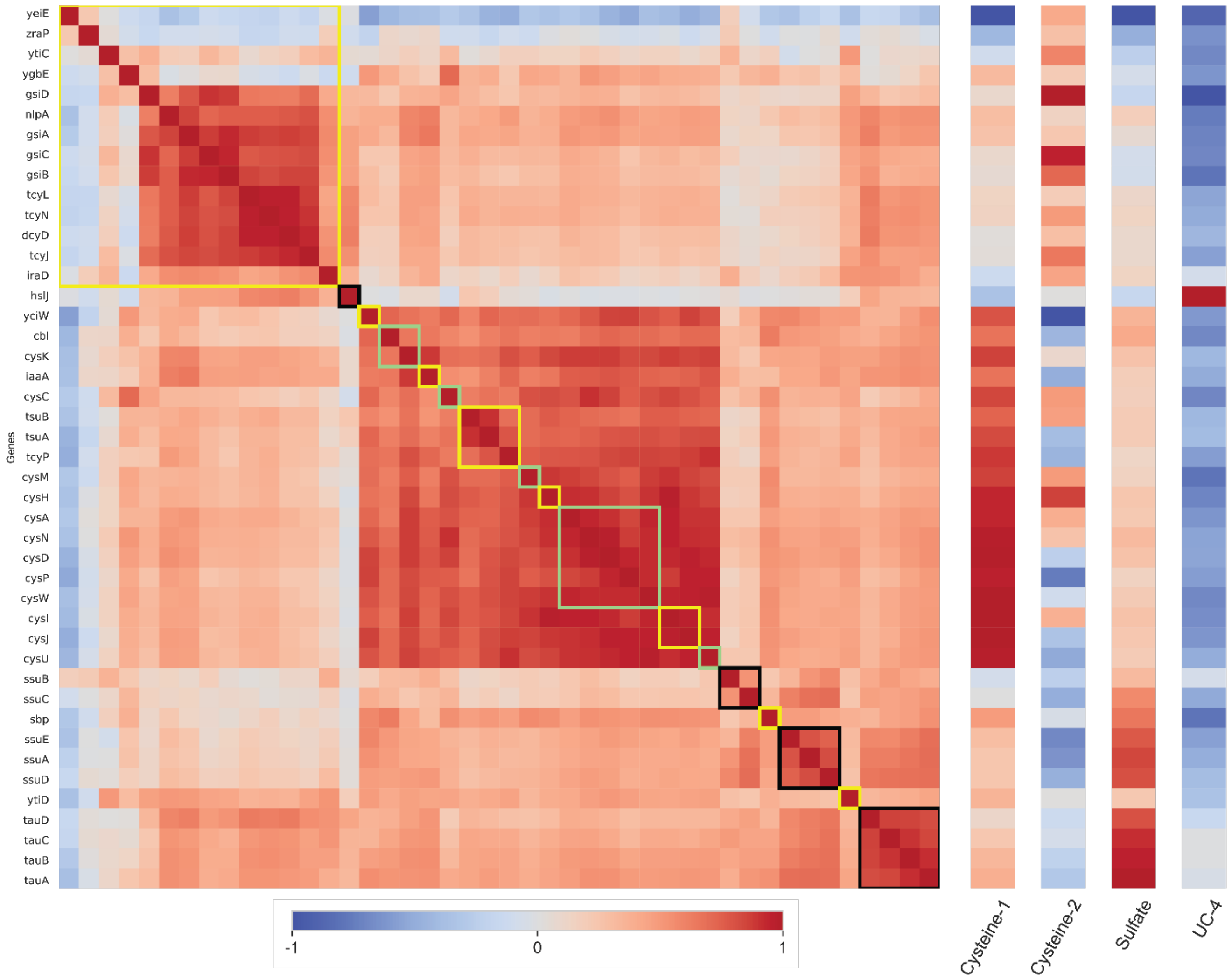


**Figure S31. Correlation heatmap of expression profiles for genes in CysB regulon and Cysteine-1/Cysteine-2 iModulon.**

X-axis and Y-axis both represent genes. The black block highlights genes exclusive to the CysB regulon, the yellow block indicates genes unique to the Cysteine-1/Cysteine-2 iModulon, and the green block highlights genes shared by both the Cysteine-1/Cysteine-2 iModulon and the CysB regulon. The right part has the correlation between the activities of the iModulons and gene expression profiles shown. Cysteine-1, Cysteine-2, Sulfate, and UC-4 are plotted here.

# Reference


1. Lacramioara Bintu, Nicolas E Buchler, Hernan G Garcia, Ulrich Gerland, Terence Hwa, Jan´e Kondev, and Rob Phillips. Transcriptional regulation by the numbers: models. Current opinion in genetics & development, 15(2):116–124, 2005.
2. Lacramioara Bintu, Nicolas E Buchler, Hernan G Garcia, Ulrich Gerland, Terence Hwa, Jan´e Kondev, Thomas Kuhlman, and Rob Phillips. Transcriptional regulation by the numbers: applications. Current opinion in genetics & development, 15(2):125–135, 2005.
3. Huynh-Thu, V. A., Irrthum, A., Wehenkel, L. & Geurts, P. Inferring regulatory networks from expression data using tree-based methods. *PLoS One* **5**, (2010).
4. Langfelder, P. & Horvath, S. WGCNA: an R package for weighted correlation network analysis. *BMC Bioinformatics* **9**, 559 (2008).
5. Fu, L. & Medico, E. FLAME, a novel fuzzy clustering method for the analysis of DNA microarray data. *BMC Bioinformatics* **8**, 3 (2007).
6. Li, G., Ma, Q., Tang, H., Paterson, A. H. & Xu, Y. QUBIC: a qualitative biclustering algorithm for analyses of gene expression data. *Nucleic Acids Res.* **37**, e101 (2009).
7. Bergmann, S., Ihmels, J. & Barkai, N. Iterative signature algorithm for the analysis of large-scale gene expression data. *Phys. Rev. E Stat. Nonlin. Soft Matter Phys.* **67**, 031902 (2003).
8. Wold, S., Esbensen, K. & Geladi, P. Principal component analysis. *Chemometrics Intellig. Lab. Syst.* **2**, 37–52 (1987).
9. Artac, M., Jogan, M. & Leonardis, A. Incremental PCA for on-line visual learning and recognition. in *2002 International Conference on Pattern Recognition* vol. 3 781–784 vol.3 (IEEE, 2002).
10. Yao, F., Coquery, J. & Lê Cao, K.-A. Independent Principal Component Analysis for biologically meaningful dimension reduction of large biological data sets. *BMC Bioinformatics* **13**, 24 (2012).
11. Hyvärinen, A. Fast and robust fixed-point algorithms for independent component analysis. *IEEE Trans. Neural Netw.* **10**, 626–634 (1999).
12. Wang, Z., Gaynanova, I., Aravkin, A. & Risk, B. B. Sparse Independent Component Analysis with an Application to Cortical Surface fMRI Data in Autism. *J. Am. Stat. Assoc.* 1–13.
13. Lee, D. D., Sebastian, H. & Seung, Y. Algorithms for non-negative matrix factorization. *Adv. Neural Inf. Process. Syst.* 556–562 (2000).
14. Rummel, R. J. *Applied Factor Analysis.* (Northwestern University Press, 1988).
15. Olshausen, B. A. & Field, D. J. Sparse coding with an overcomplete basis set: a strategy employed by V1? *Vision Res.* **37**, 3311–3325 (1997).
16. Liao, J. C. *et al.* Network component analysis: reconstruction of regulatory signals in biological systems. *Proc. Natl. Acad. Sci. U. S. A.* **100**, 15522–15527 (2003).
17. Badia-I-Mompel, P. *et al.* decoupleR: ensemble of computational methods to infer biological activities from omics data. *Bioinform. Adv.* **2**, vbac016 (2022).
